# Cyclization *vs.* Cyclization/Dimerization in *o*-Amidostilbene Radical Cation Cascade Reactions: The Amide Question

**DOI:** 10.3390/molecules16097267

**Published:** 2011-08-25

**Authors:** Chin Hui Kee, Azhar Ariffin, Khalijah Awang, Ibrahim Noorbatcha, Koichi Takeya, Hiroshi Morita, Chuan Gee Lim, Noel Francis Thomas

**Affiliations:** 1 Department of Chemistry, Faculty of Science, University of Malaya, 50603 Kuala Lumpur, Malaysia; 2 Department of Biotechnology Engineering, Faculty of Engineering, International Islamic University Malaysia, 53100 Kuala Lumpur, Malaysia; 3 Department of Pharmacognosy, Tokyo University of Pharmaceutical and Life Science, 1432-1 Horinouchi, Hachioji, Tokyo 192-0392, Japan; 4 Faculty of Pharmaceutical Sciences, Hoshi University, Shinagawa-ku, Tokyo 142-8501, Japan; 5 Environmental and Bioprocess Technology Centre, SIRIM Berhad, 40000 Shah Alam, Malaysia

**Keywords:** stilbene, indoline, bisindoline, FeCl_3_

## Abstract

The *n*-butyramido, isobutyramido, benzamido, and furancarboxamido functions profoundly modulate the electronics of the stilbene olefinic and NH groups and the corresponding radical cations in ways that influence the efficiency of the cyclization due presumably to conformational and stereoelectronic factors. For example, isobutyramido- stilbene undergoes FeCl_3_ promoted cyclization to produce only indoline, while *n*-butyramidostilbene, under the same conditions, produces both indoline and bisindoline.

## 1. Introduction

A large number of biologically active compounds incorporate indole and indoline moieties, particularly those that possess C-2 phenyl or heteroaryl moieties [1]. A vast array of creative approaches to the indoline ring system have been reported recently, for example the 1,6-H transfer followed by the 5-*exo*-trigonal ring closure tactic that exploits *o*-nitrobenzaldehyde [2]; the 5-*endo*-trigonal ring closure of *N*-(*o*-bromophenyl)ene carbamates [3]; intramolecular carbolithiation [4]; reduction of indoles [5] and the thermal reaction of *N*-allylaniline and various alkoxyamines [6]. Creative bisindoline construction is also increasing in importance as recent developments clearly indicate. For example, the highly efficient successive amide transfer approach leading to C2 symmetric bisindolines [7]; diaza-Cope rearrangement resulting in C3-C3’ bisisoindolines [8] and the [CoCl(PPh_3_)_3_] mediated indolyl bromide homolyisis/dimerisation [9].

**Scheme 1:**
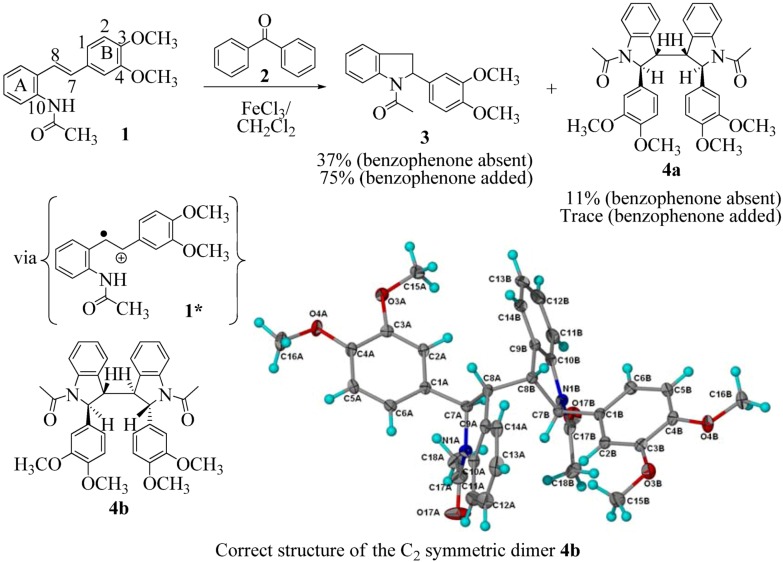
Oxidative coupling of 3,4-dimethoxy-acetamido stilbene (**1**).

In 2004, we described the then unprecedented FeCl_3_-promoted acetamidostilbene radical cation 1* cascade culminating in the indoline **3** and bisindoline **4a** [10]. A subsequent X-ray analysis of the bisindoline has shown that the correct structure should be the C_2_ symmetric dimer **4b **(Scheme 1) [11]. Four years later, we published the unexpected discovery that benzophenone (**2**) was capable of producing ca. a doubling of the yield of the indoline with concomittant suppression of bisindoline **4** formation (Scheme 1) [12]. The benzophenone was almost recovered always quantitatively. We proposed a Fe^3+^ benzophenone ketyl radical/stilbene radical cation catalytic cycle in that paper. Enhancement of indoline formation by an “internal agent” or via structural modification of the stilbene starting material was not envisaged.

The transformation shown in Scheme 1 proceeds via the radical cation (1*) followed by cyclization through the amidic NH to yield the indolyl radical and hence the products **3** and **4**. One question that we have not addressed is the relationship between the amide structure and the efficiency of the cyclization.

An *n*-butyramide (in place of the acetamide in Scheme 1) would be expected, on steric grounds, to engage the C(7) benzylic cation with greater difficulty than stilbene **1**. Cyclization (via FeCl_3_) in the case of the isobutyramidostilbene would constitute an even greater steric impediment to indoline formation. Would the furan carboxamido and benzamido moieties undergo cyclization with comparable efficiency or are there delicate conformational factors at work here?

We reported last year full spectroscopic details of several novel stilbenes that were prepared by the Heck procedure [13]. The amido NH in stilbene **6** is more shielded than **5 **(Table 1), which suggests that the amide in **6** should be somewhat more nucleophilic (under neutral or slightly acidic conditions). 

**Scheme 2:**
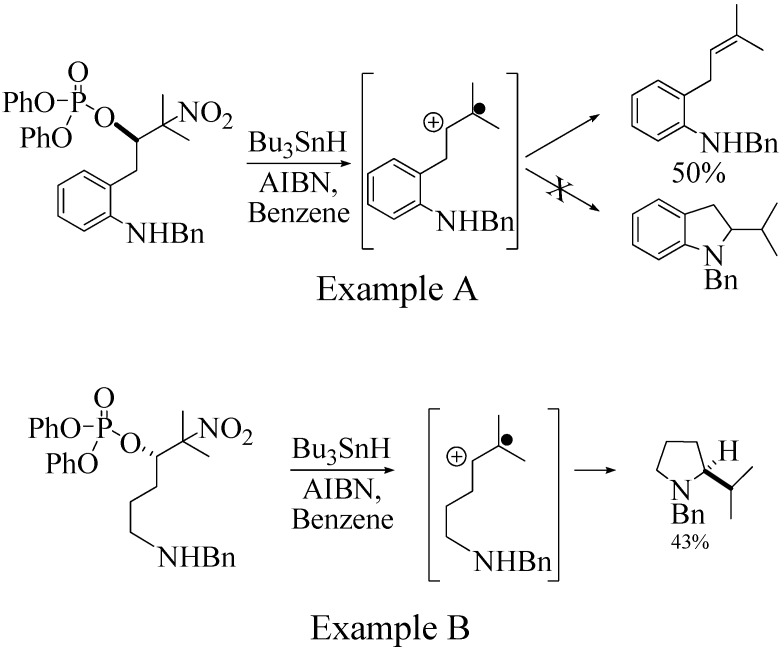
Examples of Crich’s alkene radical cation cyclizations [14].

On the other hand, the NMR spectra also reveal that the furancarboxamido stilbene **11** and the benzamidostilbene **7** possess even more deshielded NHs compared to stilbenes **6** and **5**. It could be argued, all other things being equal, that this significant withdrawal of electron density away from the amide NH could reduce the nucleophilicity of the amide group and thus adversely affect cyclization of the radical cation, in contrast to the cyclization of **5** or **6**. It is significant that Crich’s heterocyclization of a non-oxidatively derived non-stilbene radical cation fails in the case of A but is successful in the case of B (see Scheme 2). The different cyclization outcomes for A and B appear to be due to the severely reduced nucleophilicity of the benzylaniline in A compared to B.

Another interesting observation relates to the olefinic multiplets. For the furancarboxamido stilbenes **9**-**12**, the C(8)-H proton is more deshielded than the C(7)-H proton, the one noticeable exception being stilbene **9**, where the C(7)-H is significantly more deshielded. This might mean that exposure of stilbene **9** to FeCl_3_ could produce a radical cation in which the positive charge was now at C(8) rather than C(7) (in contrast to the usual pattern). The chemical shifts discussed above may be depicted as shown in Table 1.

**Table 1:** ^1^H-NMR [400MHz, *δ*H (*J*, Hz)] of stilbenes **5**-**12** in CDCl_3_.

Entry	Stilbene	C(7)-H (*J*, Hz)	C(8)-H (*J*, Hz)	N-H (*J*, Hz)
**1**	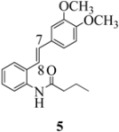	**6.85**	**6.95**	**7.56**
**2**	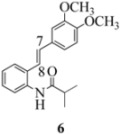	**6.90**	**6.97**	**7.31**
**3**	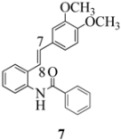	**6.93**	**7.04**	**8.03**
**4**	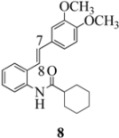	**6.91**	**6.97**	**7.21**
**5**	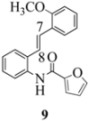	**7.40**	**7.29**	**8.25**
**6**	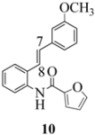	**7.02**	**7.22**	**8.17**
**7**	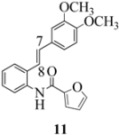	**6.96**	**7.07**	**8.19**
**8**	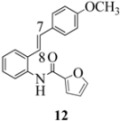	**7.00**	**7.09**	**8.17**

In order to unveil structural (electronic) and possible conformational influences on our radical cation cascade cyclization/dimerization, experiments were performed as described in the next section. We included stilbene **9** in our study to investigate the possibility of mesomeric stabilization by the *ortho*-methoxy substituent of the C(7) carbenium ion with the unpaired electron at C(8). Stilbene **10** was included to confirm an observation first reported in 2009 [15] where a *meta*-methoxy substituent directs radical cation formation in a way that is opposite to that observed with a *para*-methoxy substituent. We included stilbene **12** to examine the implications of having 4-methoxy substituent (rather than 3,4-dimethoxy substitution) on the formation of the radical cation.

## 2. Results and Discussion

### 2.1. FeCl_3_ Promoted Syntheses of Indolines

Eight carboxamidostilbenes were constructed by the Heck protocol [13]. These stilbenes were then subjected to our FeCl_3_ reaction conditions as shown below (Table 2). The resulting observations demand a more complex explanation than we had anticipated based on previous experience [10]. 

**Table 2:** FeCl_3_ promoted syntheses of indolines and bisindolines. 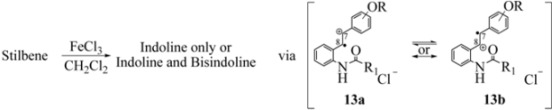

Entry	Stilbene	Indoline (Yield)	Bisindoline (Yield)
**1**	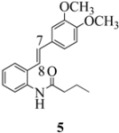	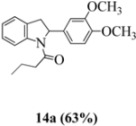	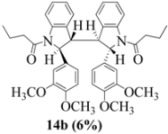
**2**	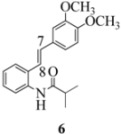	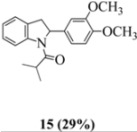	-
**3**	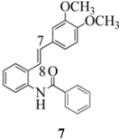	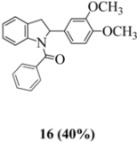	-
**4**	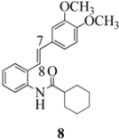	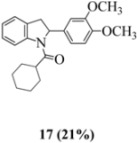	-
**5**	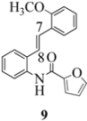	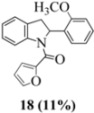	-
**6**	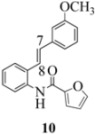	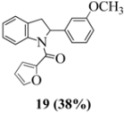	-
**7**	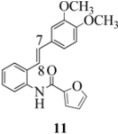	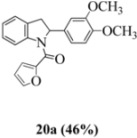	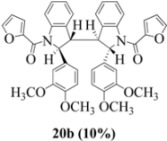
**8**	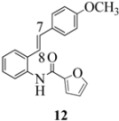	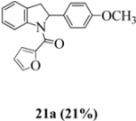	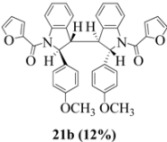

Oxidation of the stilbene **5** with FeCl_3_ gave rise to indoline **14a** (63%) and bisindoline **14b** (6%) (*inthe absence of benzophenone*) via radical cation **13a **(in contrast to the transformation in Scheme 1) an unexpected result. We had previously reported that the 10-acetamido-4-methoxystilbene on exposure to FeCl_3_ produced the bisindoline in 24% yield and the indoline in 16% yield [15]. This “4-methoxy effect” is also seen in stilbene **12** (Entry 8, Table 2) where the proportion of bisindoline is significantly increased. In the present study, replacement of butyramide (**5**) with isobutyrylamide (**6**) resulted in a lowering of the yield of indoline **15** to 29%. Bisindoline formation was not observed, a result that is all the more remarkable when compared to stilbene **12** (Entry 8, Table 2).

### 2.2. Effect of Amide Structure on the Radical Cyclization Leading to Indoline Formation

**Scheme 3:**
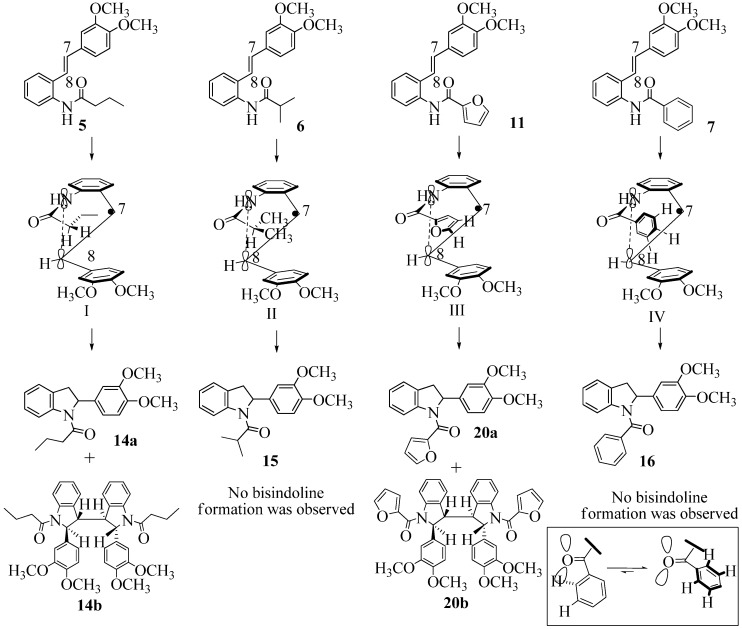
Early transition state pictures for radical cyclization leading to indoline formation.

The very different behaviour of **5** and **6** on exposure to FeCl_3_ is readily explained by the early transition state pictures **I** and **II** (Scheme 3) where the increase steric bulk of the isobutyrylamide renders cyclization more difficult, hence the lower yield of **15 **(29%). A similar explanation would account for the low yield of indoline **17** from stilbene **8**.

With respect to stilbenes **7** and **11**, cyclization proceeding via radical cation **13a** leading to **16** and **20a** respectively, is slightly more favoured for stilbene **11** (furancarboxamide) than for stilbene **7** (benzamide) on stereoelectronic grounds [16], as the transition state pictures **III** and **IV** demonstrate (Scheme 3). We believe that the coplanar orientation of furan and carbonyl groups means that cyclization experiences less resistance in the case of **11**. This coplanarity seems less likely for stilbene **7** (see III and IV, Scheme 3). The stereoelectronic factor also helps to explain the fact that bisindoline formation (**20b** and **21b**) is observed with stilbenes **11** and **12** but not with stilbene **7** (see discussion in Section 2.3 and Scheme 10). In the case of stilbene **9**, cyclization is made more difficult (and hence the significantly reduced yield) because it must proceed via radical cation **13b**. It is noteworthy that the C(8) carbon of **9**, in this special case, is more shielded than the C(7) (compared to the other furancarboxamidostilbenes) because of the directional nature and electron withdrawing tendency of the 2-methoxy lone electron pairs [13]. In the case of stilbene **10**, radical cation **13b** is again the crucial intermediate (see Ref. [10] for the similar case of 10-acetamido-3-methoxystilbene) [15]. It may be worth emphasising that the FeCl_3_ oxidation of stilbenes **9** and **10** proceed via a different radical cations (analogous to **13b**) whereas stilbene **12** proceeds via radical cation **13a** (see Table 2). This is because stilbene **12** on oxidation produces a radical cation in which the positive charge is at C(7) as a result of mesomeric stabilization from a *para*-methoxy substituent. By contrast in stilbene **10** where the methoxy group is *meta*, the position of the positive charge changes to C(8) because of superior mesomeric stabilization which comes from the amide nitrogen lone pair in the *ortho* position (*i.e.*, a C-10 amido group). In the case of stilbene 9 where mesomeric stabilization of a C(7) carbocation (in the radical cation) might have been expected, we observed a unique *ortho*-methoxy lone pair effect which dramatically reverses the position of radical and positive charge. The positive charge is now at C(8) rather than at C(7). This electronic factor is seen even in the NMR of the stilbenes (see Table 1). Notice that remarkably in stilbene **9** the C(7) proton is substantially more deshielded than C(8). The phenomena described above underpin the mechanistic interpretation discussed in Scheme 11, Scheme 12 and Scheme 13. It is significant that in contrast to our first report (Scheme 1) where the bisindoline **4A** was presented in the *meso* arrangement, in this report all bisindolines are designated as racemic. The reason for this is explained in Section 2.3.

### 2.3. Dimerization Pathways and PM6 Calculations for the Minor Products

The basis for the observed stereoselectivity for each of the bisindoline minor products (**14b**, **20b** and **21b**) is explained below. There are four diastereomeric possibilities. The PM6 calculations (MOPAC2009) are provided in Table 3, Table 4 and Table 5.

**Table 3:** PM6 calculation for **14b**.

Stereoisomer	Symmetryelement	Heat of Formation (kcal/mol)	H2-H3	H2*-H3*	H3-H3*
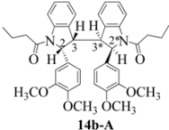	C_2_ axis	−154.22	19.15	346.41	156.58
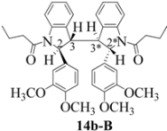	C_2_ axis	−162.78	250.79	250.79	171.63
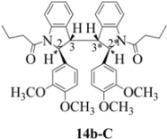	Plane (*Meso*)	−163.46	240.71	111.62	298.43
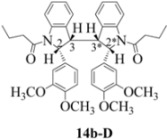	Plane (*Meso*)	−133.96	310.52	324.60	24.01

**Table 4:** PM6 calculation for **20b**.

Stereoisomer	Symmetryelement	Heat of Formation (kcal/mol)	H2-H3	H2*-H3*	H3-H3*
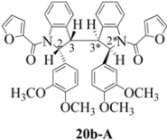	C_2_ axis	−116.85	348.44	348.44	155.06
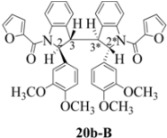	C_2_ axis	−123.76	241.19	241.17	120.43
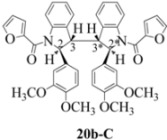	Plane (*Meso*)	−126.41	−111.73	119.11	61.86
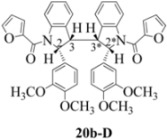	Plane (*Meso*)	−115.76	352.90	26.21	65.36

**Table 5:** PM6 calculation for **21b**.

Stereoisomer	Symmetryelement	Heat of Formation (kcal/mol)	H2-H3	H2*-H3*	H3-H3*
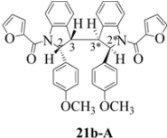	C_2_ axis	−48.59	340.27	340.27	115.07
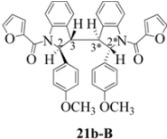	C_2_ axis	−55.04	247.75	247.23	172.63
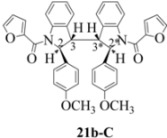	Plane (*Meso*)	−56.28	240.70	111.61	298.43
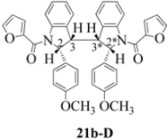	Plane (*Meso*)	−43.83	12.48	26.33	69.76

**Scheme 4:**
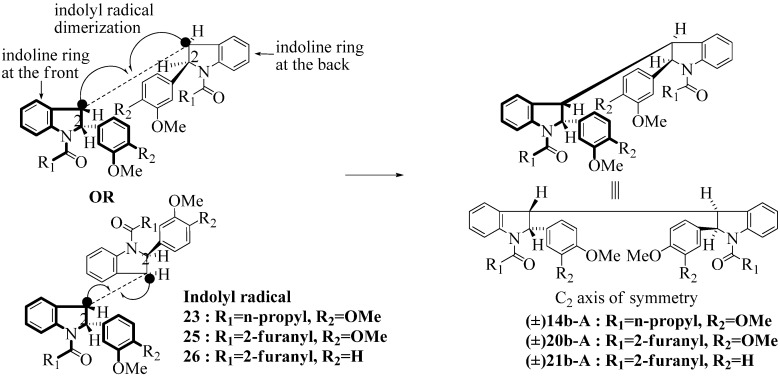
Dimerization pathways leading the stereoisomers **(±)14b-A**, **(±)20b-A** and **(±)21b-A**.

**Scheme 5:**
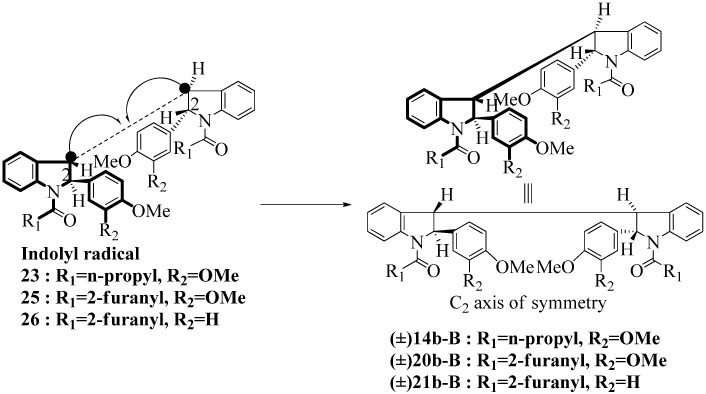
Dimerization pathways leading the stereoisomers **(±)14b-B**, **(±)20b-B** and **(±)21b-B.**

**Scheme 6:**
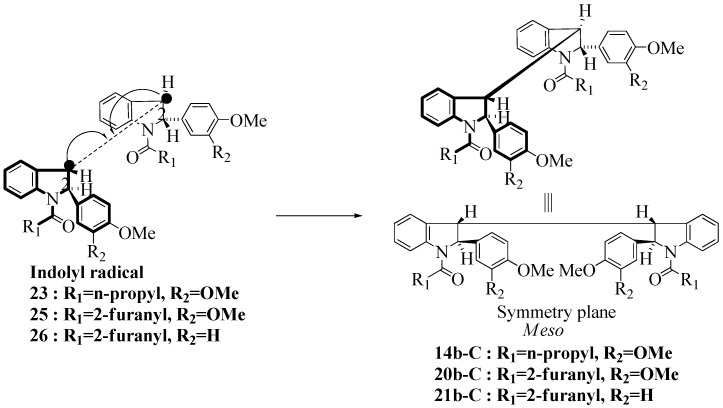
Dimerization pathways leading the stereoisomers **14b-C**, **20b-C** and **21b-C.**

**Scheme 7:**
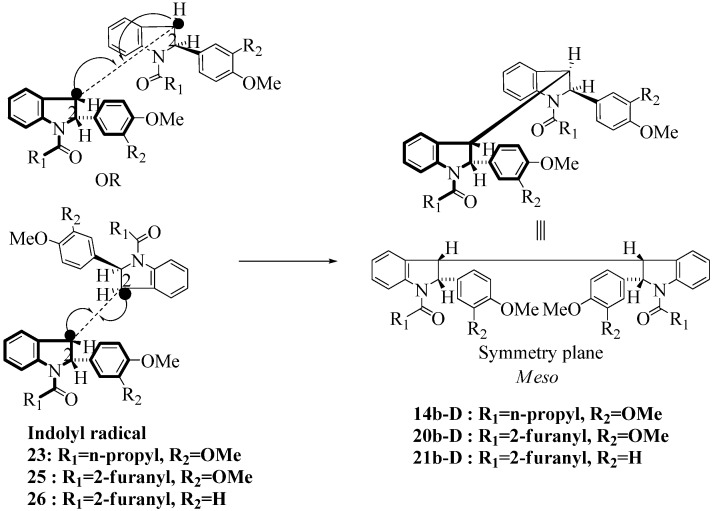
Dimerization pathways leading the stereoisomers **14b-D**, **20b-D** and **21b-D.**

These calculations (the thermodynamic argument) lead to the same conclusions as the mechanistic analysis of dimerization pathways for the various indolyl radicals (the kinetic argument) see Scheme 4, Scheme 5, Scheme 6 and Scheme 7.

**Scheme 8:**
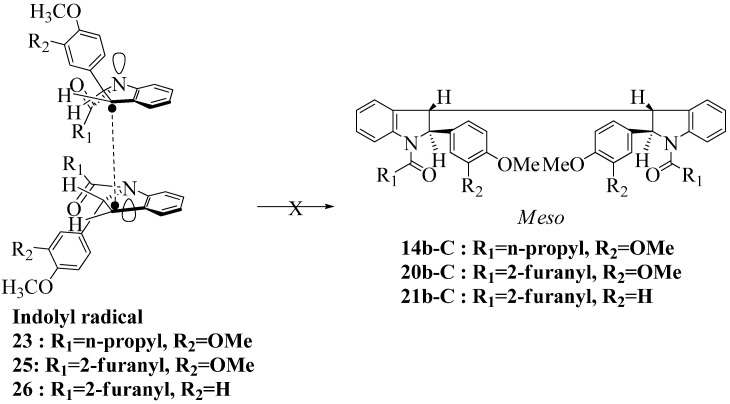
Another perspective on dimerization pathways leading to the stereoisomer **14b-C**, **20b-C** and **21b-C**.

**Scheme 9:**
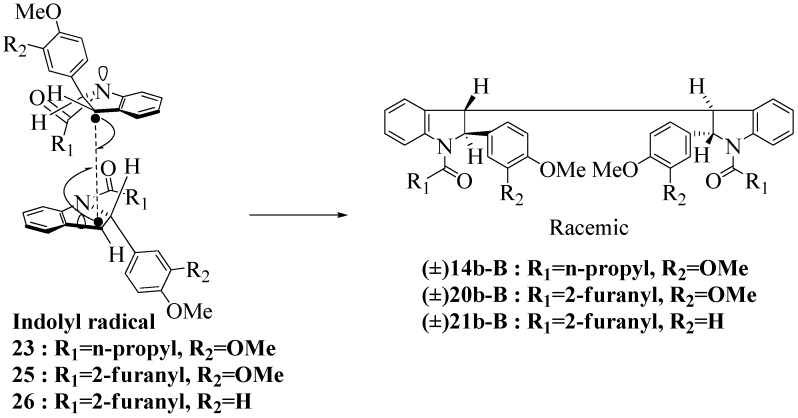
Another perspective on dimerization pathways leading to the stereoisomer **(±)14b-B**, **(±)20b-B** and **(±)21b-B**.

The arguments that distinguish **(±)14b-B** and *meso* **14b-C** (Table 3) are more finely balanced as these two diastereoisomers are closer in energy (-162.78 and -163.46 kcal/mol). This closeness is also reflected in the fact that in examining dimerization pathways for the indolyl radicals (Scheme 5 and Scheme 6), dimerization appears to be equally feasible in both cases. In comparing the early transition state pictures in Scheme 8 and Scheme 9, the approach of indolyl radicals in Scheme 9 would have the effect of keeping the “phenyl” region of the indoline rings as far apart as posible, as well as possibly minimizing interactions between the amide moieties (see Scheme 8). This would appear to lead to the conclusion that the correct stereostructures are **(±)14b-B**, **(±)20b-B** and **(±)21b-B** respectively (see Scheme 9 and Table 2).

One additional point of some importance relates to the PM6 calculations. Although **(±)14b-B** is slightly higher in energy, the dihedral angles in this case are consistent with the symmetrical nature of the molecule (Table 3) and is therefore to be preferred over the *meso* form **14b-C** (Scheme 8). These arguments fully justify the exploitation of PM6 calculations for the elucidation of stereochemical problems inherent in reactions of this type.

Exactly analogous arguments can be used to justify the presentation of bisindolines **20b** and **21b **which carry furancarboxamide moieties, as their racemic modifications (Table 2, Table 4 and Table 5). For **20b-A**, **20b-D**, **21b-A** and **21b-D** are readily ruled out on energetic grounds and this thermodynamic argument is again amply vindicated by the dimerization pathways for the indolyl radicals. Consideration of indolyl radical dimerization pathways and thermodynamic arguments favour formation of **(±)20b-B** and **20b-C** (Scheme 5 and Scheme 6) and **(±)21b-B** and **21b-C** (Scheme 5 and Scheme 6). 

**Scheme 10:**
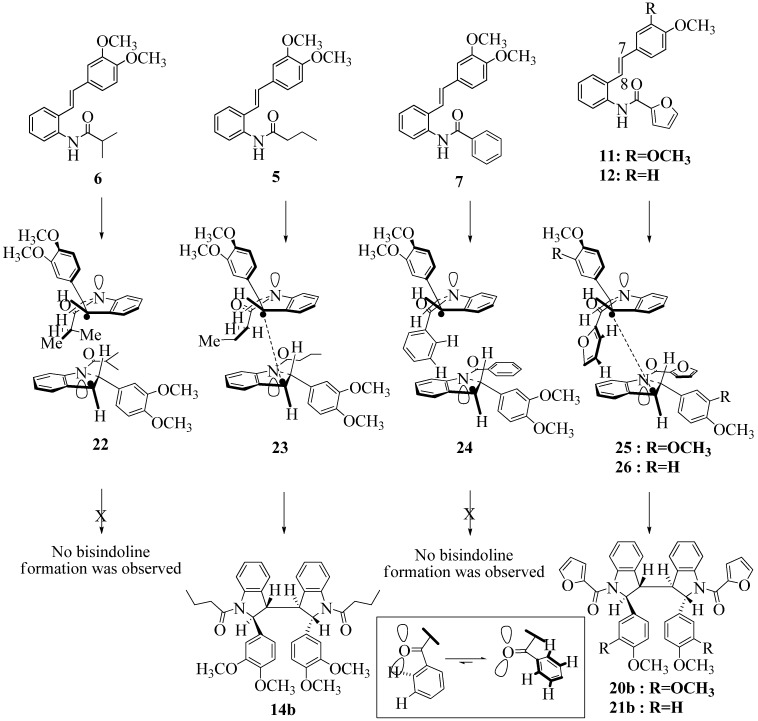
Effect of amide structure on the dimerization of indolyl radical **22-26 **leading to bisindoline formation.

However careful scrutiny of the dimerization pathways as previously described (Scheme 9 and Scheme 10) inevitably leads to the conclusion that the correct stereostructures are **(±)20b-B** and **(±)21b-B**. These conclusion are supported by the X-ray data for the bisindoline depicted in Scheme 1 and confirm that the bisindoline has the structure **(±)4b** (and not **4a** as originally reported by us).

With respect to bisindoline formation, it would seem that in the presumably early transition states involving the corresponding indolyl radicals **22** and **23** (Scheme 10), the methyl group of the isobutryl moiety in **22** is able to block dimerization so that no bisindoline is obtained. By contrast indolyl radical **23** by virtue of possessing an *n*-butyrylamido group has only the much smaller H in that position and dimerization is not impeded. 

**Scheme 11:**
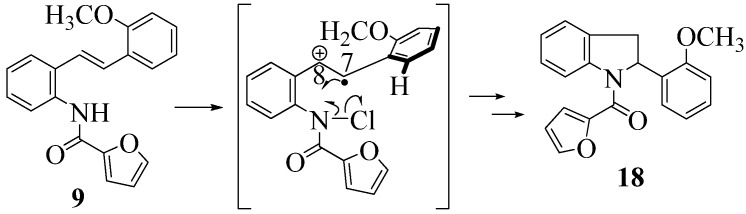
The *ortho*-methoxy effect.

In the case of the **9** (Scheme 11), the attempted dimerization fails in spite of the fact that one would have expected a radical cation intermediate analogous to **13a** (Table 2). The reduced nucleophilicity of this NH and the reduced electron density in the olefinic bond may well account for the low yield of the indoline **18** (Scheme 10) (see our previous discussion on NMR spectroscopic characteristics for the eight stilbenes) [13].

**Scheme 12:**
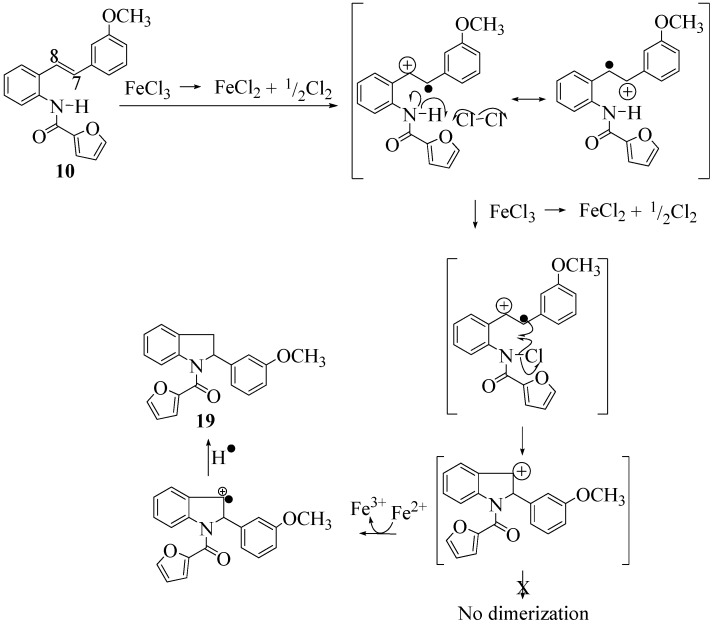
Mechanistic interpretation I.

It is interesting to note that in **9** the C7-H (*δ* 7.40) is more deshielded than the C8-H (*δ* 7.29) in contrast to the other furan carboxamidostilbenes **10**, **11** and **12**. This would have the effect of reversing the position of the radical and cation (formed after oxidation) where the removal of an electron from C8 would be preferred. 

**Scheme 13:**
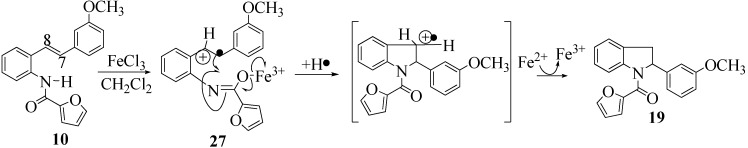
Mechanistic interpretation II.

The failure of dimerization in the case of **10** is due to the formation of the radical cation analogous to **13b** (Table 2) and Scheme 12. The formation of this radical cation is related to the absence of a paramethoxy group (in **10**) that could stabilize a C7 cation and as a result the cation is formed preferentially at C8. Ring closure proceeds possibly via homolysis of an N-Cl bond leading to the nitrogen centred radical as shown in Scheme 12 or alternatively, via the Fe^3+^ azaenolate (**27**). Reduction of the indolyl cation (whose formation renders dimerization impossible) to the indolyl radical cation and H• uptake leads to the indoline. This pathway is shown in Scheme 13.

## 3. Experimental

### 3.1. General

Unless otherwise noted, materials were purchased from commercial suppliers and used without purification. THF was freshly distilled from calcium hydride. DMF was dried over 4Å molecular sieves (Sigma-Aldrich) prior to use. Column chromatography was performed using Merck silica gel (0.040–0.063 mm). For thin layer chromatography, Merck TLC aluminum sheets (silica gel 60 F_254_) were used; centrifugal chromatography, Merck silica gel 60 PF_254_ containing gypsum were used. Infrared spectra were recorded on a Perkin Elmer FTIR Spectrum RX-1 spectrometer at wavenumbers from 4000–400 cm^−1^. Nuclear magnetic resonance (NMR) spectra were obtained on a JEOL JNM-LA 400 and JEOL ECA-400 instrument. Spectra are reported in units of ppm on the scale, relative to chloroform and the coupling constants are given in Hz. Ultraviolet (UV) spectra were recorded from 190–400 nm, in methanol, on a Shimadzu UV-Visible Spectrophotometer 1650. Mass spectra were measured using an Agilent 6530 Accurate-Mass Q-TOF LC/MS system. Detailed experimental procedures for the synthesis of stilbenes **7**-**14** and starting materials have been reported elsewhere [13].

### 3.2. General Procedure for the FeCl_3_ Oxidative Coupling

Stilbene (1 equiv) was dissolved in CH_2_Cl_2_ (25 mL). ‘Anhydrous’ FeCl_3_ (2-3 equiv) was added to the mixture under nitrogen. The mixture was stirred at room temperature and monitored by TLC. After the consumption of the stilbene, the mixture was diluted with aqueous ammonium chloride solution and extracted with ethyl acetate (3 × 25 mL). The combined organic fractions were dried over anhydrous sodium sulphate. Purification of the crude product by column chromatography afforded the desired products. Semi-empirical molecular orbital calculations were performed to optimise the geometry of the molecules using the PM6 method [17] included in MOPAC2009 [18]. 

#### 3.2.1. 1-(2-(3,4-Dimethoxyphenyl)indolin-1-yl)butan-1-one (**14a**)

Yield 0.30 g (63%); *ν*_max_/cm^−1^ (NaCl): 2961, 1659, 1516, 1480, 1463, 1401, 1262, 1139, 1027, 754; λ_max_ (MeOH)/nm: 237, 253, 281; ^1^H-NMR (400 MHz; CDCl_3_) *δ*_H_ 8.33 (d, *J* = 7.6 Hz, H-8, 1H), 7.24 (t, *J* = 7.8 Hz, H-7, 1H), 7.12 (d, *J* = 6.4 Hz, H-5, 1H), 7.03 (t, *J* = 7.6 Hz, H-6, 1H) , 6.76 (d, *J* = 8.2 Hz, H-5', 1H), 6.68 (d, *J* = 8.2 Hz, H-6', 1H), 6.64 (s, H-2', 1H), 5.36 (d, *J* = 7.3 Hz, H-2, 1H), 3.83 (s, OCH_3_, 3H), 3.77 (s, OCH_3_, 3α, 4H), 2.94 (d, *J* = 16.5 Hz, H-3β, 1H), 2.09-2.36 (m, H-2", CH2, 2H), 1.67 (s, H-3", CH2, 2H), 0.84 (s, H-4", CH3, 3H); ^13^C-NMR (100 MHz; CDCl_3_) *δ*_C_ 172.0 (C-1"), 149.5 (C-3'), 148.5 (C-4'), 143.5 (C-9), 136.0 (C-1'), 129.3 (C-4), 127.8 (C-7), 125.1 (C-5), 124.0 (C-6), 117.2 (C-6'), 117.0 (C-8), 111.4 (C-5'), 108.0 (C-2'), 62.6 (C-2), 56.0 (OCH_3_), 55.9 (OCH_3_), 39.2 (CH_2_, C-3), 37.5 (CH_2_, C-2"), 18.2 (CH_2_, C-3"), 13.9 (CH3, C-4"); HRMS (+ESI) [M+H]^+^: 326.1754, C_20_H_24_NO_3_ requires 326.1756.

#### 3.2.2. 1,1'-2,2'- bis(3,4-Dimethoxyphenyl)- 3,3'- biindoline-1,1'-diyl dibutan-1-one (**(±)14b**)

Yield 0.06 g (6%); *ν*_max_/cm^−1^ (NaCl): 2961, 1666, 1516, 1462, 1398, 1255, 1140, 1027, 755 ; λ_max_ (MeOH)/nm: 237, 249, 281; ^1^H-NMR (400 MHz; CDCl_3_) *δ*_H_ 8.53 (d, *J* = 8.2 Hz, H-8, H-8*, 2H), 7.46 (t, *J* = 8.0 Hz, H-7, H-7*, 2H), 7.30 (d, *J* = 7.3 Hz, H-5, H-5*, 2H), 7.21 (t, *J* = 7.3 Hz, H-6, H-6*, 2H), 6.61 (d, *J* = 8.7 Hz, H-5', H-5'*, 2H), 6.21 (d, *J* = 7.8 Hz, H-6', H-6'*, 2H), 5.81 (s, H-2', H-2'*, 2H), 4.70 (s, H-2, H-2*, CH, 2H), 3.77 (s, 2 X OCH_3_, 6H), 3.61 (s, 2 X OCH_3_, 6H), 3.52 (s, H-3, H-3*, CH, 2H), 1.83-2.12 (m, H-2", H-2"*, CH2, 4H), 1.45-1.56 (m, H-3", H-3"*, CH_2_, 4H), 0.76 (t, *J* = 7.6 Hz, H-4", H-4"*, CH_3_, 6H); ^13^C-NMR (100 MHz; CDCl_3_) *δ*_C_ 172.4 (C-1", C-1"*), 149.4 (C-3', C-3'*), 148.3 (C-4', C-4'*), 144.3 (C-9, C-9*), 134.7 (C-1', C-1'*), 130.0 (C-4, C-4*), 129.4 (C-7, C-7*), 125.3 (C-5, C-5*), 124.5 (C-6, C-6*), 117.7 (C-8, C-8*), 115.9 (C-6', C-6'*), 111.3 (C-5', C-5'*), 107.5 (C-2', C-2'*), 63.4 (C-2, C-2*), 56.9 (C-3, C-3*), 55.9 (2 X OCH_3_), 55.8 (2 X OCH_3_), 37.1 (CH_2_, C-2", C-2"*), 17.7 (CH_2_, C-3", C-3"*), 13.8 (CH_3_, C-4", C-4"*); HRMS (+ESI) [M+H]^+^: 649.3276, C_40_H_44_N_2_O_6_ requires 649.3278.

#### 3.2.3. 1-(2-(3,4-dimethoxyphenyl)indolin-1-yl)-2-methylpropan-1-one (**15**)

Yield 0.13 g (29%); *ν*_max_/cm^−1^ (NaCl): 2964, 1653, 1516, 1479, 1263, 1138, 1026, 755; λ_max_ (MeOH)/nm: 210, 238, 253, 281; ^1^H-NMR (400 MHz; CDCl_3_) *δ*_H_ 8.36 (d, *J* = 5.5 Hz, H-8, 1H), 7.24 (t, *J* = 7.8 Hz, H-7, 1H), 7.11 (d, *J* = 7.3 Hz, H-5, 1H), 7.02 (t, *J* = 7.8 Hz, H-6, 1H) , 6.75 (d, *J* = 8.2 Hz, H-5', 1H), 6.68 (dd, *J* = 8.2 Hz, 1.8 Hz, H-6', 1H), 6.64 (s, H-2', 1H), 5.43 (d, *J* = 8.7 Hz, H-2, 1H), 3.82 (s, OCH_3_, 3H), 3.77 (s, OCH_3_, H-3α, 4H), 2.96 (d, *J* = 16.0, H-3β, 1H), 2.61 (s, H-2", 1H), 1.20 (d, *J* = 6.9 Hz, H-4", 3H), 0.87 (s, H-3", 1H); ^13^C-NMR (100 MHz; CDCl_3_) *δ*_C_ 177.0 (C-1"), 149.5 (C-3'), 148.5 (C-4'), 143.5 (C-9), 136.3 (C-1'), 129.4 (C-4), 127.8 (C-7), 125.0 (C-5), 124.1 (C-6), 117.3 (C-8), 117.1 (C-6'), 111.4 (C-5'), 108.0 (C-2'), 62.4 (C-2), 56.0 (2 x OCH_3_), 39.1 (CH_2_, C-3), 33.4 (CH, C-2"), 20.3 (CH_3_, C-4"), 19.1 (CH_3_, C-3"); HRMS (+ESI) [M+H]^+^: 326.1760, C_20_H_24_NO_3_ requires 326.1751.

#### 3.2.4. (2-(3,4-Dimethoxyphenyl)indolin-1-yl)(phenyl)methanone (**16**)

Yield 0.21 g (40%); *ν*_max_/cm^−1^ (NaCl): 2956, 1646, 1516, 1480, 1386, 1259, 1139, 1026, 755; λ_max_ (MeOH)/nm: 216, 267; ^1^H-NMR (400 MHz; CDCl_3_) *δ*_H_ 7.38 (t, *J* = 6.8 Hz, H-5", 1H), 7.17-7.31 (m, H-5, H-7, H-8, H-3", H-4", H-6", H-7", 7H), 7.06 (t, *J* = 7.3 Hz, H-6, 1H), 6.68 (d, *J* = 8.2 Hz, H-5', 1H) , 6.55 (d, *J* = 5.0 Hz, H-6', 1H), 6.42 (s, H-2', 1H), 5.37 (s, H-2, 1H), 3.81 (s, OCH_3_, 3H), 3.67-3.74 (m, OCH_3_, H-3α, 4H), 2.98 (dd, *J* = 16.2 Hz, 1.8 Hz, H-3β, 1H); ^13^C-NMR (100 MHz; CDCl_3_) *δ*_C_ 169.9 (C-1"), 149.0 (C-3'), 148.3 (C-4'), 143.2 (C-9), 137.1 (C-2"), 136.2 (C-1'), 130.7 (C-4), 130.0 (C-5"), 128.3 (C-4", C-6"), 127.6 (C-7), 127.0 (C-3", C-7"), 125.3 (C-5, C-8), 124.4 (C-6), 117.5 (C-6'), 111.3 (C-5'), 108.9 (C-2'), 64.3 (C-2), 55.9 (OCH_3_), 55.8 (OCH_3_), 38.5 (C-3); HRMS (+ESI) [M+Na]^+^: 382.1416, C_23_H_21_NNaO_3_ requires 382.1414.

#### 3.2.5. Cyclohexyl(2-(3,4-dimethoxyphenyl)indolin-1-yl)methanone (**17**)

Yield 0.13 g (12%); *ν*_max_/cm^−1^ (NaCl): 2930, 1655, 1516, 1479, 1403, 1265, 1138, 1027, 754; λ_max_ (MeOH)/nm: 216, 237, 254, 282; ^1^H-NMR (400 MHz; CDCl_3_) *δ*_H_ 8.35 (d, *J* = 6.4 Hz, H-8, 1H), 7.24 (t, *J* = 8.1 Hz, H-7, 1H), 7.11 (d, *J* = 6.6 Hz, H-5, 1H), 7.03 (t, *J* = 7.4 Hz, H-6, 1H) , 6.76 (d, *J* = 8.3 Hz, H-5', 1H), 6.70 (dd, *J* = 8.2 Hz, 1.7 Hz, H-6', 1H), 6.65 (s, H-2', 1H), 5.42 (d, *J* = 7.6 Hz, H-2, 1H), 3.83 (s, OCH_3_, 3H), 3.77 (s, OCH_3_, H-3α, 4H), 2.97 (d, *J* = 15.8, H-3β, 1H), 2.34 (s, H-2", 1H), 1.20-1.89 (m, 5 x CH_2_, 10H); ^13^C-NMR (100 MHz; CDCl_3_) *δ*_C_ 175.9 (C-1"), 149.3 (C-3'), 148.4 (C-4'), 143.5 (C-9), 136.5 (C-1'), 129.4 (C-4), 127.7 (C-7), 124.8 (C-5), 123.7 (C-6), 117.1 (C-8, C-5'), 111.4 (C-6'), 108.0 (C-2'), 62.3 (C-2), 55.8 (2 x OCH_3_), 43.8 (CH, C-2"), 38.9 (CH_2_, C-3), 30.0, 28.7, 26.0, 25.6, 25.4 (5 x CH_2_); HRMS (+ESI) [M+H]^+^: 366.2065, C_23_H_27_NO_3_ requires 366.2064.

#### 3.2.6. Furan-2-yl(2-(2-methoxyphenyl)indolin-1-yl)methanone (**18**)

Yield 0.07 g (11%); *ν*_max_/cm^−1^ (NaCl): 3007, 1634, 1479, 1397, 1243, 1025, 832, 753; λ_max_ (MeOH)/nm: 280, 297; ^1^H-NMR (400 MHz; CDCl_3_) *δ*_H_ 8.33 (s, H-8, 1H), 7.36 (d, *J* = 1.8 Hz, H-5", 1H), 7.28 (t, *J* = 7.8 Hz, H-7, 1H), 7.17 (td, *J* = 7.8 Hz, 1.8Hz, H-4', 1H), 7.12 (d, *J* = 7.3 Hz, H-5, 1H), 7.06 (td, *J* = 7.6 Hz, 0.9 Hz, H-6, 1H), 6.98 (dd, *J* = 7.6 Hz, 1.8 Hz, H-6', 1H), 6.89 (d, *J* = 8.2 Hz, H-3', 1H), 6.80 (d, *J* = 3.2 Hz, H-3", 1H), 6.75 (t, *J* = 7.5 Hz, H-5', 1H), 6.33 (dd, *J* = 3.7Hz, 1.8 Hz, H-4", 1H), 6.31 (dd, *J* = 9.8 Hz, 1.4 Hz, H-2, 1H), 3.92 (s, OCH_3_, 3H), 3.76 (dd, *J* = 16.2 Hz, 10.1 Hz, H-3α, 1H), 2.90 (dd, *J* = 16.5 Hz, 1.4 Hz, H-3β, 1H); ^13^C-NMR (100 MHz; CDCl_3_) *δ*_C_ 158.2 (C-1"), 155.5 (C-2'), 147.8 (C-2"), 144.5 (C-5"), 143.7 (C-9), 131.6 (C-1'), 130.7 (C-4), 128.4 (C-4'), 127.6 (C-7), 125.2 (C-5), 125.0 (C-6'), 124.7 (C-6), 120.8 (C-5'), 118.1 (C-8), 116.5 (C-3"), 111.5 (C-4"), 110.5 (C-3'), 58.9 (C-2), 55.6 (OCH_3_), 38.2 (C-3); HRMS (+ESI) [M+Na]^+^: 342.1106, C_20_H_17_NNaO_3_ requires 342.1101.

#### 3.2.7. Furan-2-yl(2-(3-methoxyphenyl)indolin-1-yl)methanone (**19**)

Yield 0.04 g (38%); *ν*_max_/cm^−1^ (NaCl): 3006, 1634, 1479, 1397, 1286, 1048, 755; λ_max_ (MeOH)/nm: 281, 296; ^1^H-NMR (400 MHz; CDCl_3_) *δ*_H_ 8.24 (s, H-8, 1H), 7.39 (s, H-5", 1H), 7.27 (t, *J* = 8.2 Hz, H-7, 1H), 7.12-7.16 (m, H-5, H-5', 2H), 7.07 (t, *J* = 7.6 Hz, H-6, 1H), 6.91 (d, *J* = 3.7 Hz, H-3", 1H), 6.69-6.75 (m, H-2', H-4', H-6', 3H), 6.36 (dd, *J* = 3.4Hz, 1.8 Hz, H-4", 1H), 6.05 (dd, *J* = 9.6 Hz, 1.4 Hz, H-2, 1H), 3.78 (dd, *J* = 15.8 Hz, 9.6 Hz, H-3α, CH_2_, 1H), 3.69 (s, OCH_3_, 3H), 3.01 (d, *J* = 15.6 Hz, H-3β, CH_2_, 1H); ^13^C-NMR (100 MHz; CDCl_3_) *δ*_C_ 159.9 (C-3'), 158.3 (C-1"), 148.0 (C-2"), 145.5 (C-1'), 144.3 (C-5"), 143.6 (C-9), 130.0 (C-4, C-5'), 127.8 (C-7), 125.1 (C-5), 124.8 (C-6), 117.9 (C-8), 117.3 (C-4'), 117.1 (C-3"), 112.4 (C-6'), 111.7 (C-4"), 111.0 (C-2'), 63.3 (C-2), 55.2 (OCH_3_), 39.3 (C-3) ; HRMS (+ESI) [M+H]^+^: 320.1286, C_20_H_18_NO_3_ requires 320.1281.

#### 3.2.8. (2-(3,4-Dimethoxyphenyl)indolin-1-yl)(furan-2-yl)methanone (**20a**)

Yield 0.15 g (46%); *ν*_max_/cm^−1^ (NaCl): 3008, 1634, 1479, 1397, 1257, 1140, 1026, 754; λ_max_ (MeOH)/nm: 207, 283, 296; ^1^H-NMR (400 MHz; CDCl_3_) *δ*_H_ 8.21 (d, *J* = 5.5 Hz, H-8, 1H), 7.41 (dd, *J* = 1.8 Hz, 0.92 Hz, H-5", 1H), 7.26 (t, *J* = 7.8 Hz, H-7, 1H), 7.16 (d, *J* = 7.8 Hz, H-5, 1H), 7.07 (td, *J* = 7.6 Hz, 0.92Hz, H-6, 1H), 6.89 (d, *J* = 3.6 Hz, H-3", 1H), 6.63-6.68 (m, H-2', H-5', H-6', 3H), 6.36 (dd, *J* = 3.4Hz, 1.4 Hz, H-4", 1H), 6.02 (dd, *J* = 9.6 Hz, 1.8 Hz, H-2, CH, 1H), 3.76-3.82 (m, H-3α, OCH_3_, 4H), 3.73 (s, OCH_3_, 3H), 3.01 (dd, *J* = 16.0 Hz, 1.84Hz, H-3β, CH_2_, 1H); ^13^C-NMR (100 MHz; CDCl_3_) *δ*_C_ 158.5 (C-1"), 149.1 (C-3'), 148.3 (C-4'), 148.1 (C-2"), 144.1 (C-5"), 143.5 (C-9), 136.4 (C-1'), 130.1 (C-4), 127.7 (C-7), 125.1 (C-5), 124.7 (C-6), 117.7 (C-8), 117.1 (C-5'), 117.0 (C-3"), 111.7 (C-4"), 111.3 (C-6'), 108.2 (C-2'), 63.1 (C-2), 55.9 (2 x OCH_3_), 39.4 (C-3); HRMS (+ESI) [M+H]^+^: 350.1404, C_21_H_20_NO_4_ requires 350.1387; [M+Na]^+^: 372.1215, C_21_H_19_NNaO_4_ requires 372.1206.

#### 3.2.9. (2,2'- bis(3,4-Dimethoxyphenyl)-3,3'-biindoline-1,1'-diyl)-bis(furan-2-ylmethanone) (**(±)20b**)

Yield 0.07 g (10%); *ν*_max_/cm^−1^ (NaCl): 2934, 1636, 1516, 1476, 1394, 1254, 1141, 1025, 754; λ_max_ (MeOH)/nm: 207, 283; ^1^H-NMR (400 MHz; CDCl_3_) *δ*_H_ 8.59 (d, *J* = 7.8 Hz, H-8, H-8*, 2H), 7.48 (td, *J* = 7.8 Hz, 1.4 Hz, H-7, H-7*, 2H), 7.36 (d, *J* = 7.3 Hz, H-5, H-5*, 2H), 7.23-7.27 (m, H-6, H-6*, H-5", H-5"*, 4H), 6.67 (d, *J* = 3.6 Hz, H-3", H-3"*, 2H), 6.54 (d, *J* = 8.2 Hz, H-5', H-5'*, 2H), 6.28 (d, *J* = 1.8 Hz, H-6', H-6'*, 2H), 6.21 (dd, *J* = 3.4 Hz, 1.8 Hz, H-4", H-4"*, 2H), 5.83 (d, *J* = 1.8 Hz, H-2', H-2'*, 2H), 5.78 (s, H-2, H-2*, CH, 2H), 3.70 (s, 2 x OCH_3_, 6H), 3.60 (s, H-3, H-3*, 2H), 3.57 (s, 2 x OCH_3_, 6H); ^13^C-NMR (100 MHz; CDCl_3_) *δ*_C_ 158.4 (C-1", C-1"*), 149.1 (C-3', C-3'*), 148.1 (C-4', C-4'*), 147.4 (C-2", C-2"*), 144.9 (C-9, C-9*), 144.6 (C-5", C-5"*), 135.6 (C-1', C-1'*), 130.8 (C-4, C-4*), 129.2 (C-7, C-7*), 125.4 (C-5, C-5*), 125.0 (C-6, C-6*), 118.2 (C-8, C-8*), 117.3 (C-3", C-3"*), 115.9 (C-6', C-6'*), 111.5 (C-4", C-4"*), 111.1 (C-5', C-5'*), 107.6 (C-2', C-2'*), 63.6 (C-2, C-2*), 57.3 (C-3, C-3*), 55.81 (2 x OCH_3_), 55.76 (2 x OCH_3_); HRMS (+ESI) [M+H]^+^: 697.2542, C_42_H_37_N_2_O_8_ requires 697.2544.

#### 3.2.10. Furan-2-yl(2-(4-methoxyphenyl)indolin-1-yl)methanone (**21a**)

Yield 0.10 g (21%); *ν*_max_/cm^−1^ (NaCl): 3005, 1635, 1513, 1478, 1397, 1249, 1179, 1034, 834, 755; λ_max_ (MeOH)/nm: 203, 226, 283, 297; ^1^H-NMR (400 MHz; CDCl_3_) *δ*_H_ 8.25 (d, *J* = 4.6 Hz, H-8, 1H), 7.40 (dd, *J* = 1.8 Hz, 0.92 Hz, H-5", 1H), 7.27 (t, *J* = 7.8 Hz, H-7, 1H), 7.16 (d, *J* = 7.8 Hz, H-5, 1H), 7.04-7.09 (m, H-6, H-2', H-6', 3H), 6.90 (d, *J* = 4.1 Hz, H-3", 1H), 6.72-6.75 (m, H-3', H-5', 2H), 6.35 (dd, *J* = 3.7Hz, 1.8 Hz, H-4", 1H), 6.05 (dd, *J* = 9.6 Hz, 1.8 Hz, H-2, CH, 1H), 3.76 (dd, *J* = 16.0 Hz, 10.1Hz, H-3α, CH_2_, 1H), 3.70 (s, OCH_3_, 3H), 2.98 (dd, *J* = 16.0 Hz, 1.4Hz, H-3β, CH_2_, 1H); ^13^C-NMR (100 MHz; CDCl_3_) *δ*_C_ 158.8 (C-4'), 158.4 (C-1"), 148.1 (C-2"), 144.2 (C-5"), 143.6 (C-9), 136.0 (C-1'), 130.1 (C-4), 127.7 (C-7), 126.1 (C-2', C-6'), 125.1 (C-5), 124.7 (C-6), 117.8 (C-8), 117.0 (C-3"), 114.2 (C-3', C-5'), 111.7 (C-4"), 62.8 (C-2), 55.3 (OCH_3_), 39.4 (C-3); HRMS (+ESI) [M+H]^+^: 320.1294, C_20_H_18_NO_3_ requires 320.1281; [M+Na]^+^: 342.1105, C_20_H_17_NNaO_3_ requires 342.1101.

#### 3.2.11. (2,2'- bis(4-methoxyphenyl)- 3,3'- biindoline-1,1'-diyl) bis(furan-2-ylmethanone) (**(±)21b**)

Yield 0.11 g (12%); *ν*_max_/cm^−1^ (NaCl): 3008, 1636, 1512, 1477, 1395, 1249, 1177, 1034, 755; λ_max_ (MeOH)/nm: 205, 226, 284; ^1^H-NMR (400 MHz; CDCl_3_) *δ*_H_ 8.59 (d, *J* = 8.2 Hz, H-8, H-8*, 2H), 7.49 (td, *J* = 7.8 Hz, 1.4 Hz, H-7, H-7*, 2H), 7.31 (d, *J* = 6.4 Hz, H-5, H-5*, 2H), 7.24-7.27 (m, H-6, H-6*, H-5", H-5"*, 4H), 6.69 (d, *J* = 3.6 Hz, H-3", H-3"*, 2H), 6.55-6.60 (m, H-3', H-3'*, H-5', H-5'*, 4H), 6.44-6.47 (m, H-2', H-2'*, H-6', H-6'*, 4H), 6.20 (dd, *J* = 3.4 Hz, 1.8 Hz, H-4", H-4"*, 2H), 5.81 (s, H-2, H-2*, CH, 2H), 3.63 (s, 2 x OCH_3_, 6H), 3.55 (s, H-3, H-3*, 2H); ^13^C-NMR (100 MHz; CDCl_3_) *δ*_C_ 158.7 (C-4', C-4'*), 158.3 (C-1", C-1"*), 147.5 (C-2", C-2"*), 144.8 (C-9, C-9*), 144.6 (C-5", C-5"*), 135.3 (C-1', C-1'*), 130.7 (C-4, C-4*), 129.3 (C-7, C-7*), 125.6 (C-2', C-2'*, C-6', C-6'*), 125.4 (C-5, C-5*), 125.1 (C-6, C-6*), 118.1 (C-8, C-8*), 117.3 (C-3", C-3"*), 114.2 (C-3', C-3'*, C-5', C-5'*), 111.4 (C-4", C-4"*), 63.5 (C-2, C-2*), 57.2 (C-3, C-3*), 55.2 (2 x OCH_3_); HRMS (+ESI) [M+H]^+^: 637.2341, C_40_H_33_N_2_O_6_ requires 637.2333.

## 4. Conclusions

Although previous studies by our group have demonstrated the synthetic utility of benzophenone (an organocatalyst) as an effective additive for dramatically improving the yield of indolines (and suppressing bisindoline formation), the present study demonstrated that even in the absence of benzophenone, the amide structure can also under certain conditions discriminate between the indoline and bisindoline forms (enhancing the former and suppressing the latter). Stilbene **5** (with an *n*-butyramide moiety) will, on exposure to FeCl_3_, give the corresponding indoline **14a** in 63% yield, compared to stilbene **6** (with an isobutyramide moiety) which yields indoline **15** in 29%. Previously undiscovered steric, conformational and stereoelectronic effects have been considered in this study involving eight stilbenes.

We would like to draw the reader’s attention to some intriguing electronic effects. For example, stilbene **9** with an *ortho*-methoxy substituent dramatically shifts the electron density in the olefinic bond (see Table 1). The C(7) olefinic proton is now more deshielded than the C(8) proton. This in turn changes the position of the positive charge and unpaired electron (in the radical cation) compared to stilbenes **11** and **12** with predictable consequences for the cyclization/dimerization. These transformations exploit FeCl_3_ (an environmentally friendly oxidant) as the single electron transfer reagent [19,20,21]. Further developments will be reported in due course.

## Supplementary Materials

Supplementary File 1
