# Supplementary material for: Cyclization vs. Cyclization/Dimerization in o-Amidostilbene Radical Cation Cascade Reactions: The Amide Question
Source: Molecules. 2011 Aug 25;16(9):7267–87. doi: 10.3390/molecules16097267 (PMC6264379; doi:10.3390/molecules16097267)
Supplement: Supplementary File 1 [file molecules-16-07267-s001.zip › supplementry/Crystallographic data of 4b.pdf]

X-ray Crystal Data for Compound 4b. X-ray diffraction analysis was carried out on a Bruker SMART APEX II CCD area detector system equipped with a graphite monochromator and a Mo Kα fine-focus sealed tube (λ=0.71073 Å), at 100K. The structure was solved by direct methods (SHELXS-97) and refined with full-matrix least-squares on F^2 (SHELXL-97). All non-hydrogen atoms were refined anisotropically, and all hydrogen atoms were placed in idealized positions and refined as riding atoms with the relative isotropic parameters.

Crystallographic data of **Compound 4b**: Colorless block crystals from EtOH, $C_{36}H_{36}N_2O_6.H_2O$, Mr = 610.68, monoclinic, space group $P2_1/c$, a = 16.4733(3) Å, b = 18.0651(3) Å, c = 31.8360(6) Å, $\alpha = \gamma = 90^o$, $\beta = 91.2600(10)^o$, V = 9471.8(3) Å^3, T = 100 K, Z = 12, D_{calcd} = 1.285 gcm^{-3}, crystal size 0.20 x 0.35 x 0.49 mm^3, F(000) = 3888. The final R_1 value is 0.0534 (wR_2 = 0.1141) for 21783 reflections [$I>2\sigma(I)$].

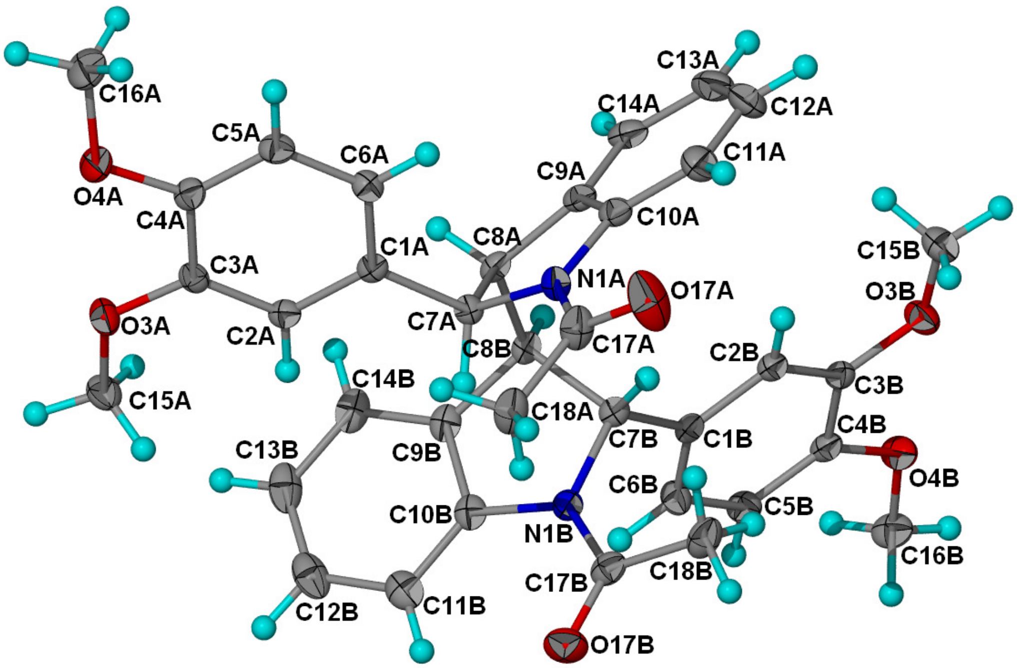

Figure 1. X-ray crystal structure of **Compound 4b**. Thermal ellipsoids are shown at the 50% probability level.

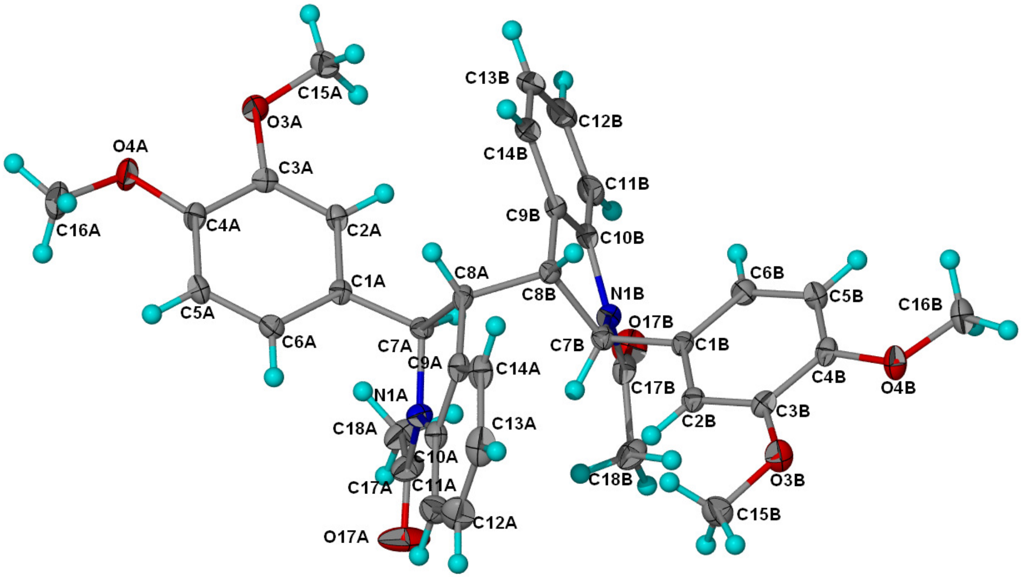

Figure 2. X-ray crystal structure of **Compound 4b**. Thermal ellipsoids are shown at the 50% probability level.
