# Supplementary material for: Cyclization vs. Cyclization/Dimerization in o-Amidostilbene Radical Cation Cascade Reactions: The Amide Question
Source: Molecules. 2011 Aug 25;16(9):7267–87. doi: 10.3390/molecules16097267 (PMC6264379; doi:10.3390/molecules16097267)
Supplement: Supplementary File 1 [file molecules-16-07267-s001.zip › supplementry/NMRSuppData.pdf]

Electronic Supplementary Information
Cyclization vs. Cyclization / Dimerization in *o*-amidostilbene Radical Cation Cascade Reactions : The Amide Question (Spectral Data)

Chin Hui Kee, Azhar Ariffin, Khalijah Awang, Ibrahim Noorbatcha, Koichi Takeya, Hiroshi Morita, Chuan Gee Lim and Noel Francis Thomas*
noelfthomas@um.edu.my

Contents

List of Figures

S1 ^{1}H spectrum ($CDCl_3$, 400MHz) of **14a**. 3
S2 ^{13}C spectrum ($CDCl_3$, 100MHz) of **14a**. 4
S3 HSQC spectrum ($CDCl_3$, 400MHz) of **14a**. 5
S4 HMBC spectrum ($CDCl_3$, 400MHz) of **14a**. 6
S5 ^{1}H spectrum ($CDCl_3$, 400MHz) of **(±)14b**. 7
S6 ^{13}C spectrum ($CDCl_3$, 100MHz) of **(±)14b**. 8
S7 HSQC spectrum ($CDCl_3$, 400MHz) of **(±)14b**. 9
S8 HMBC spectrum ($CDCl_3$, 400MHz) of **(±)14b**. 10
S9 H2BC spectrum ($CDCl_3$, 400MHz) of **(±)14b**. 11
S10 ^{1}H spectrum ($CDCl_3$, 400MHz) of **15**. 12
S11 ^{13}C spectrum ($CDCl_3$, 100MHz) of **15**. 13
S12 HSQC spectrum ($CDCl_3$, 400MHz) of **15**. 14
S13 HMBC spectrum ($CDCl_3$, 400MHz) of **15**. 15
S14 ^{1}H spectrum ($CDCl_3$, 400MHz) of **16**. 16
S15 ^{13}C spectrum ($CDCl_3$, 100MHz) of **16**. 17
S16 HSQC spectrum ($CDCl_3$, 400MHz) of **16**. 18
S17 HMBC spectrum ($CDCl_3$, 400MHz) of **16**. 19
S18 H2BC spectrum ($CDCl_3$, 400MHz) of **16**. 20
S19 ^{1}H spectrum ($CDCl_3$, 400MHz) of **17**. 21
S20 ^{13}C spectrum ($CDCl_3$, 100MHz) of **17**. 22

S21 HMQC spectrum ($CDCl_3$, 400MHz) of **17**. 23

S22 HMBC spectrum ($CDCl_3$, 400MHz) of **17**. 24

S23 ^1H spectrum ($CDCl_3$, 400MHz) of **18**. 25

S24 ^{13}C spectrum ($CDCl_3$, 100MHz) of **18**. 26

S25 HSQC spectrum ($CDCl_3$, 400MHz) of **18**. 27

S26 HMBC spectrum ($CDCl_3$, 400MHz) of **18**. 28

S27 HMBC spectrum ($CDCl_3$, 400MHz) of **18** (expanded). 29

S28 ^1H spectrum ($CDCl_3$, 400MHz) of **19**. 30

S29 ^{13}C spectrum ($CDCl_3$, 100MHz) of **19**. 31

S30 HSQC spectrum ($CDCl_3$, 400MHz) of **19**. 32

S31 HMBC spectrum ($CDCl_3$, 400MHz) of **19**. 33

S32 ^1H spectrum ($CDCl_3$, 400MHz) of **20a**. 34

S33 ^{13}C spectrum ($CDCl_3$, 100MHz) of **20a**. 35

S34 HSQC spectrum ($CDCl_3$, 400MHz) of **20a**. 36

S35 HMBC spectrum ($CDCl_3$, 400MHz) of **20a**. 37

S36 H2BC spectrum ($CDCl_3$, 400MHz) of **20a**. 38

S37 ^1H spectrum ($CDCl_3$, 400MHz) of **(±)20b**. 39

S38 ^{13}C spectrum ($CDCl_3$, 100MHz) of **(±)20b**. 40

S39 HSQC spectrum ($CDCl_3$, 400MHz) of **(±)20b**. 41

S40 HMBC spectrum ($CDCl_3$, 400MHz) of **(±)20b**. 42

S41 H2BC spectrum ($CDCl_3$, 400MHz) of **(±)20b**. 43

S42 ^1H spectrum ($CDCl_3$, 400MHz) of **21a**. 44

S43 ^{13}C spectrum ($CDCl_3$, 100MHz) of **21a**. 45

S44 HSQC spectrum ($CDCl_3$, 400MHz) of **21a**. 46

S45 HMBC spectrum ($CDCl_3$, 400MHz) of **21a**. 47

S46 ^1H spectrum ($CDCl_3$, 400MHz) of **(±)21b**. 48

S47 ^{13}C spectrum ($CDCl_3$, 100MHz) of **(±)21b**. 49

S48 HSQC spectrum ($CDCl_3$, 400MHz) of **(±)21b**. 50

S49 HMBC spectrum ($CDCl_3$, 400MHz) of **(±)21b**. 51

S50 H2BC spectrum ($CDCl_3$, 400MHz) of **(±)21b**. 52

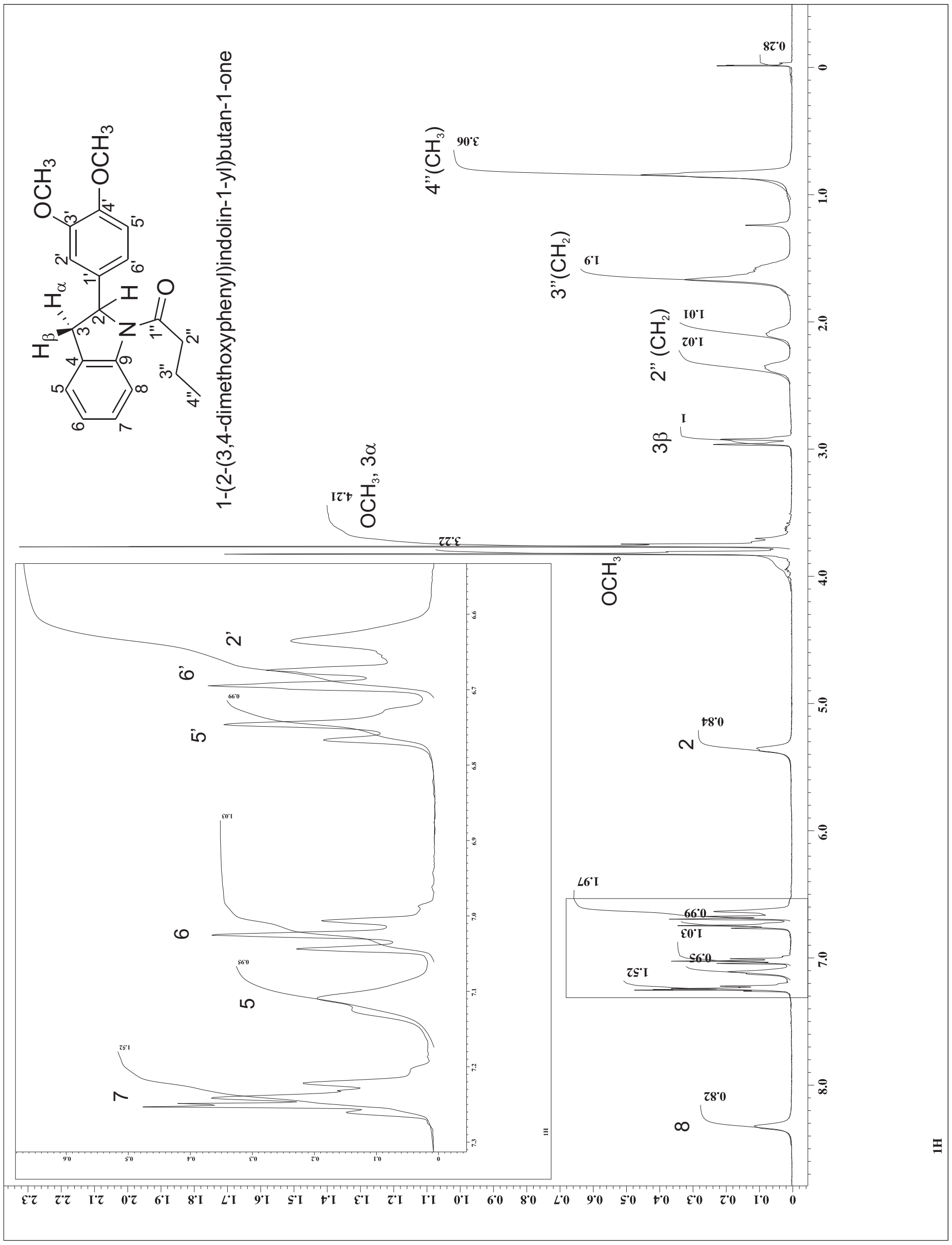

Figure S1: ^{1}H spectrum ($CDCl_3$, 400MHz) of **14a**.

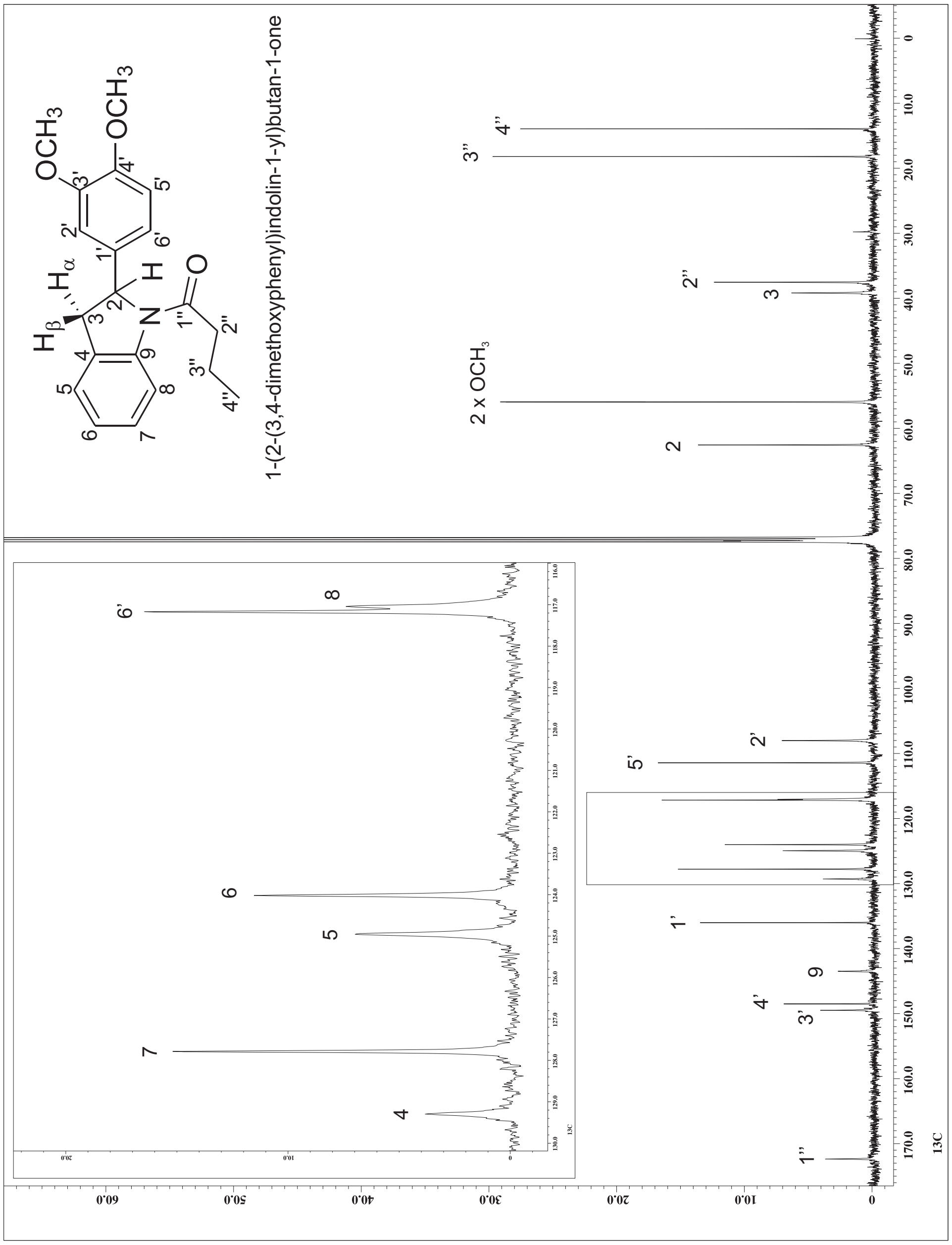

Figure S2: ^{13}C spectrum ($CDCl_3$, 100MHz) of **14a**.

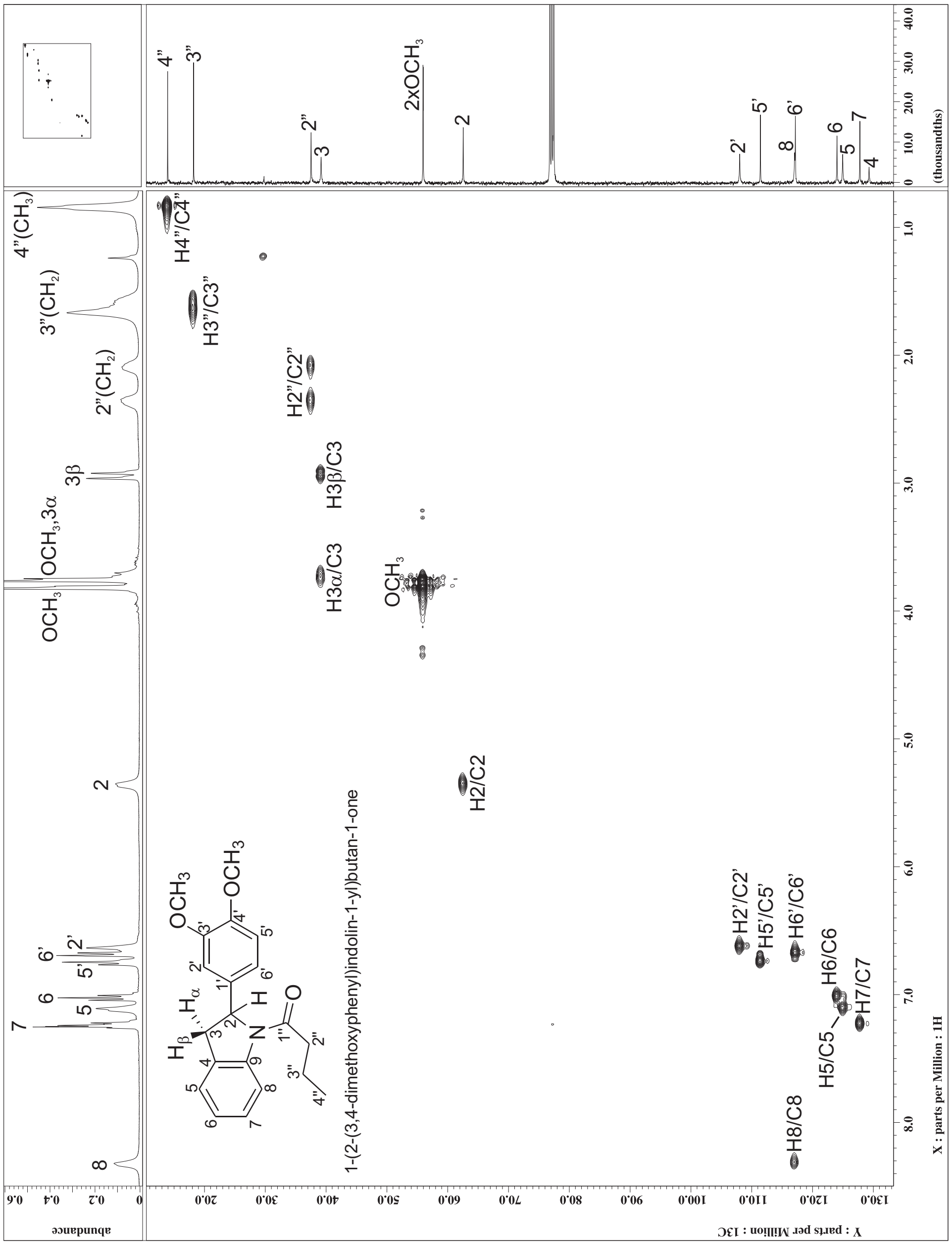

Figure S3: HSQC spectrum ($CDCl_3$, 400MHz) of **14a**.

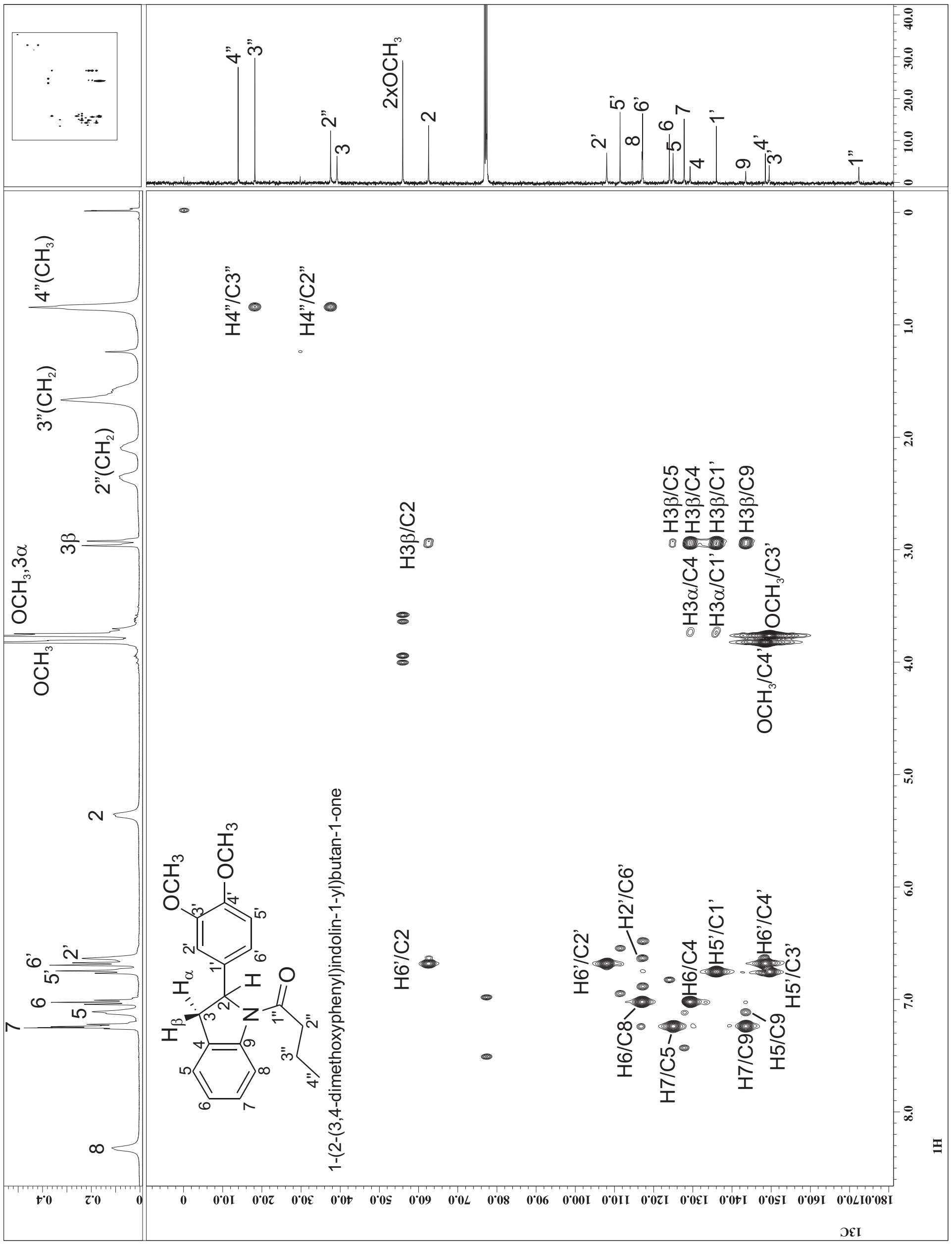

Figure S4: HMBC spectrum ($CDCl_3$, 400MHz) of **14a**.

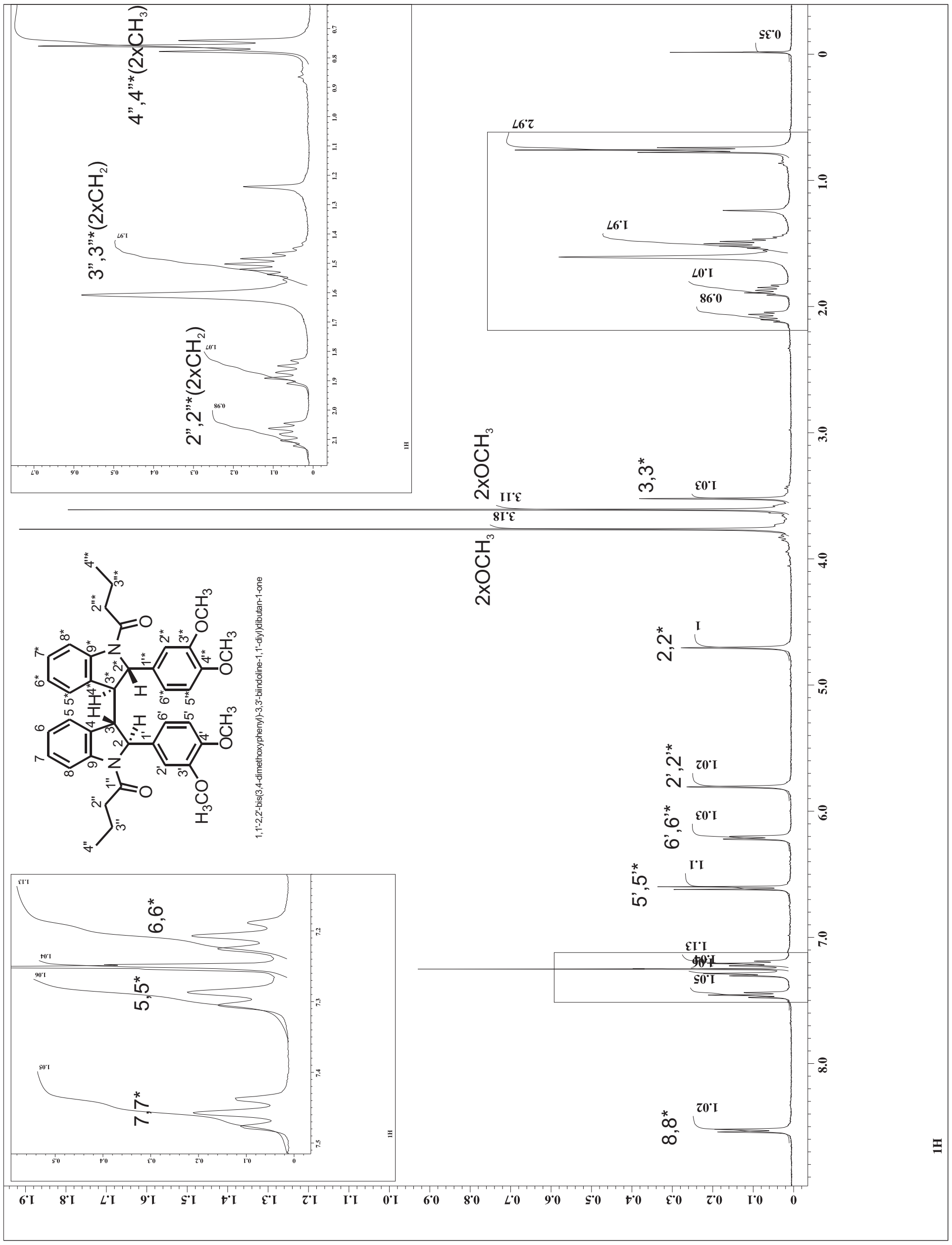

Figure S5: ^{1}H spectrum ($CDCl_3$, 400MHz) of **(±)14b**.

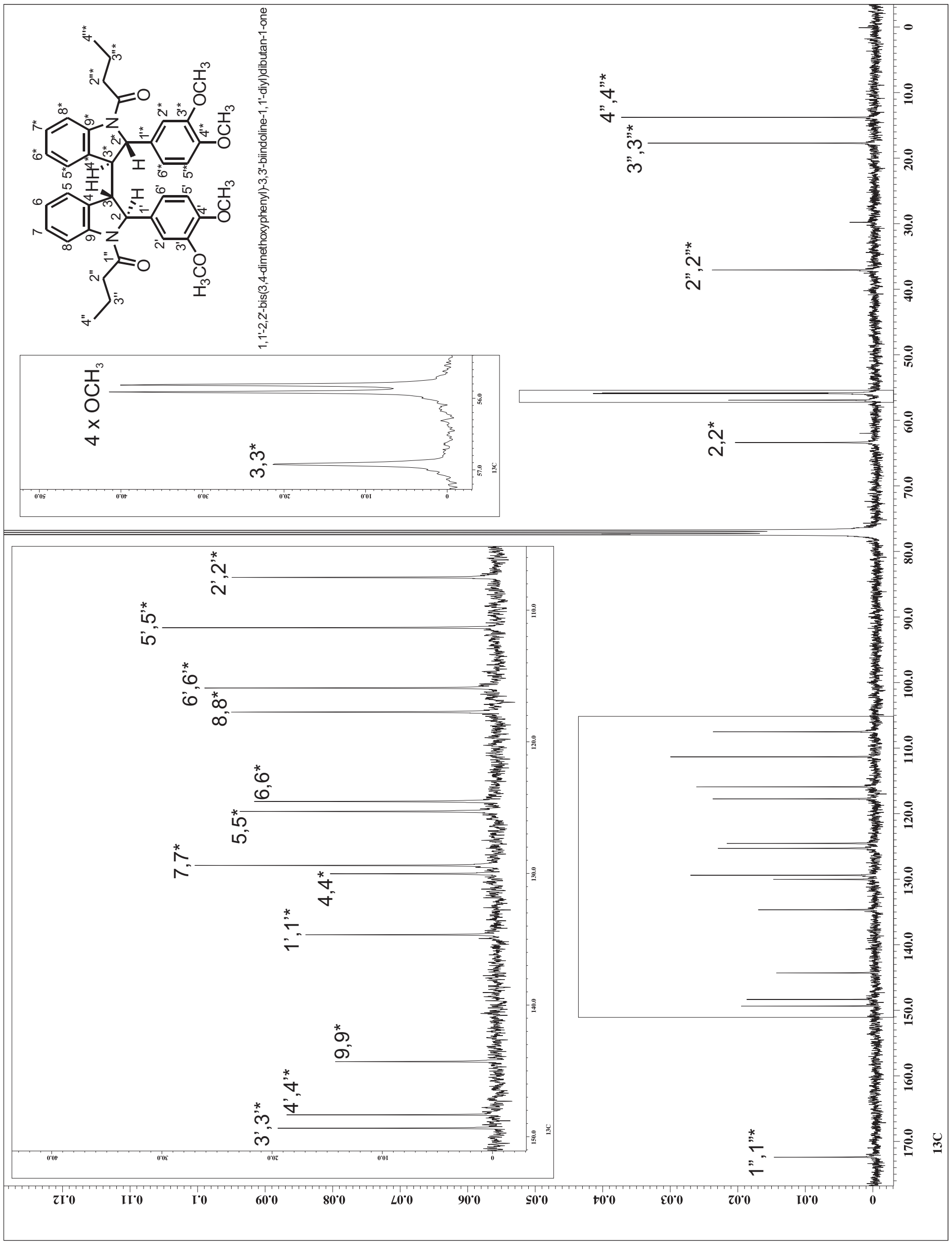

Figure S6: ^{13}C spectrum ($CDCl_3$, 100MHz) of **(±)14b**.

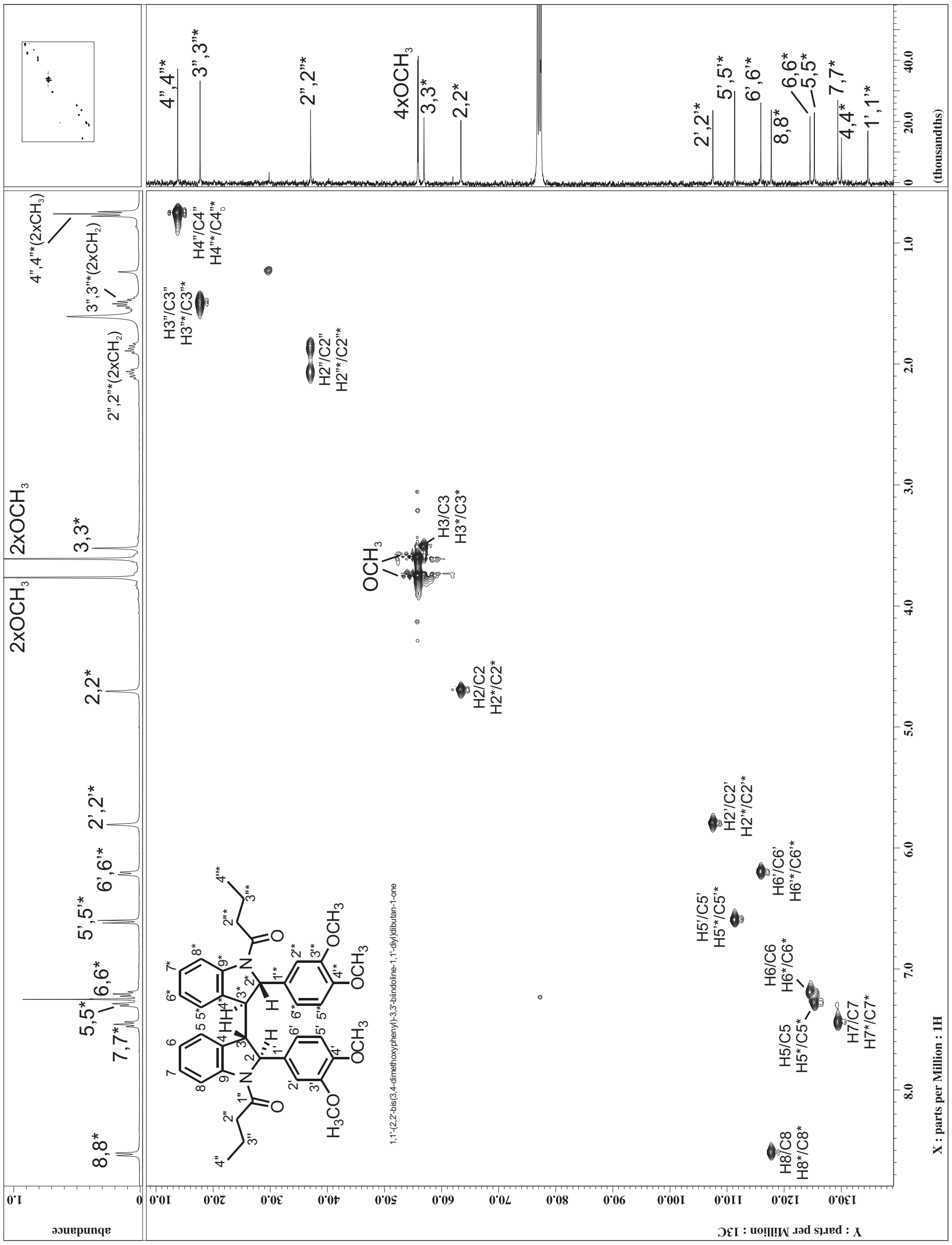

Figure S7: HSQC spectrum ($CDCl_3$, 400MHz) of **(±)14b**.

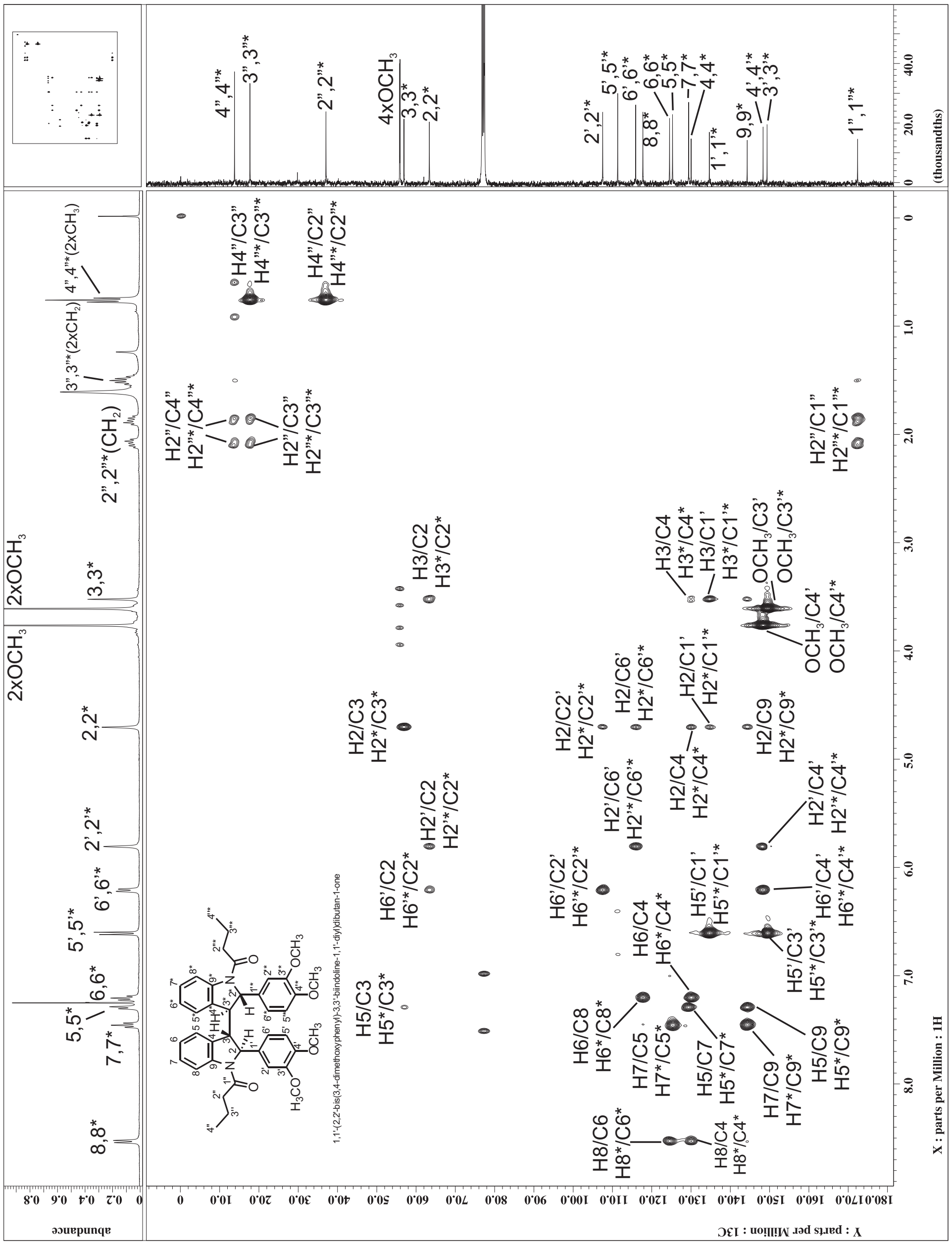

Figure S8: HMBC spectrum ($CDCl_3$, 400MHz) of **(±)14b**.

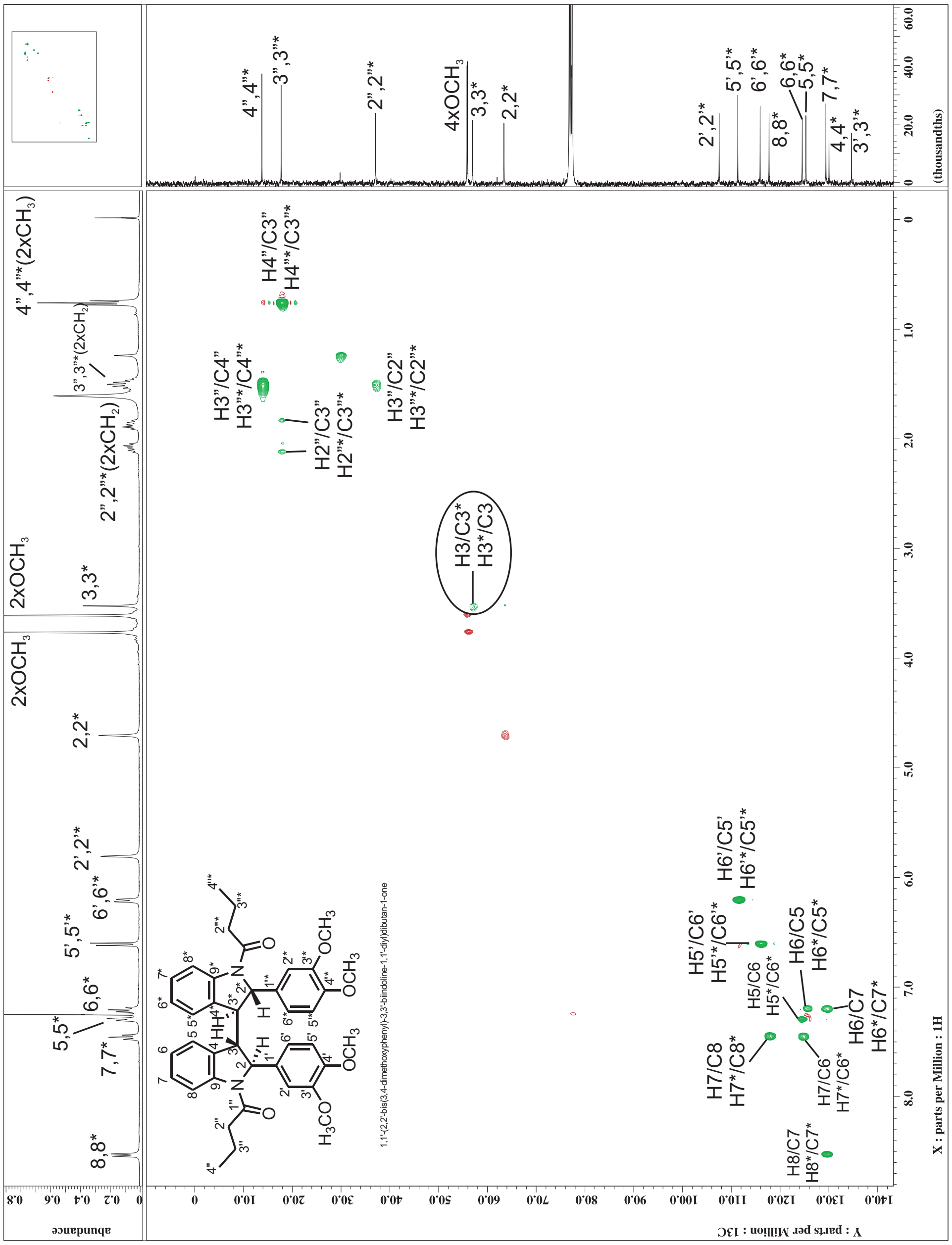

Figure S9: H2BC spectrum ($CDCl_3$, 400MHz) of **(±)14b**.

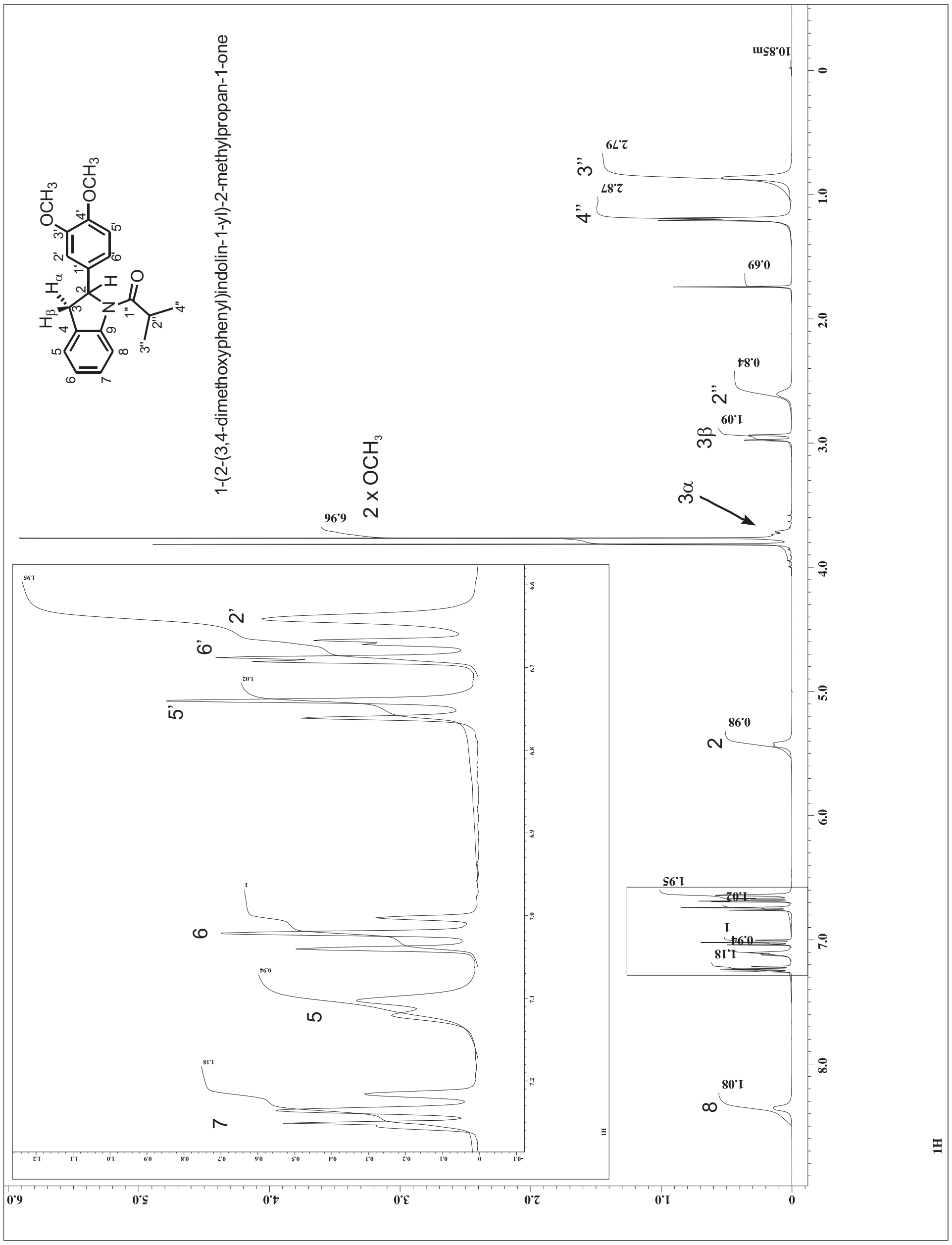

Figure S10: ^{1}H spectrum ($CDCl_3$, 400MHz) of **15**.

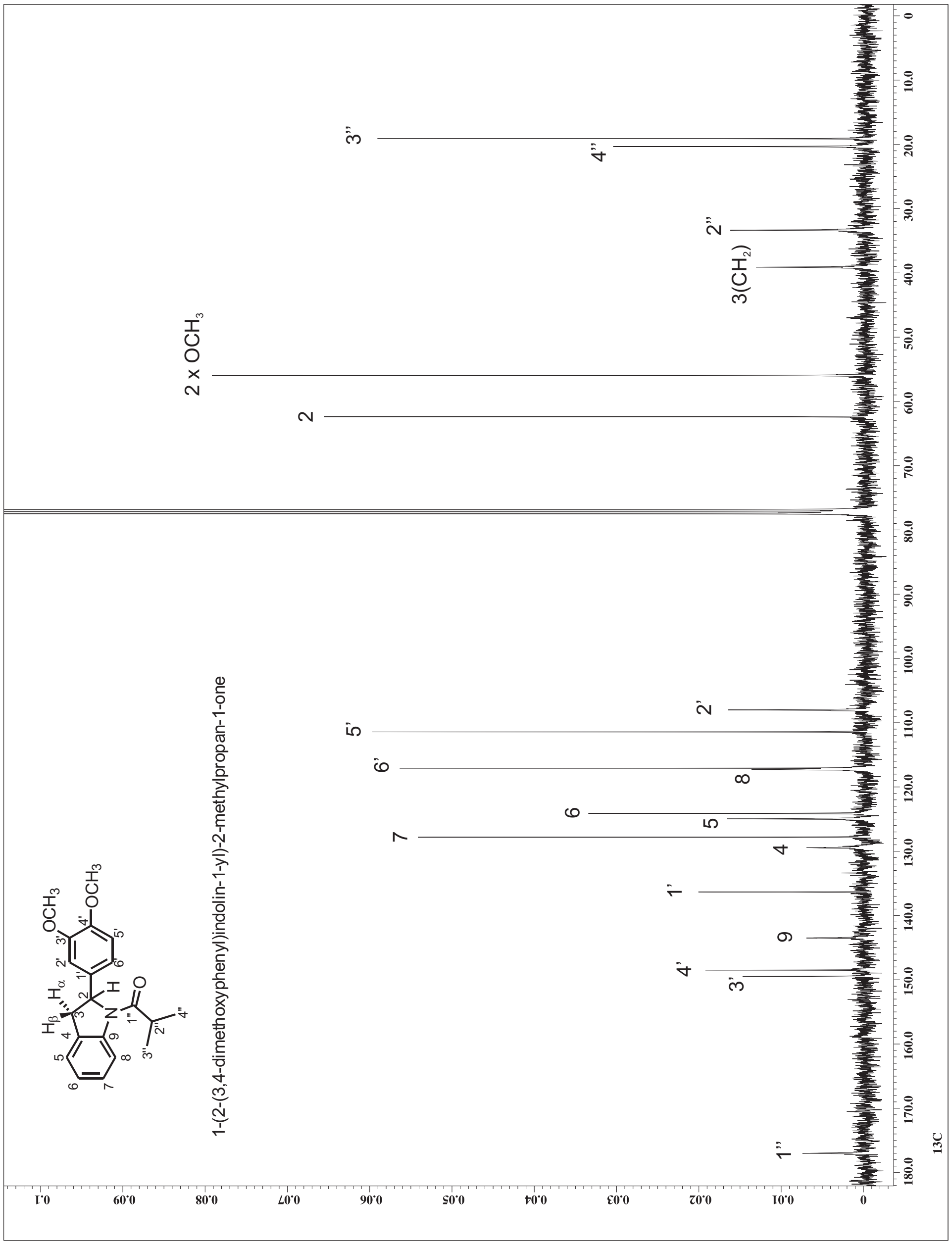

Figure S11: ^{13}C spectrum ($CDCl_3$, 100MHz) of **15**.

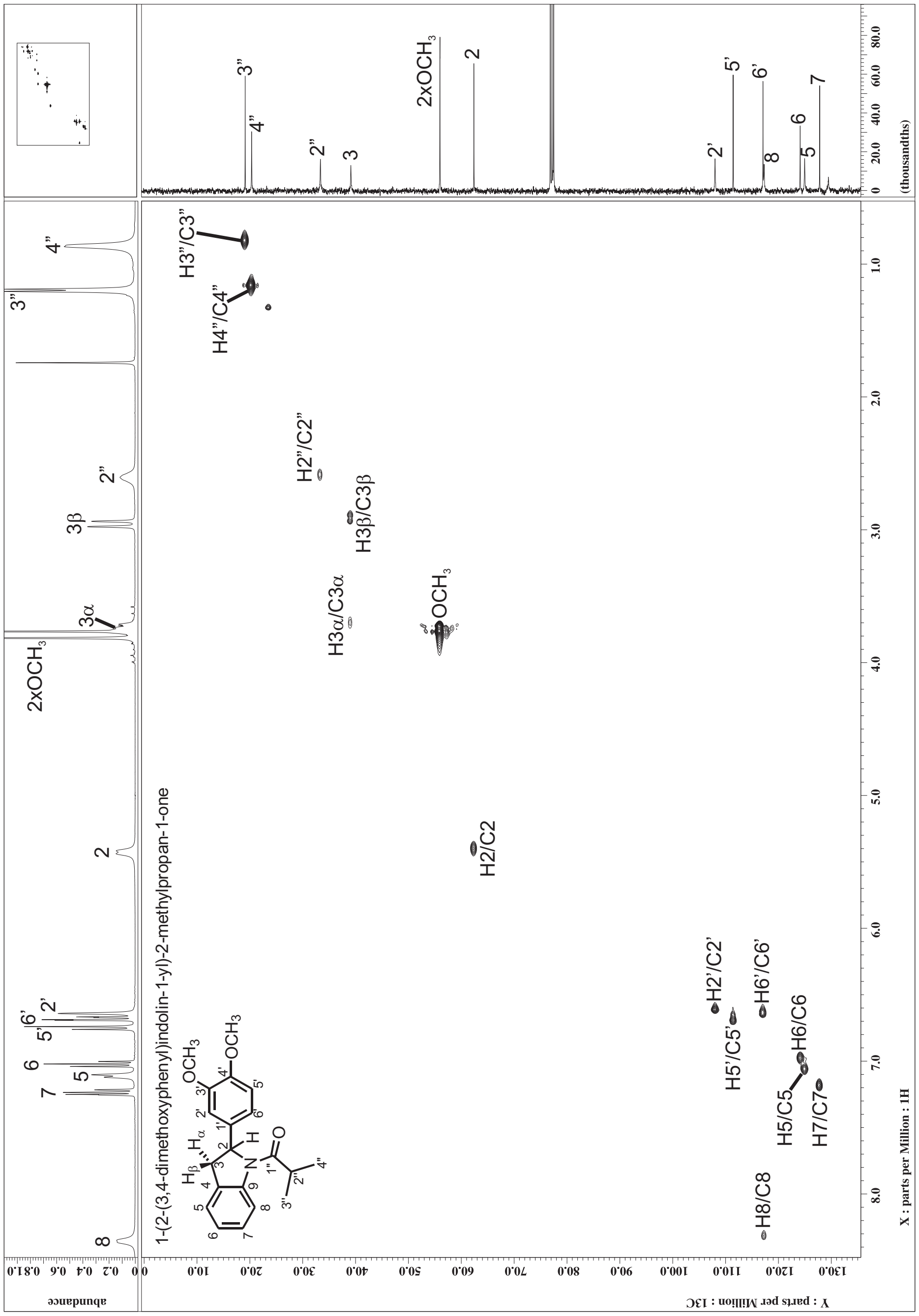

Figure S12: HSQC spectrum ($CDCl_3$, 400MHz) of **15**.

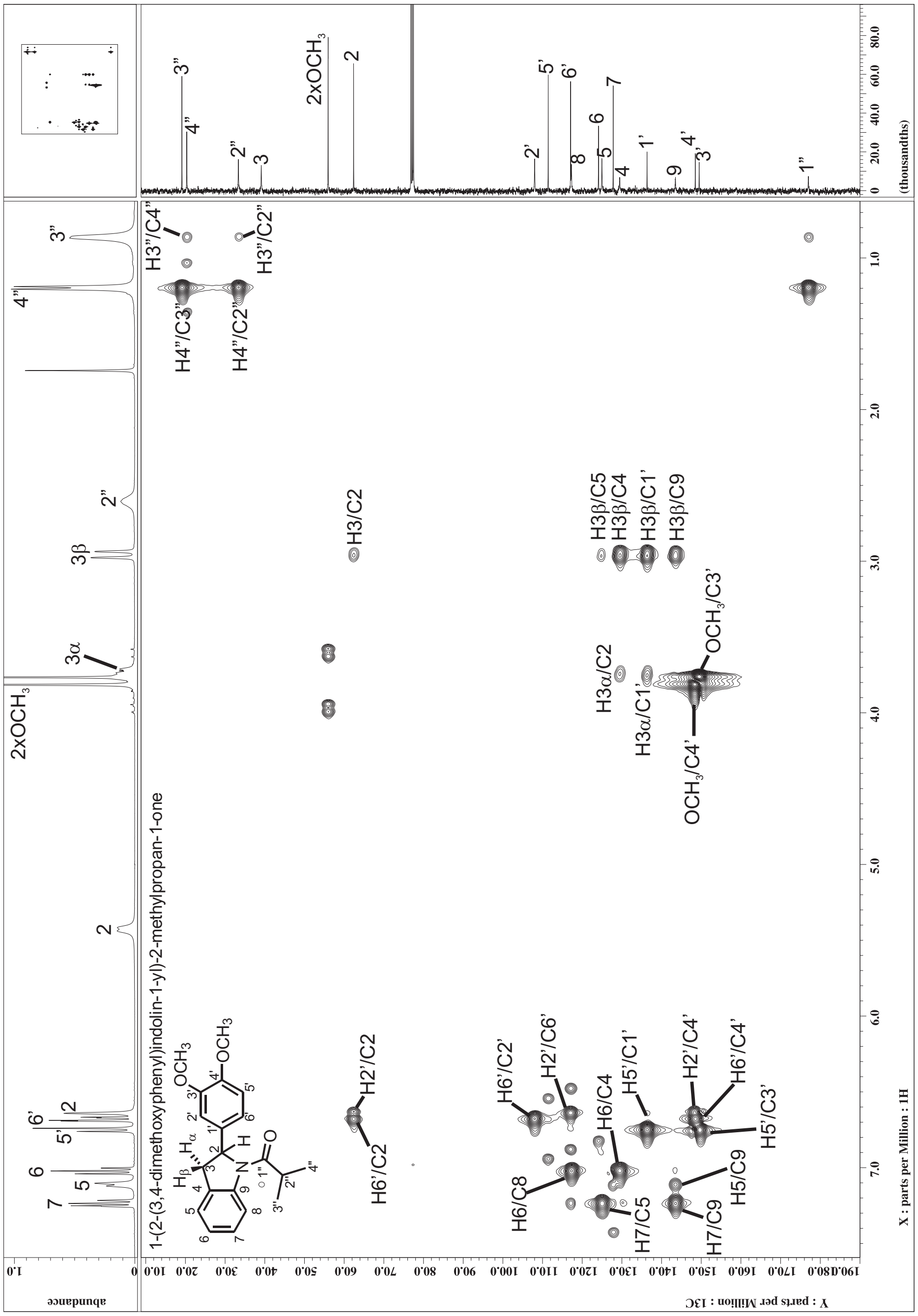

Figure S13: HMBC spectrum ($CDCl_3$, 400MHz) of **15**.

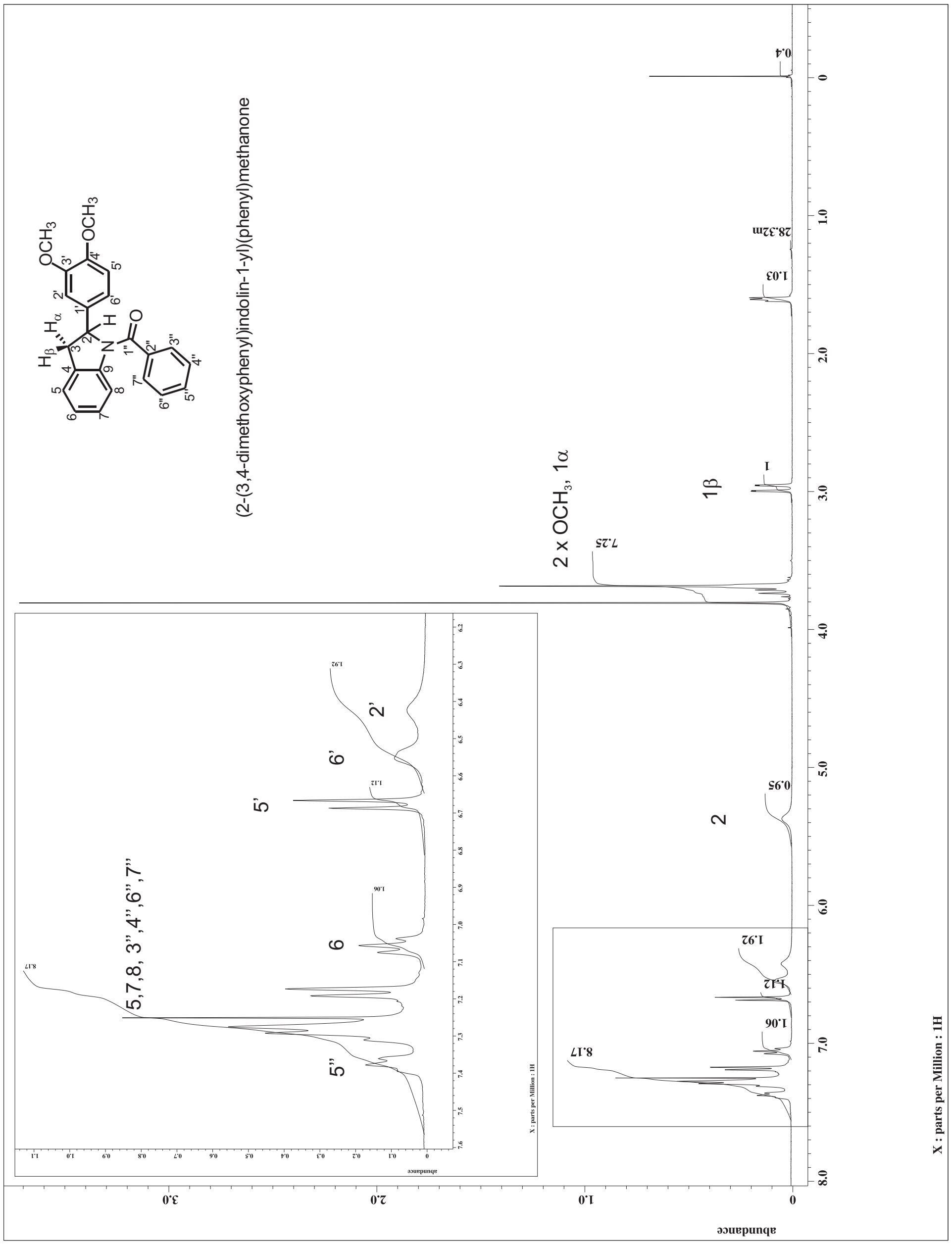

Figure S14: ^{1}H spectrum (CDCl$_3$, 400MHz) of **16**.

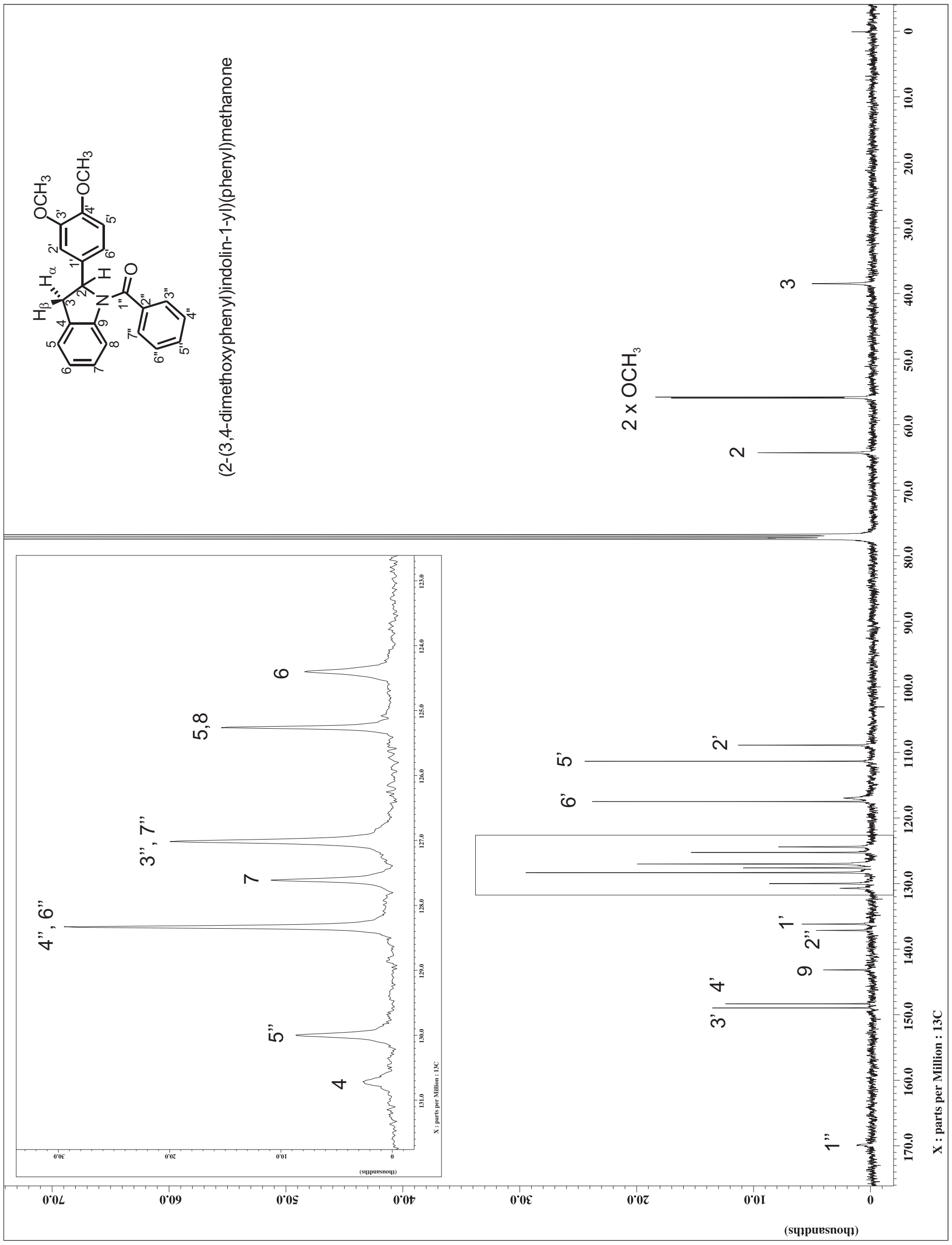

Figure S15: ^{13}C spectrum ($CDCl_3$, 100MHz) of **16**.

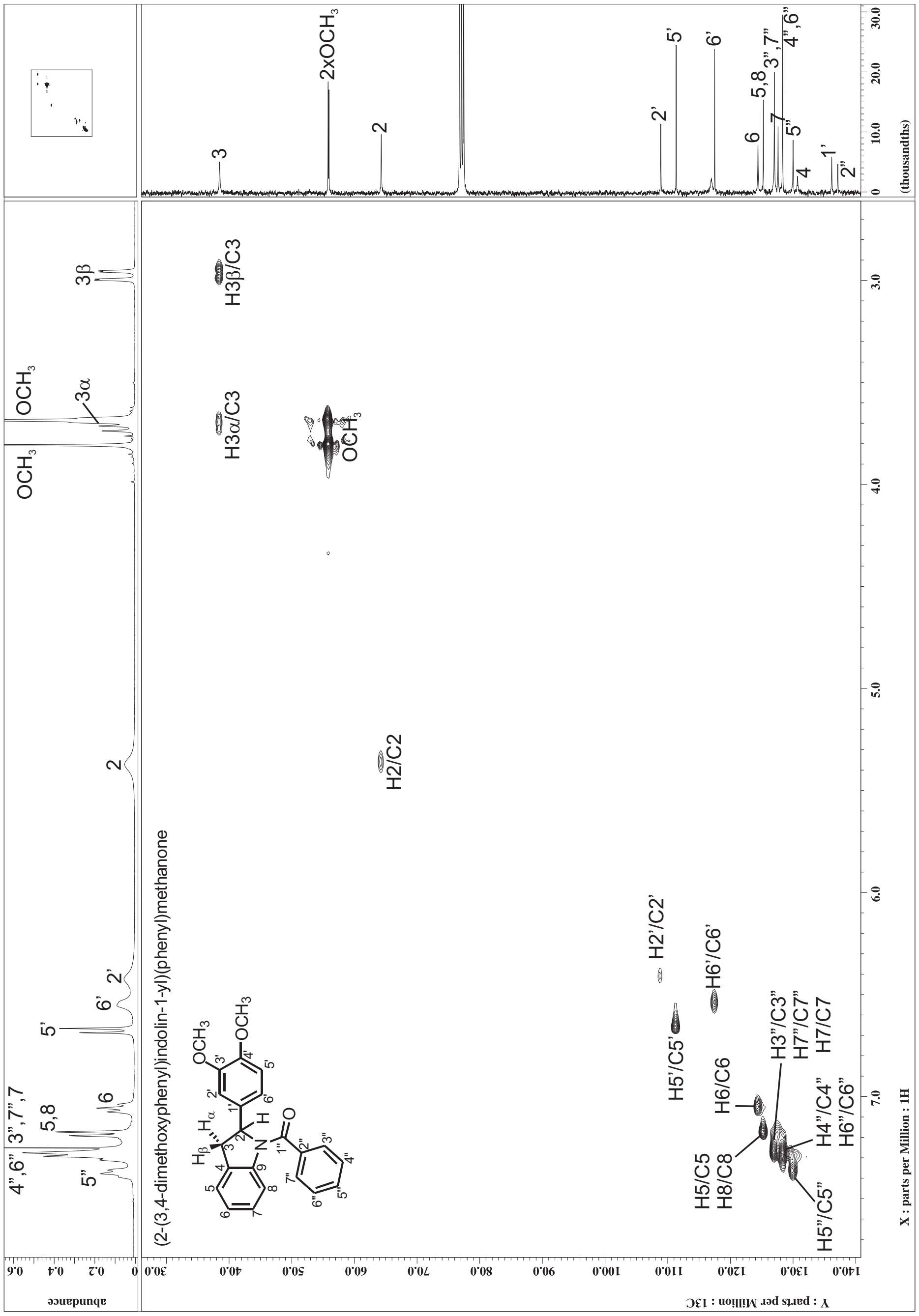

Figure S16: HSQC spectrum ($CDCl_3$, 400MHz) of **16**.

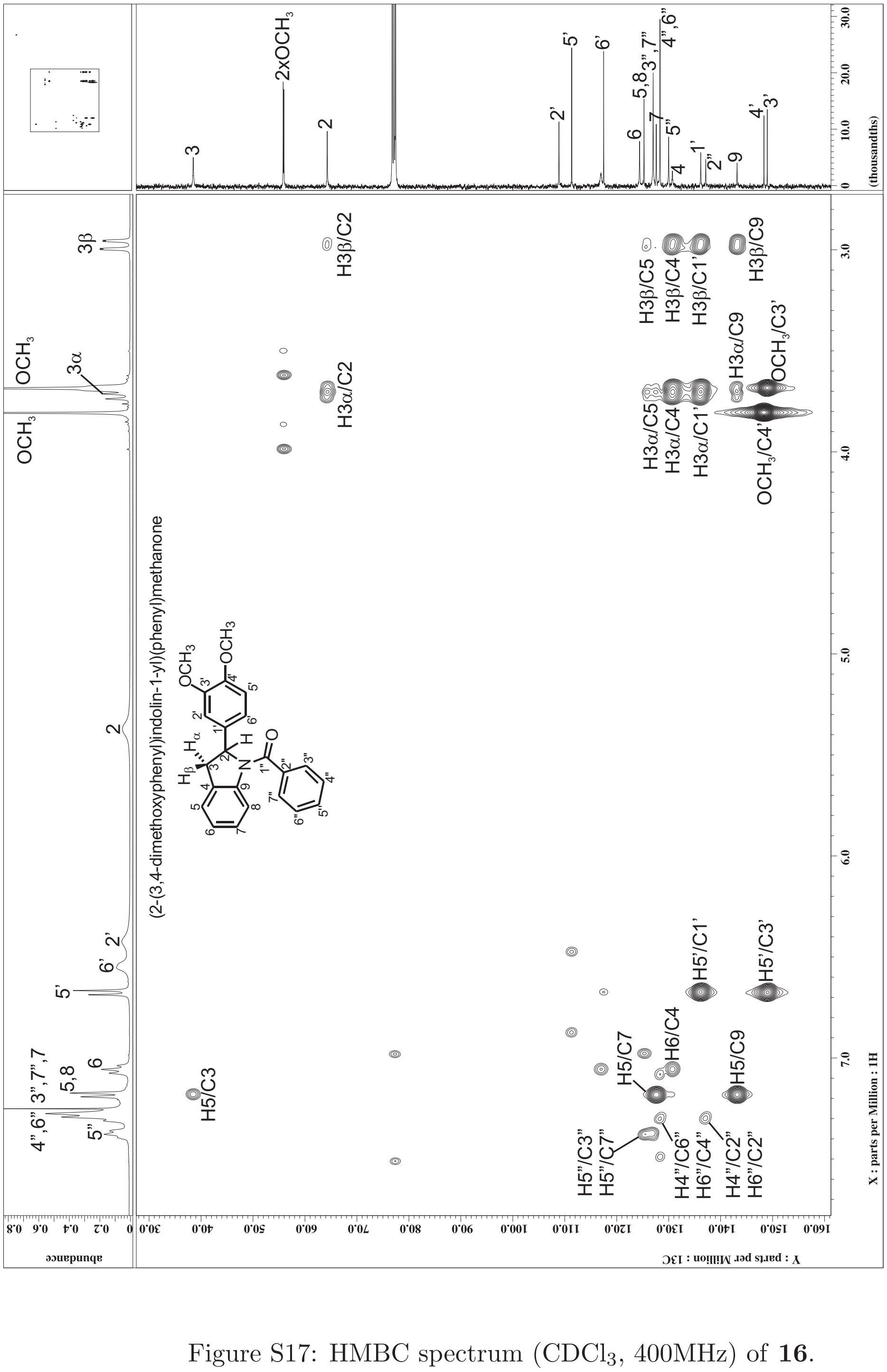

Figure S17: HMBC spectrum ($CDCl_3$, 400MHz) of **16**.

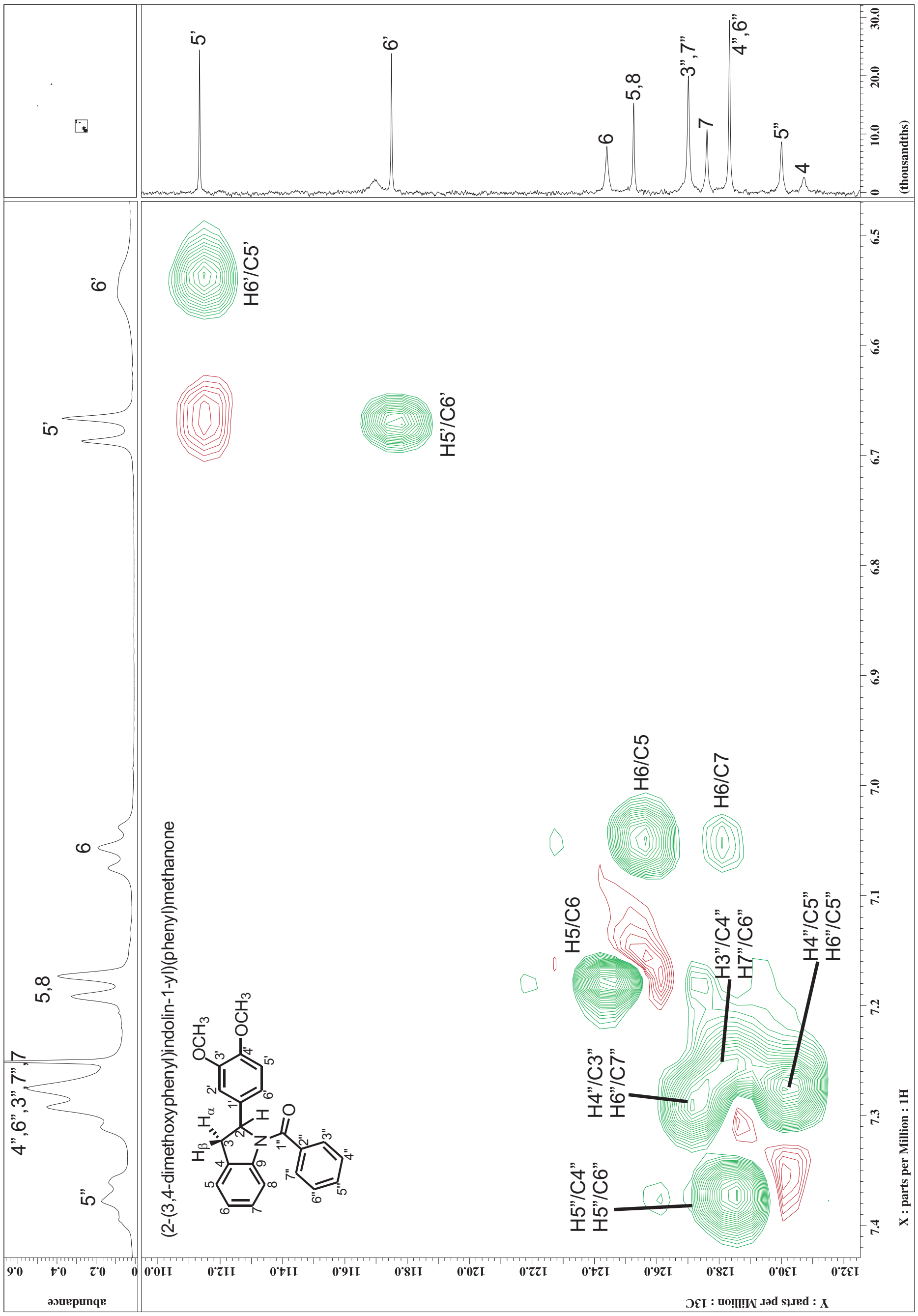

Figure S18: H2BC spectrum ($CDCl_3$, 400MHz) of **16**.

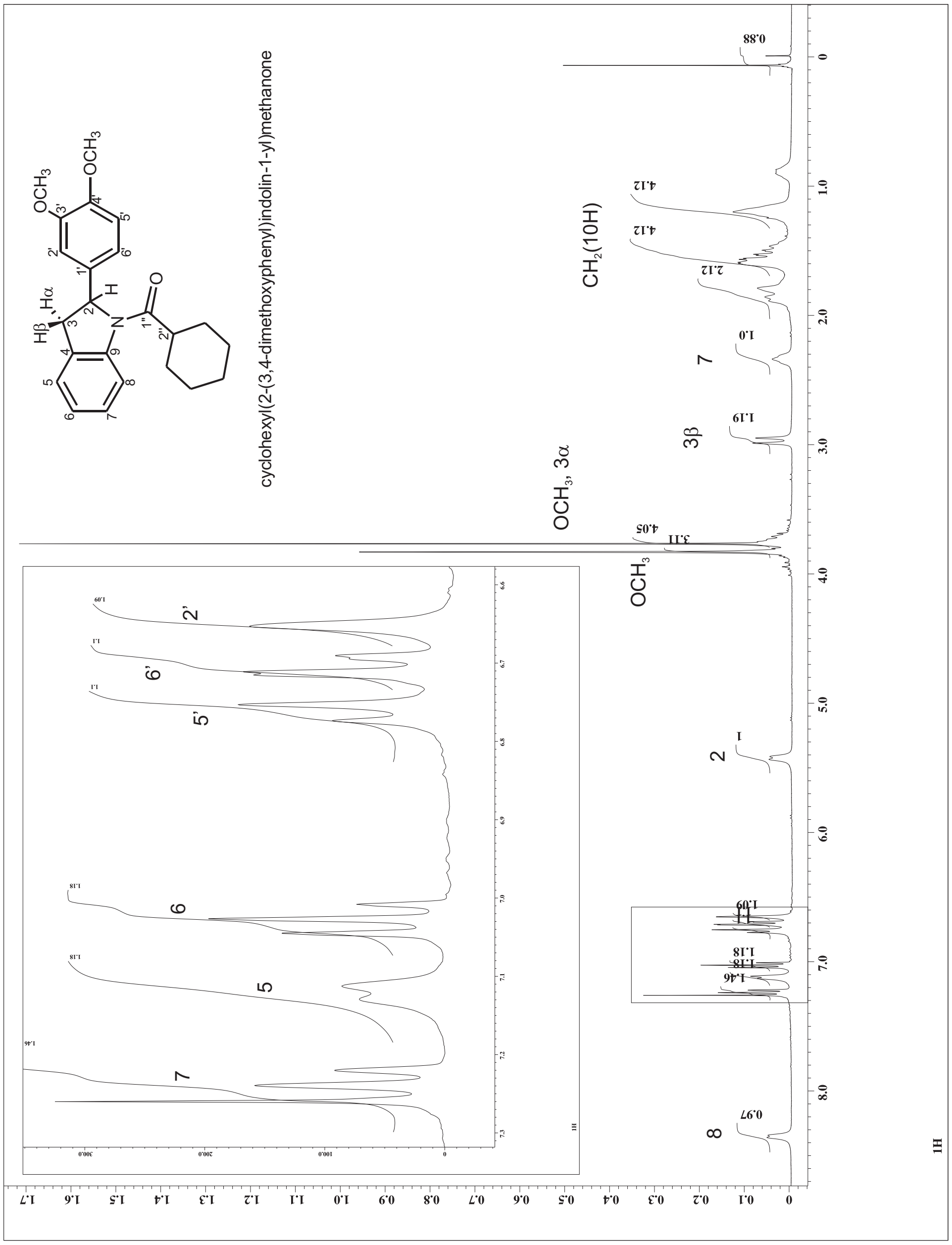

Figure S19: ^{1}H spectrum ($CDCl_3$, 400MHz) of **17**.

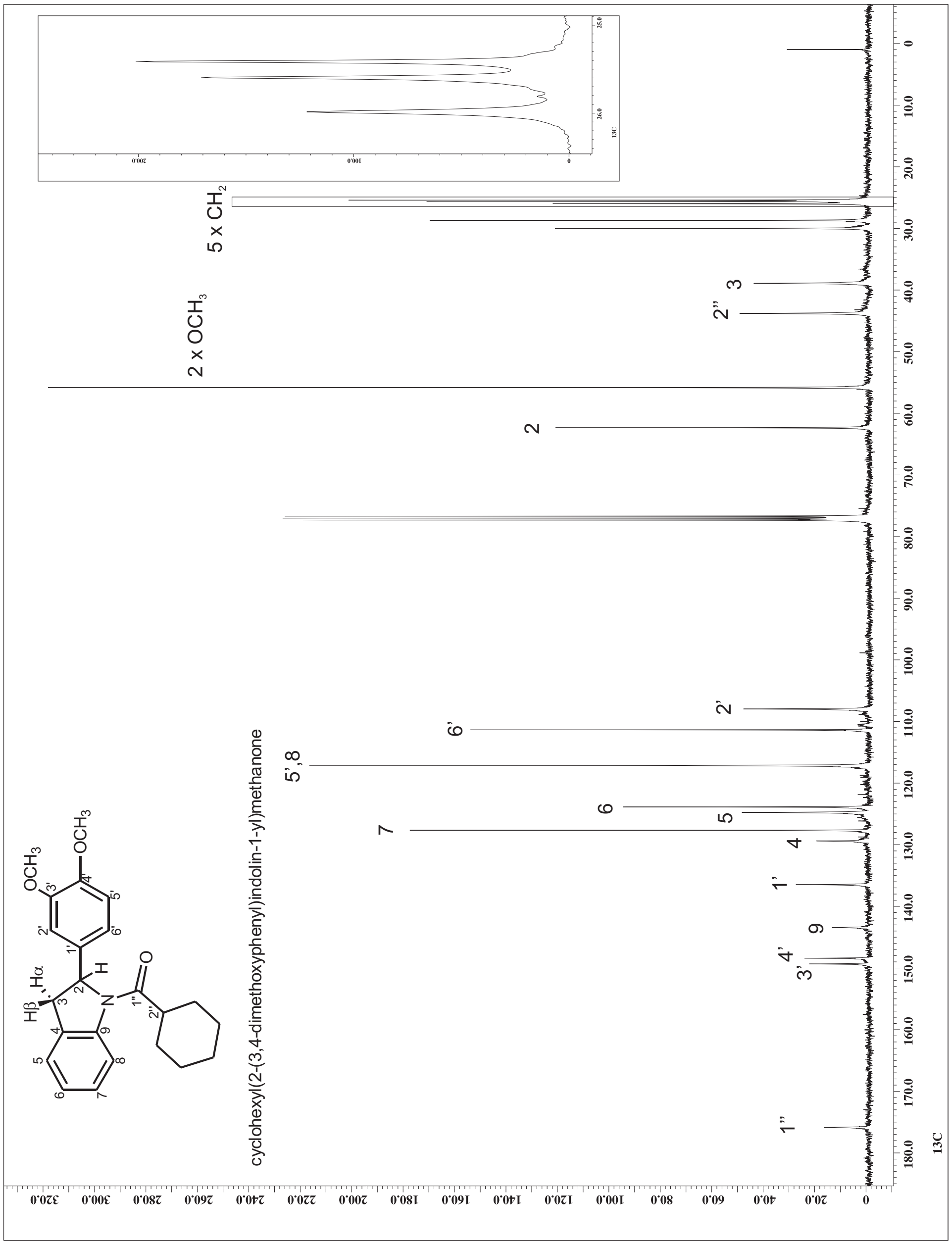

Figure S20: ^{13}C spectrum ($CDCl_3$, 100MHz) of **17**.

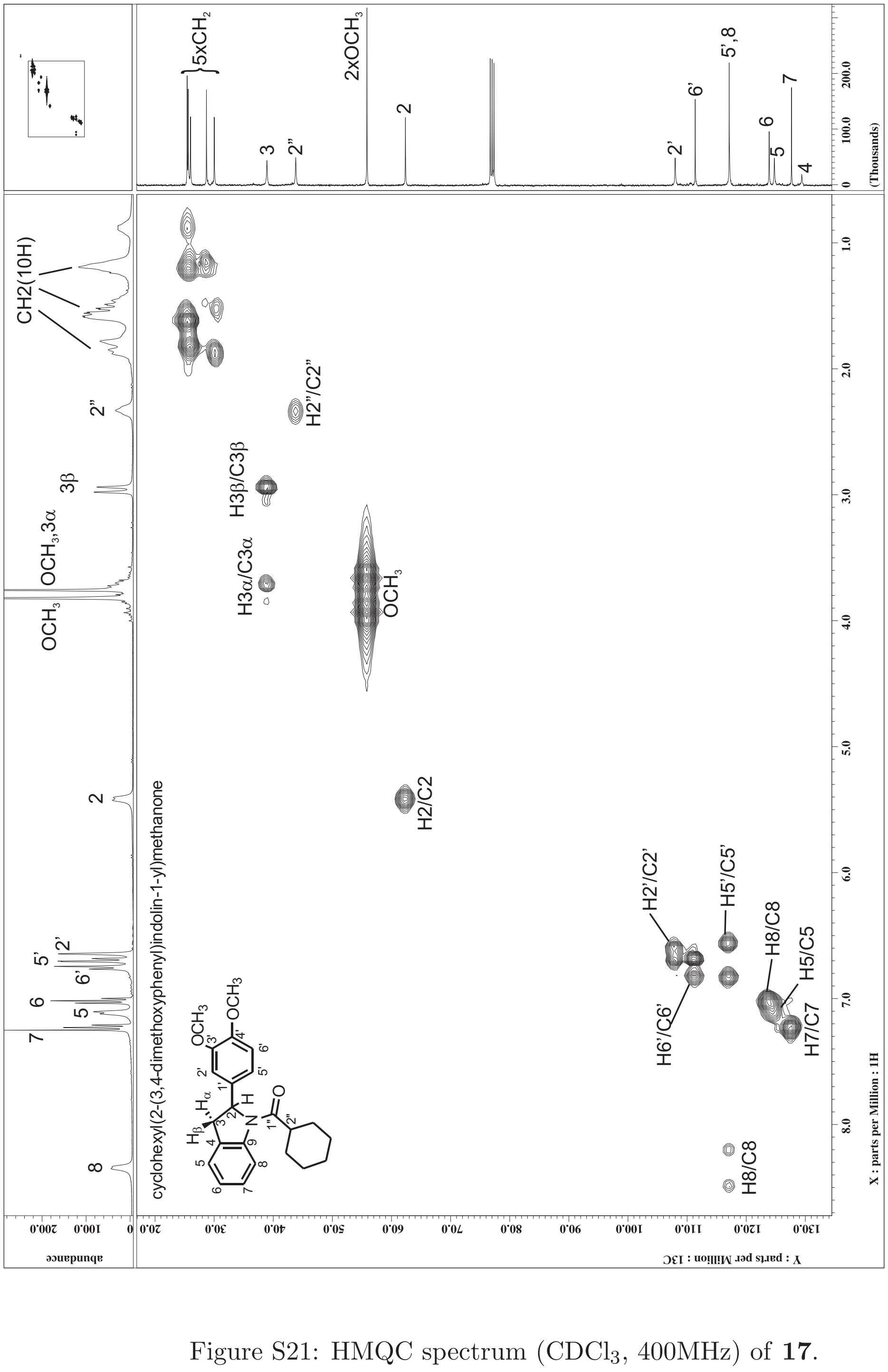

Figure S21: HMQC spectrum ($CDCl_3$, 400MHz) of **17**.

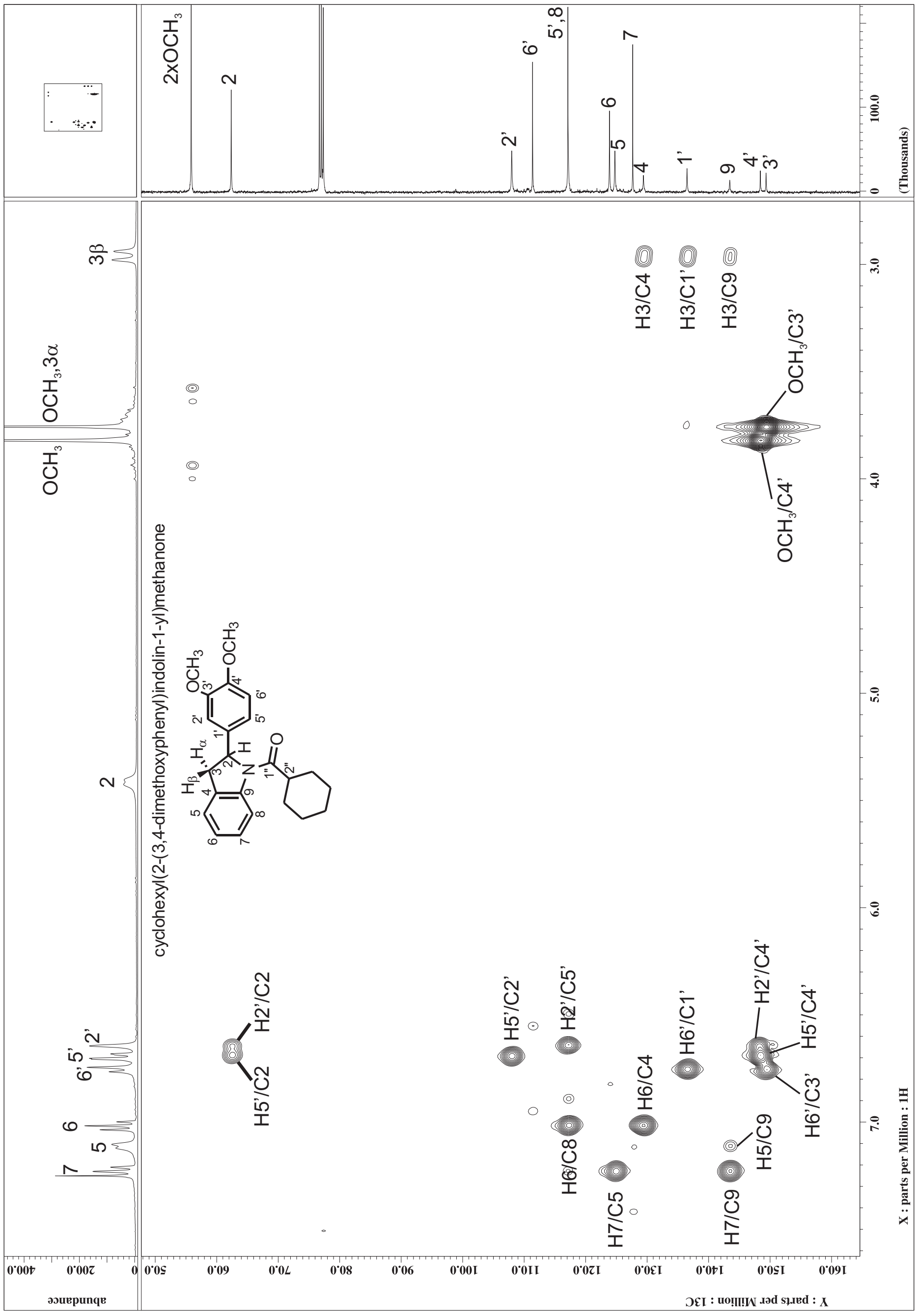

Figure S22: HMBC spectrum ($CDCl_3$, 400MHz) of **17**.

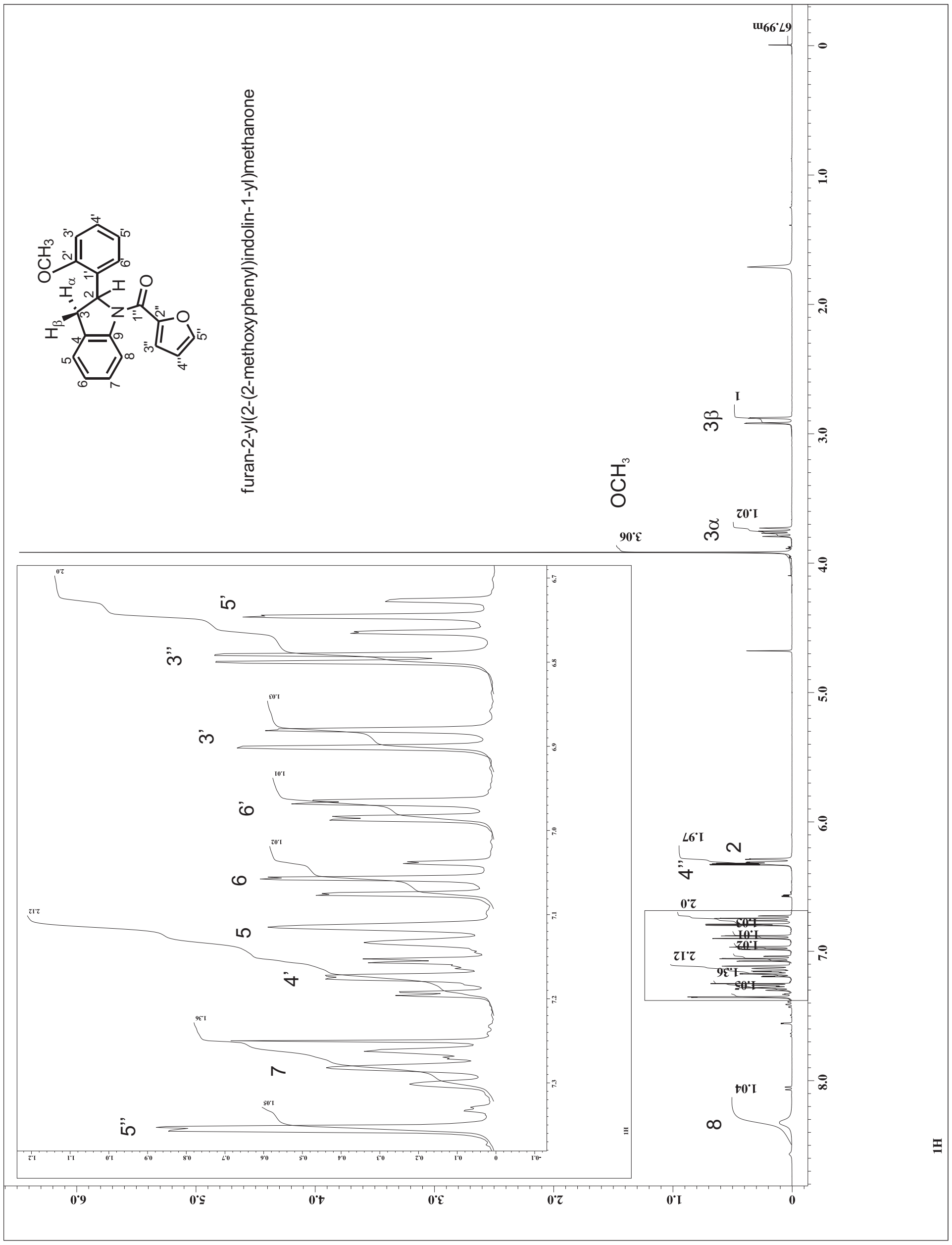

Figure S23: ^{1}H spectrum ($CDCl_3$, 400MHz) of **18**.

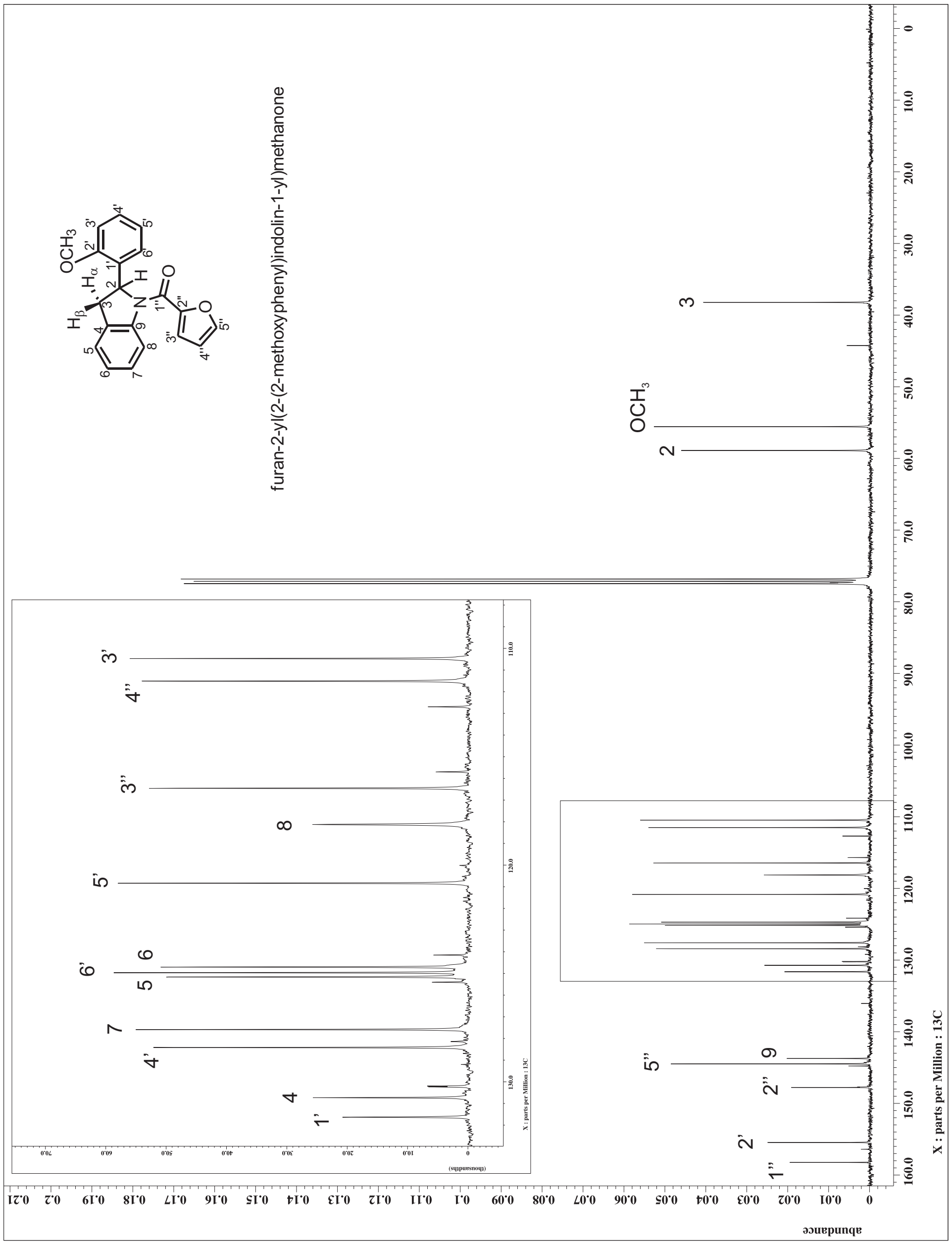

Figure S24: ^{13}C spectrum ($CDCl_3$, 100MHz) of **18**.

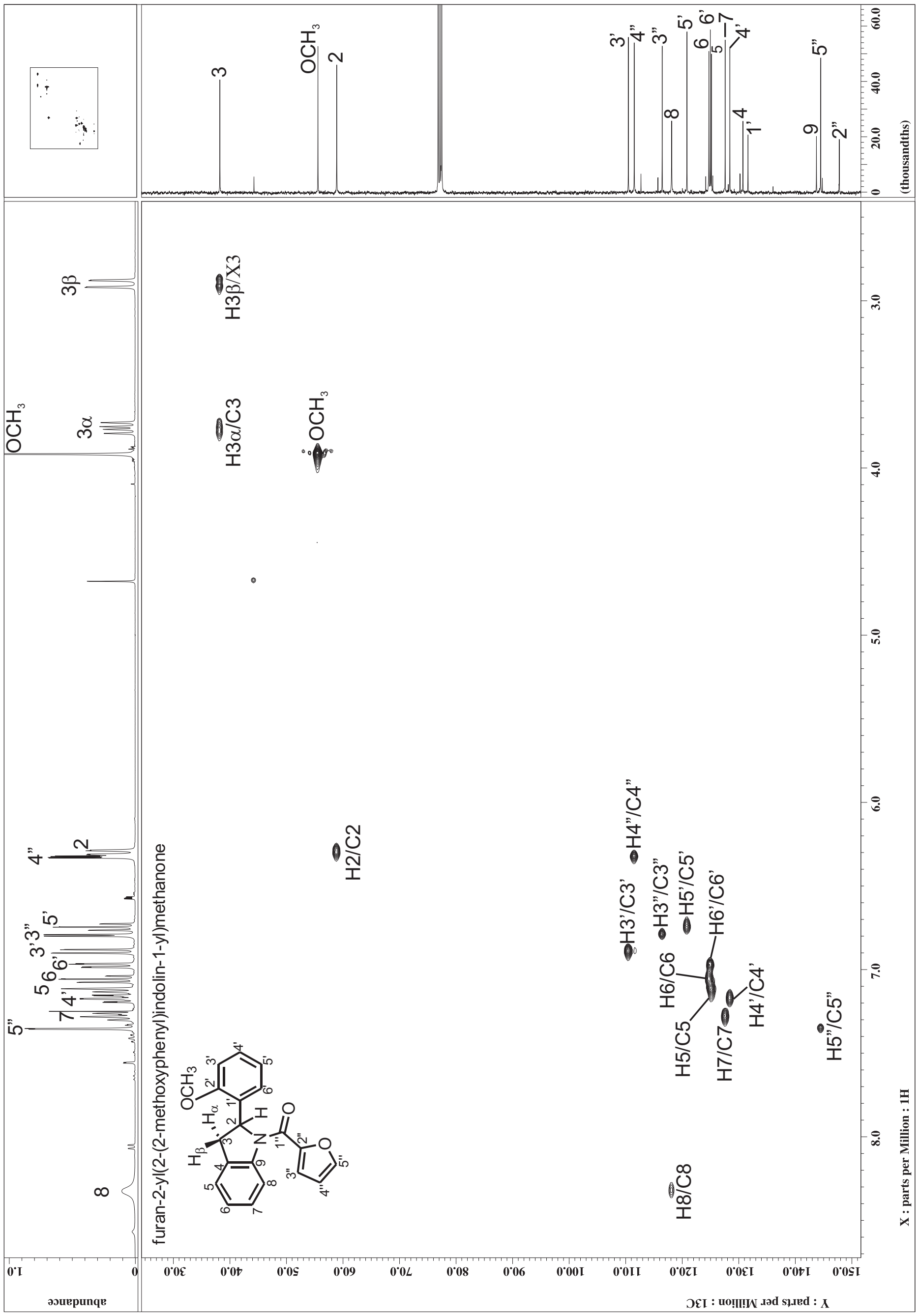

Figure S25: HSQC spectrum ($CDCl_3$, 400MHz) of **18**.

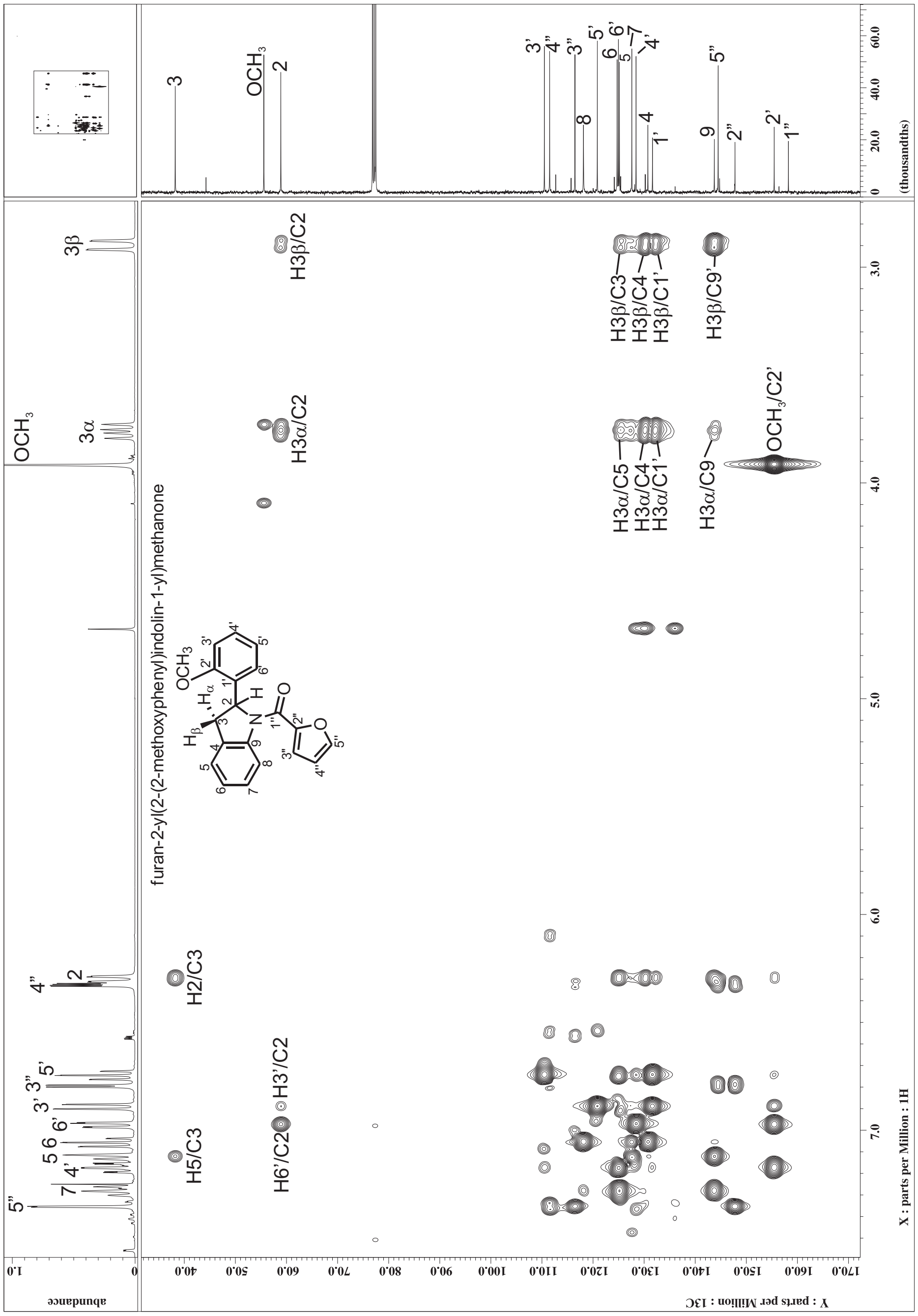

Figure S26: HMBC spectrum ($CDCl_3$, 400MHz) of **18**.

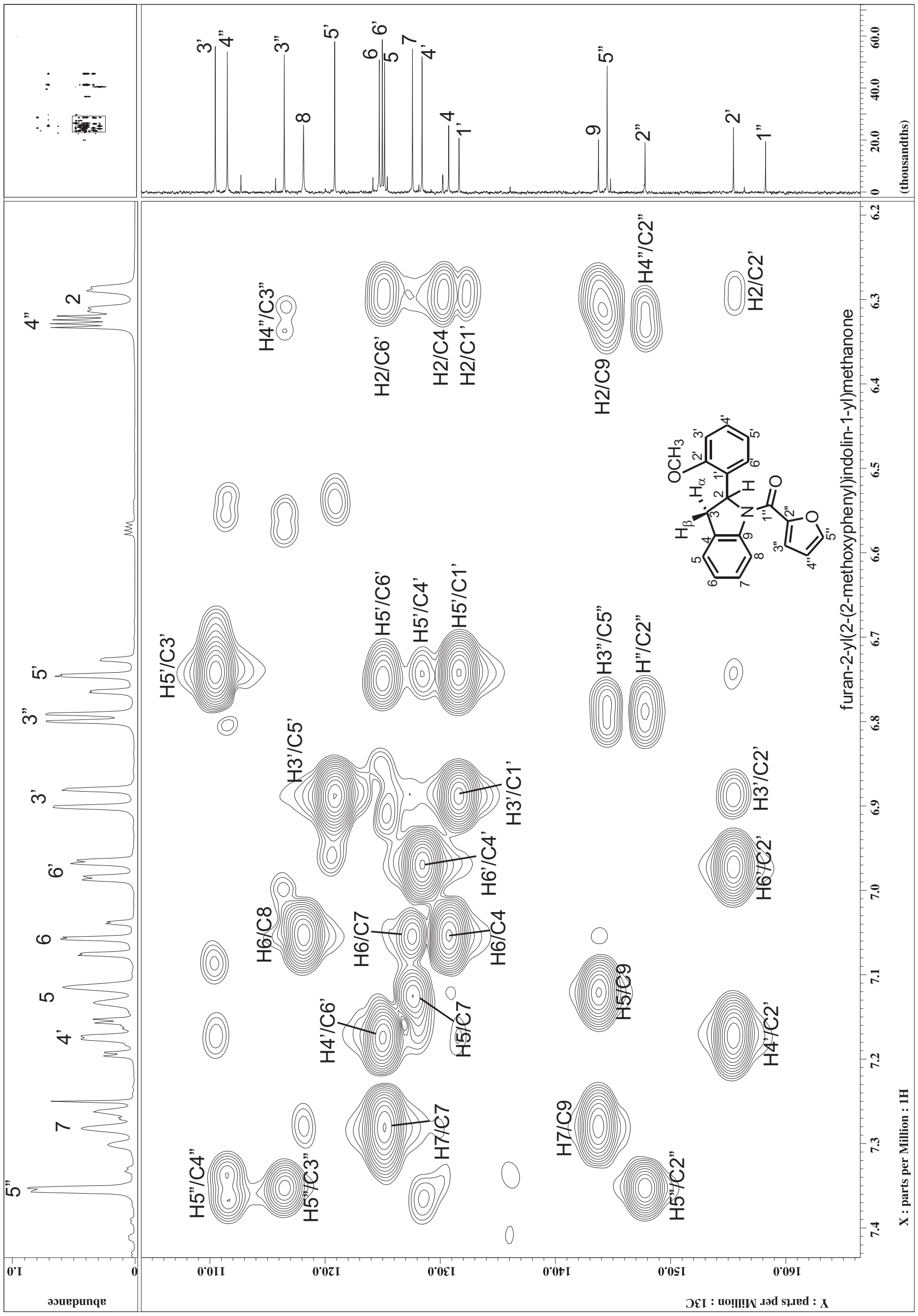

Figure S27: HMBC spectrum ($CDCl_3$, 400MHz) of **18** (expanded).

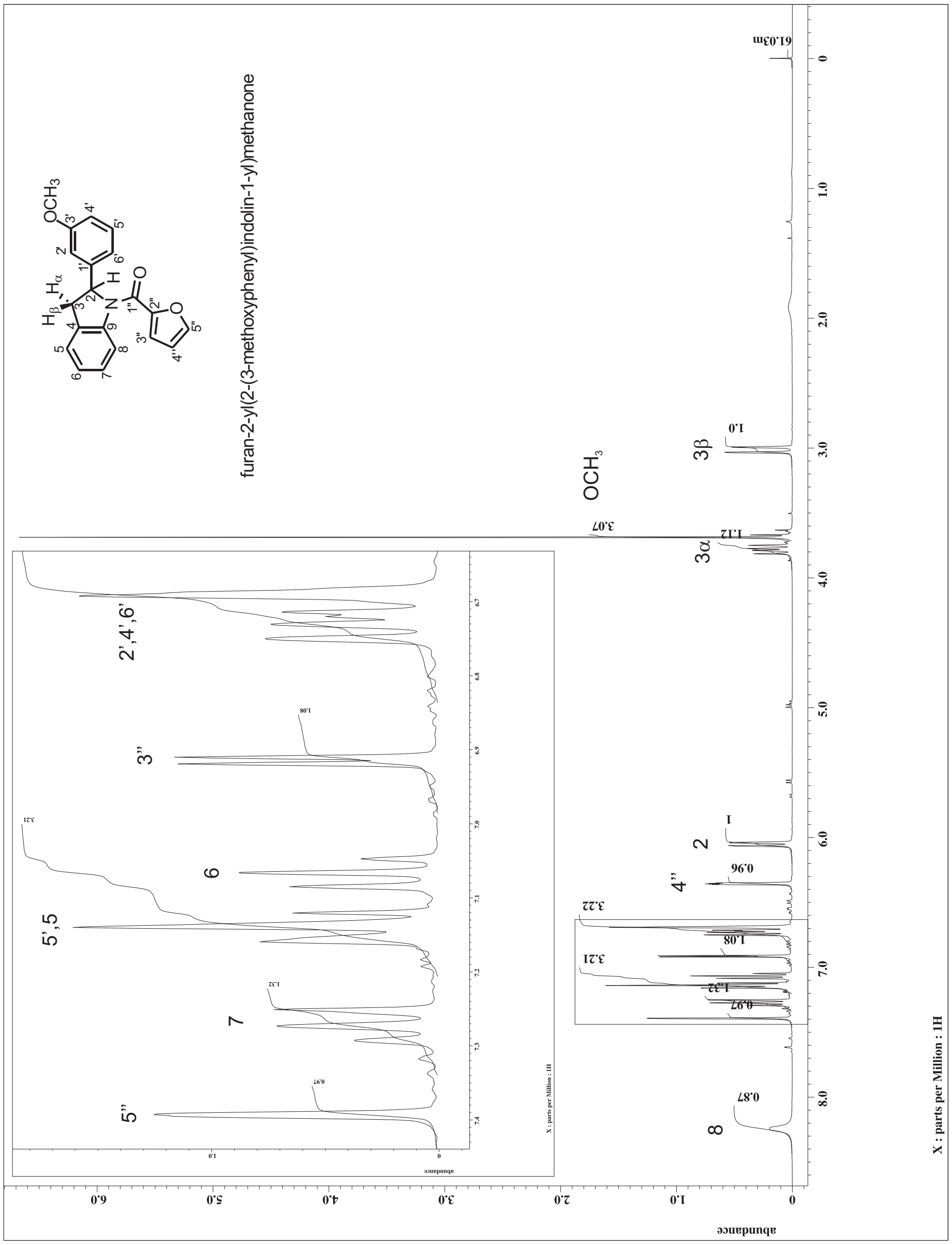

Figure S28: ^{1}H spectrum ($CDCl_3$, 400MHz) of **19**.

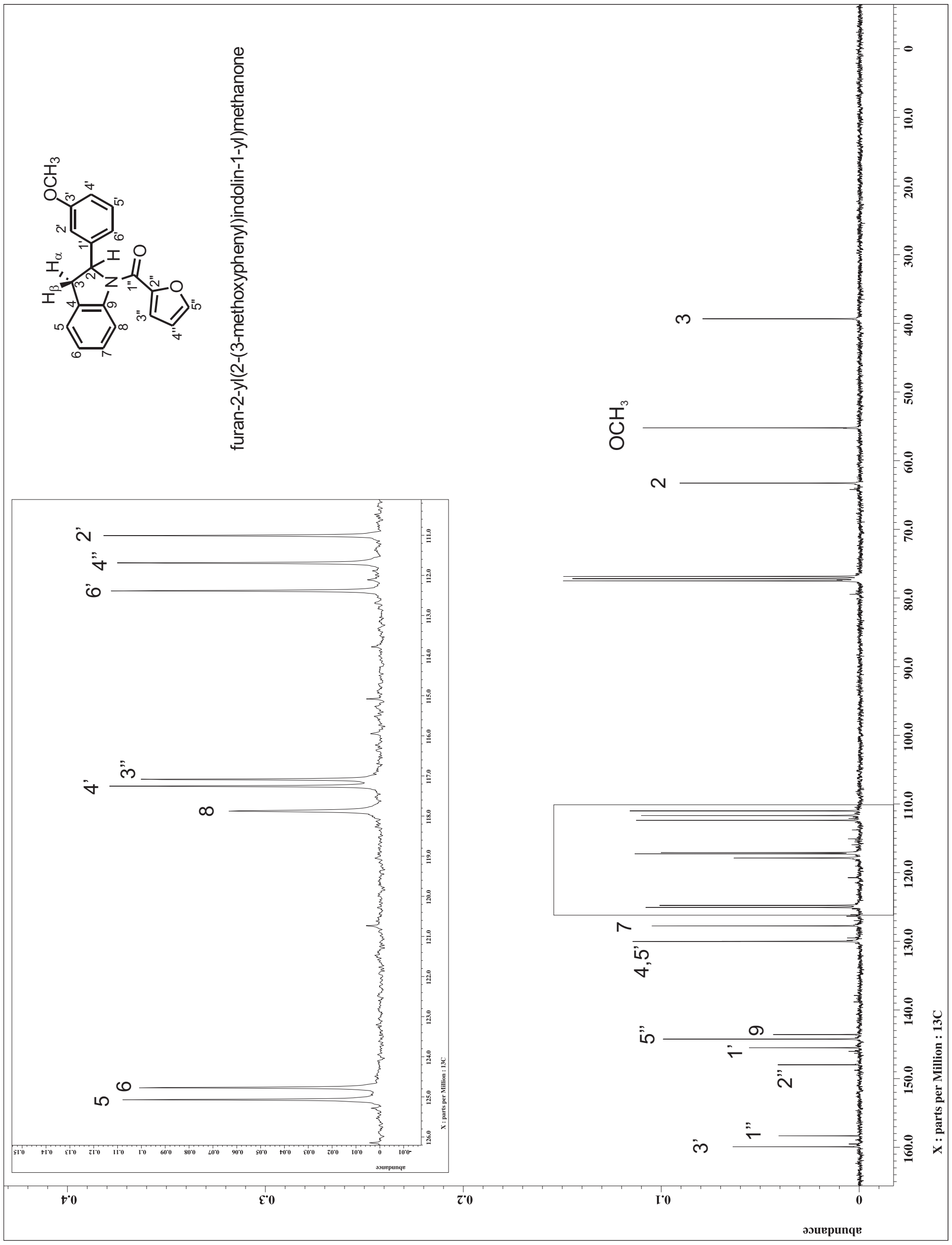

Figure S29: ^{13}C spectrum ($CDCl_3$, 100MHz) of **19**.

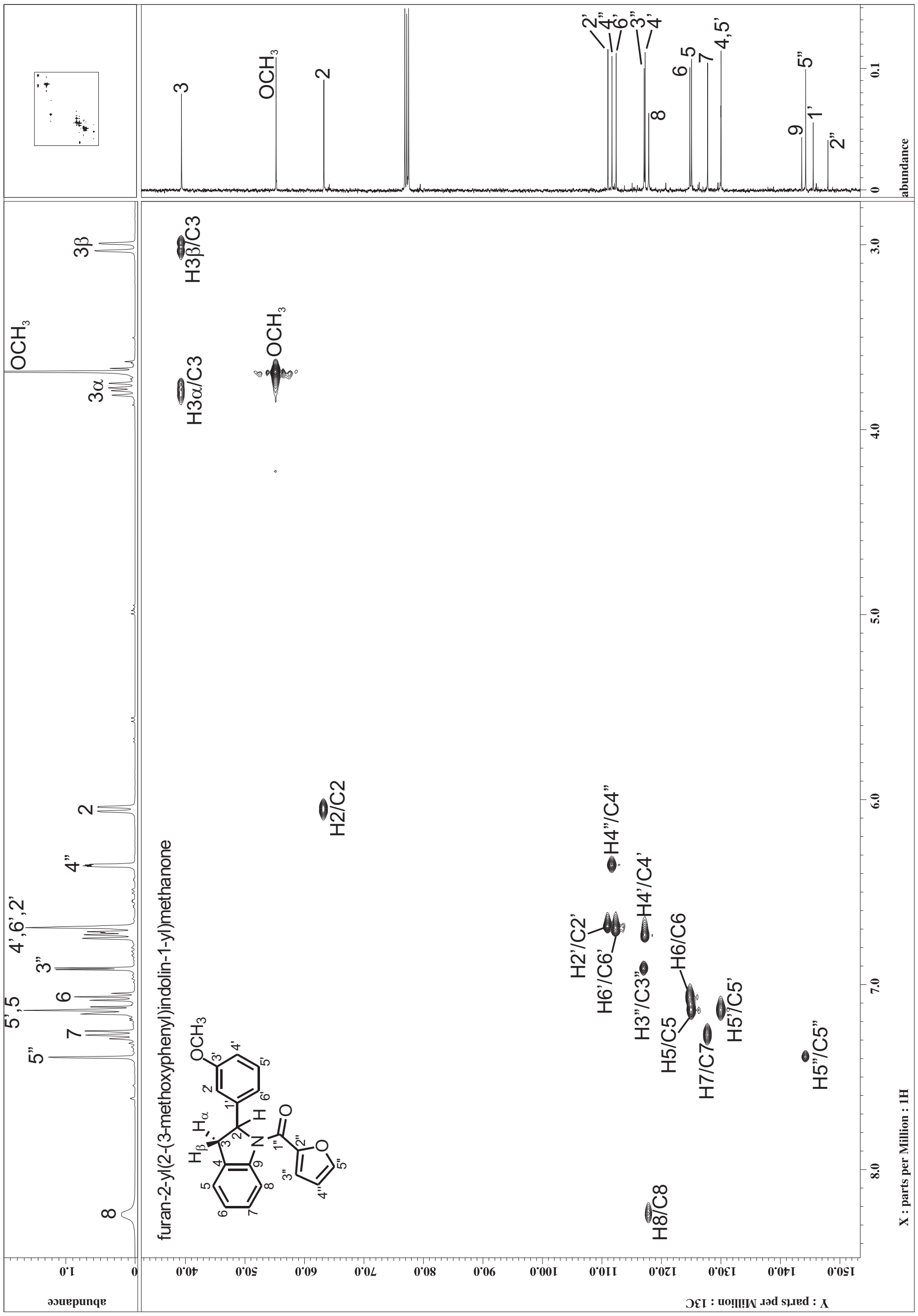

Figure S30: HSQC spectrum ($CDCl_3$, 400MHz) of **19**.

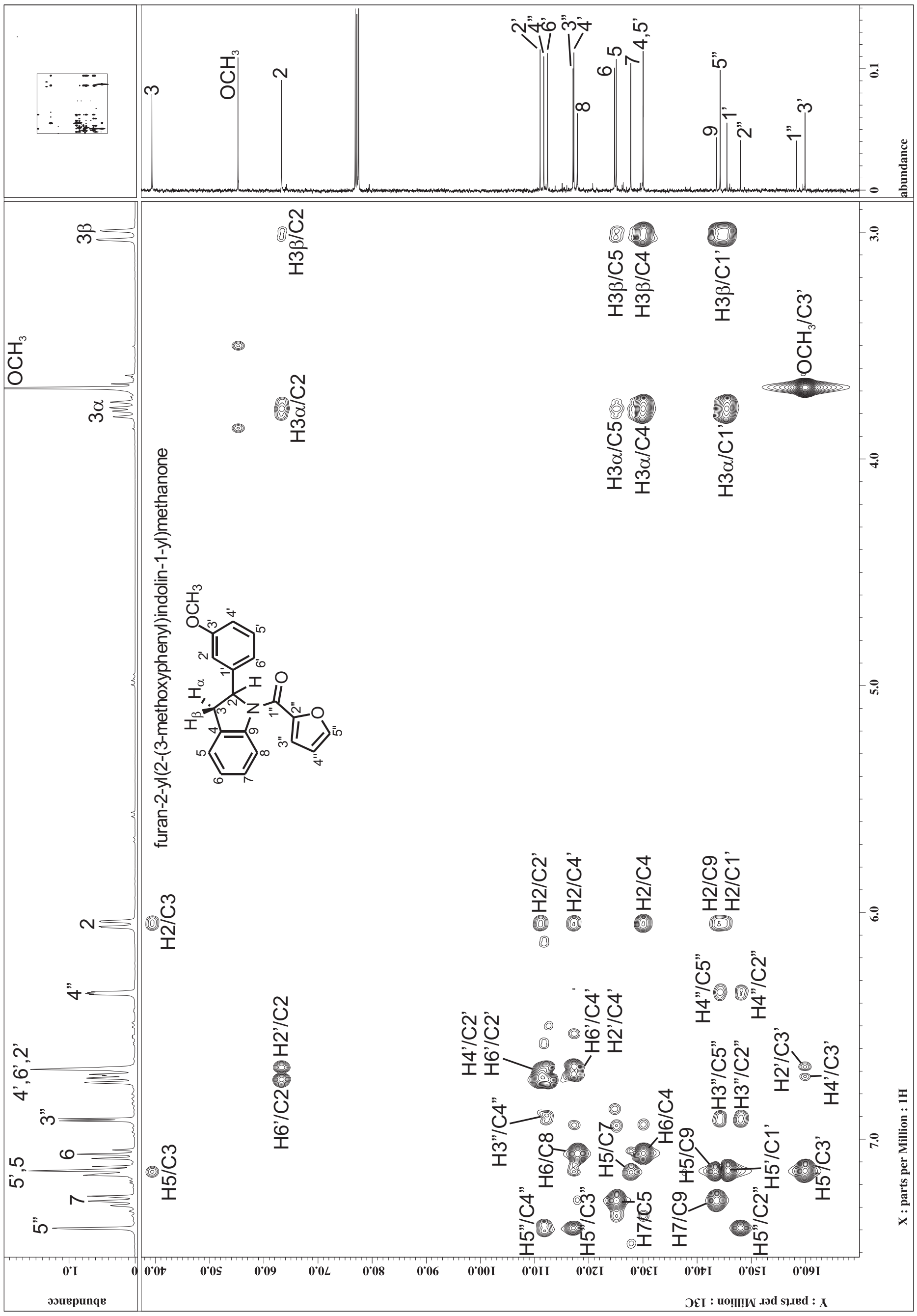

Figure S31: HMBC spectrum ($CDCl_3$, 400MHz) of **19**.

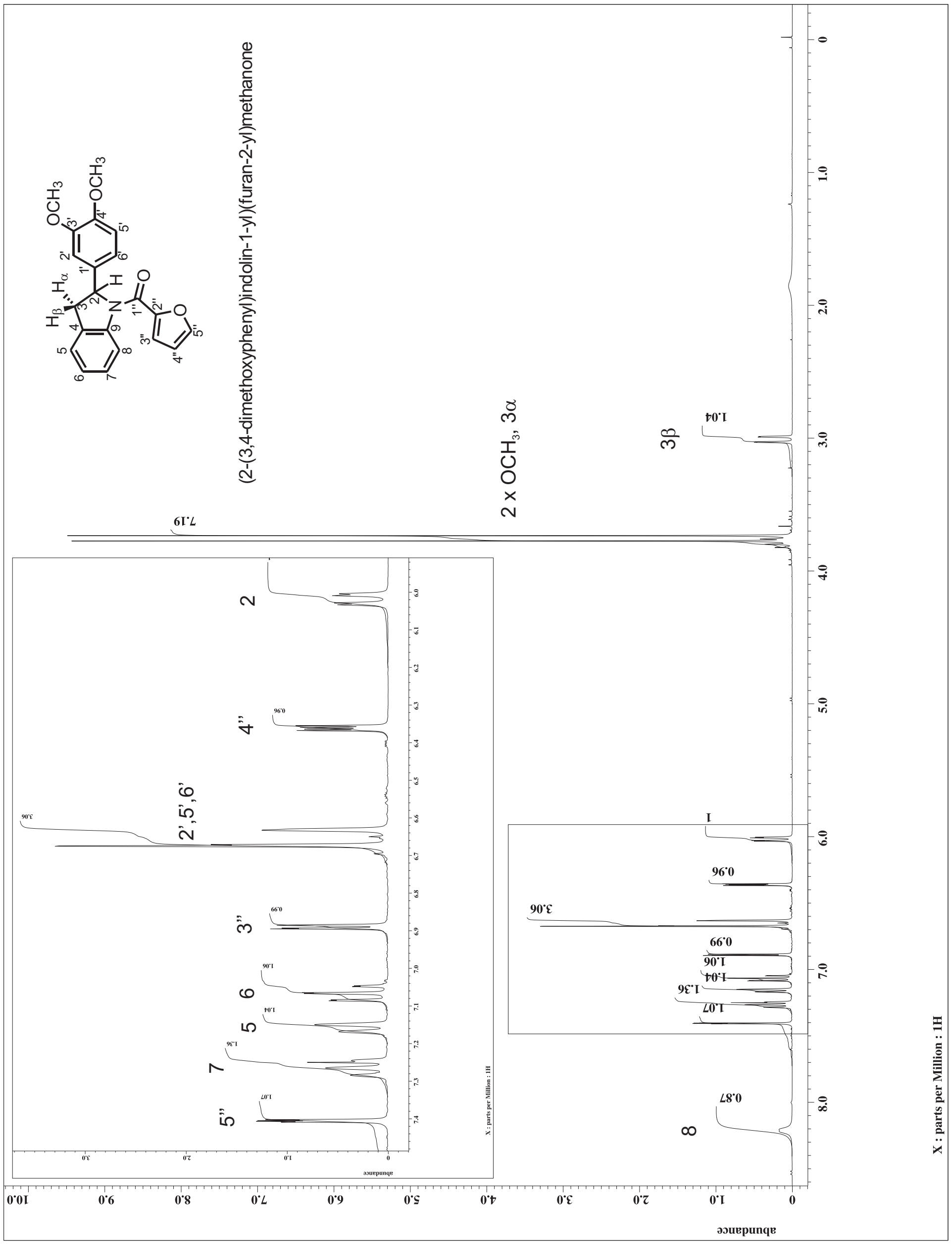

Figure S32: ^{1}H spectrum ($CDCl_3$, 400MHz) of **20a**.

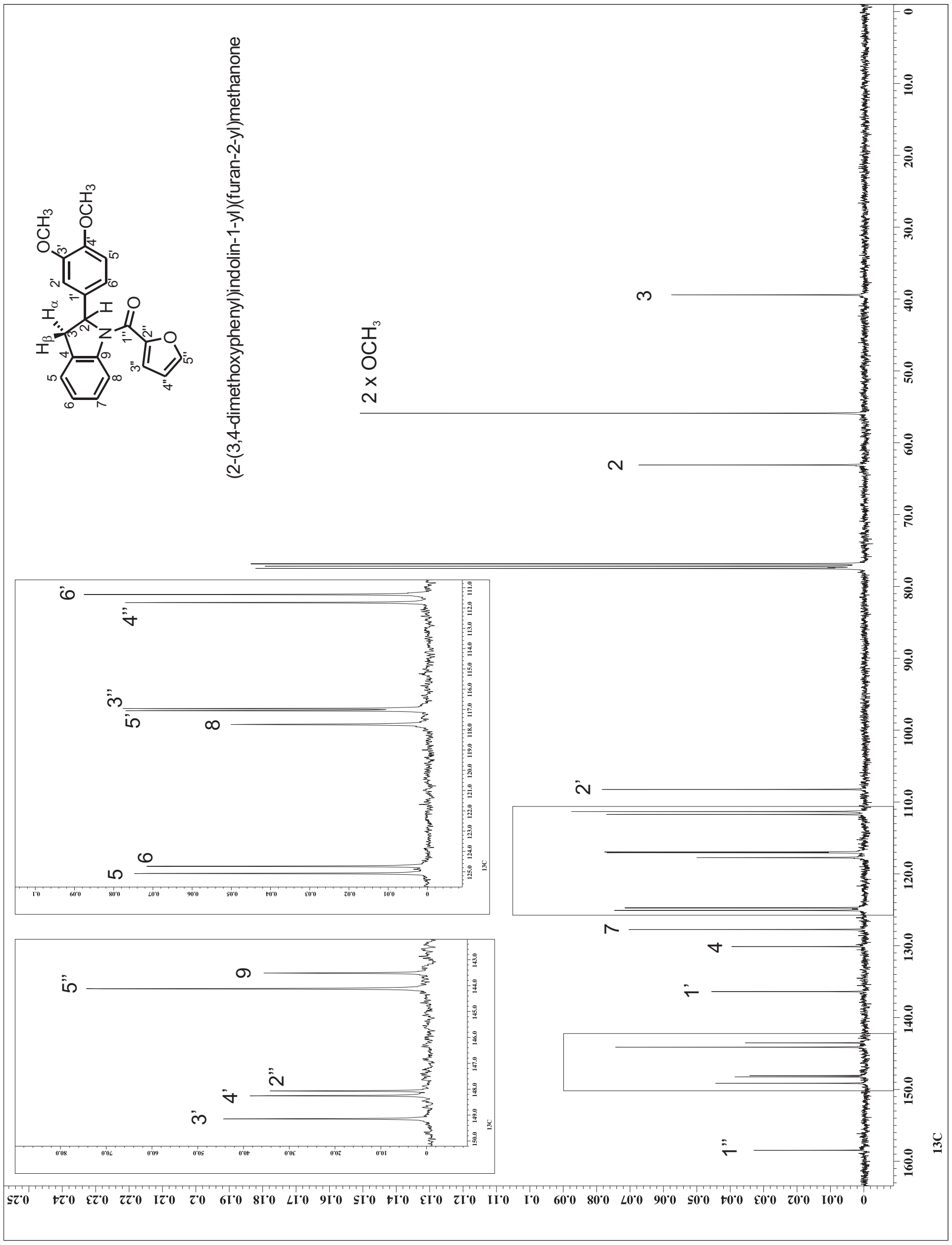

Figure S33: ^{13}C spectrum ($CDCl_3$, 100MHz) of **20a**.

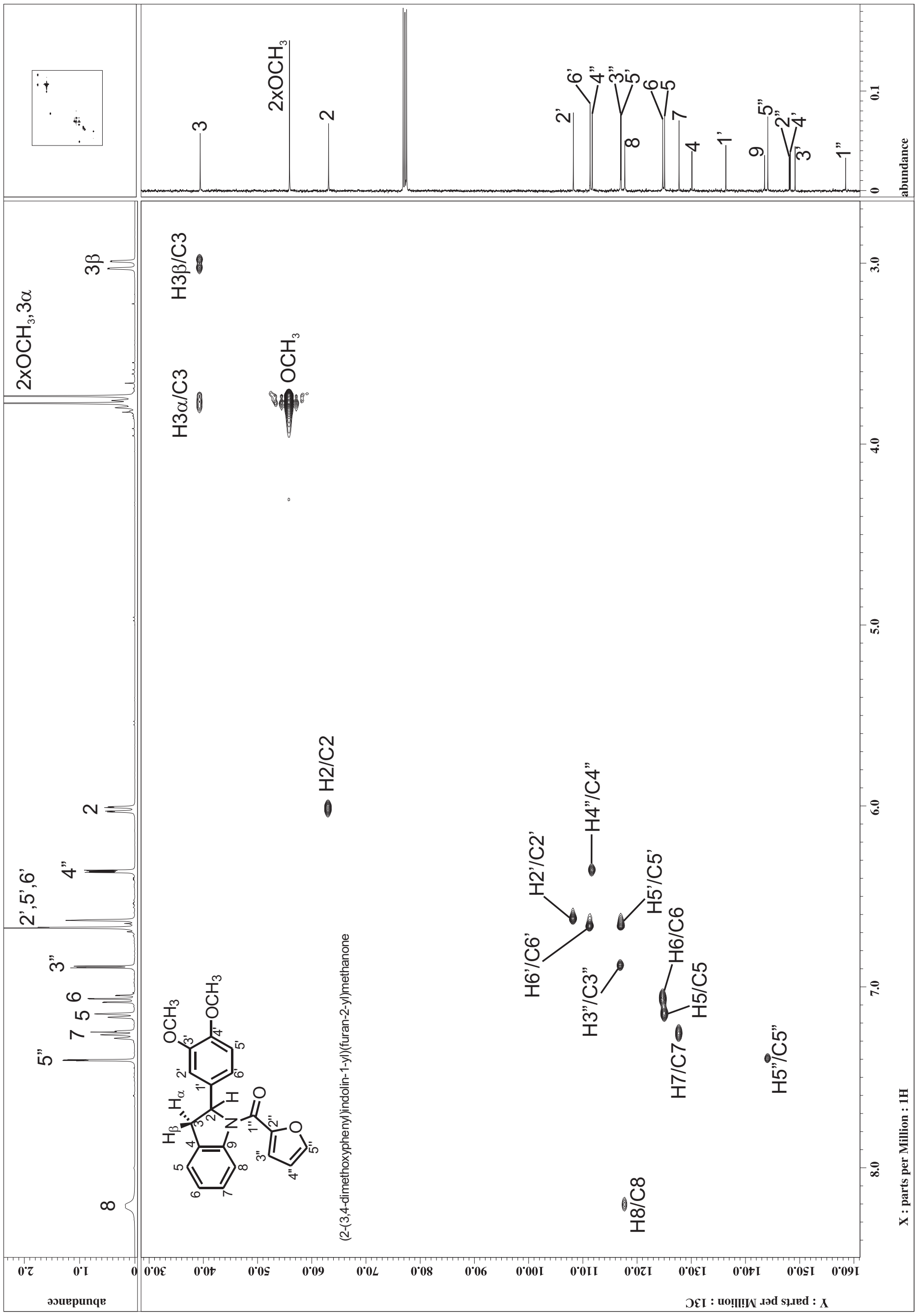

Figure S34: HSQC spectrum ($CDCl_3$, 400MHz) of **20a**.

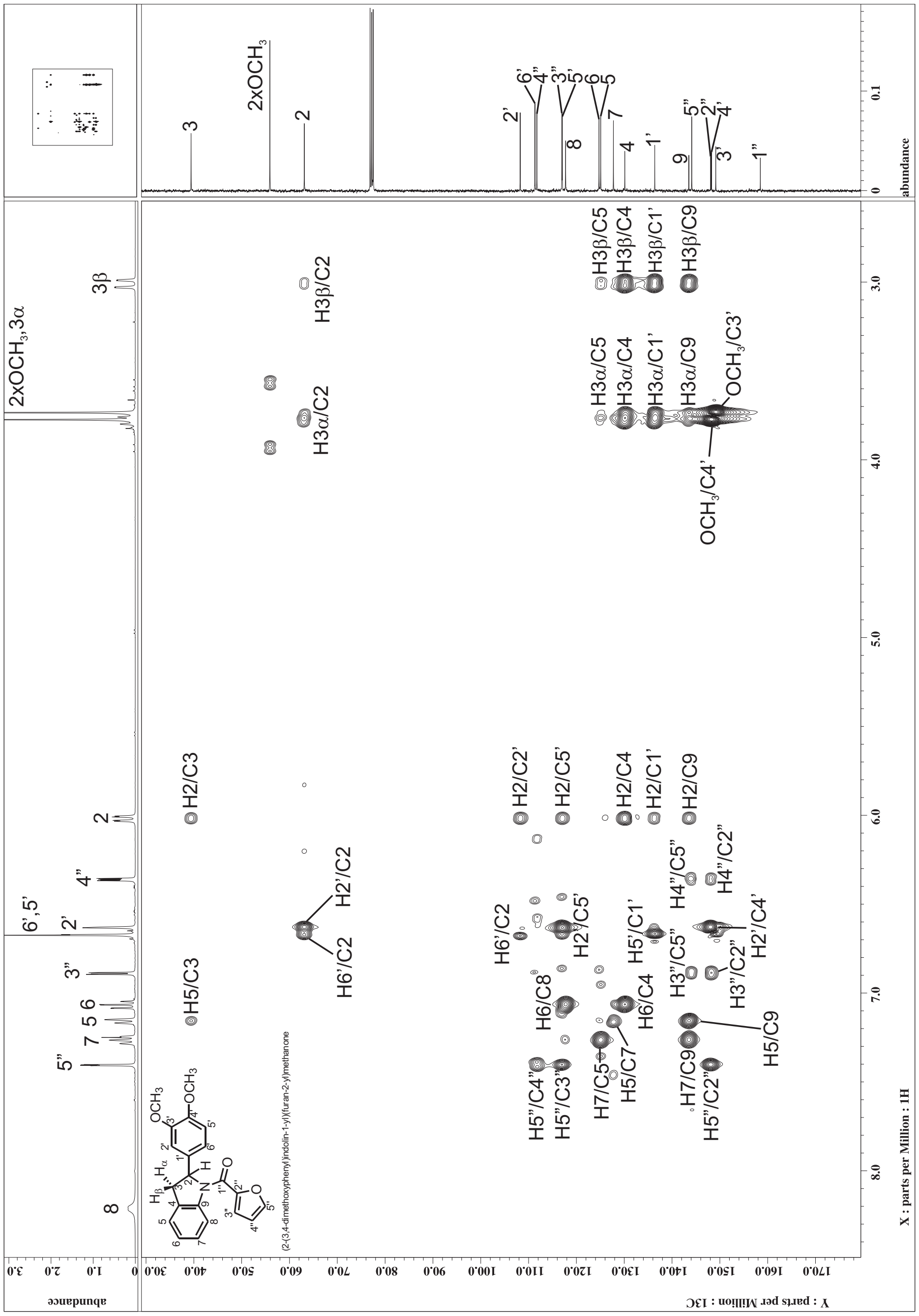

Figure S35: HMBC spectrum ($CDCl_3$, 400MHz) of **20a**.

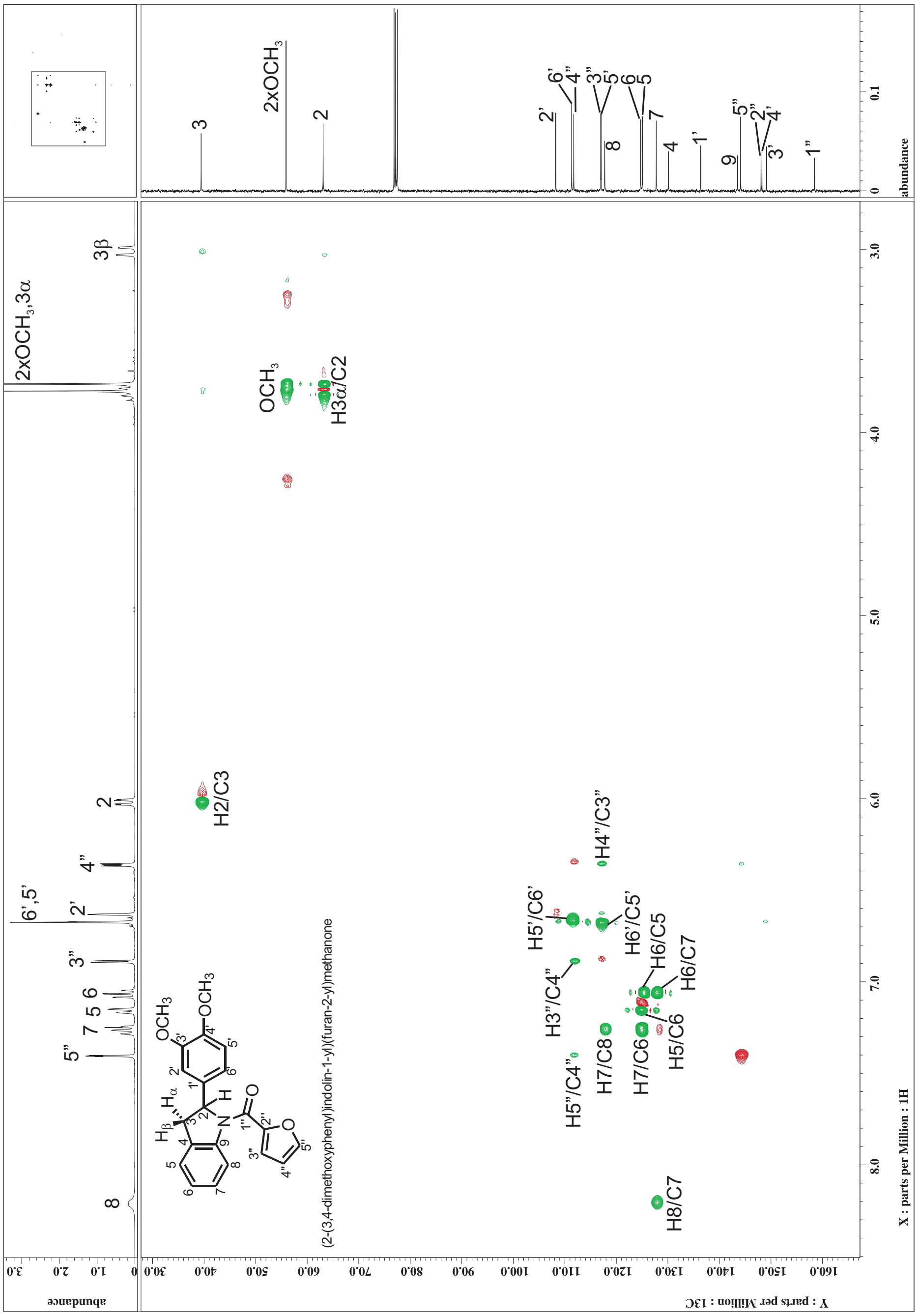

Figure S36: H2BC spectrum ($CDCl_3$, 400MHz) of **20a**.

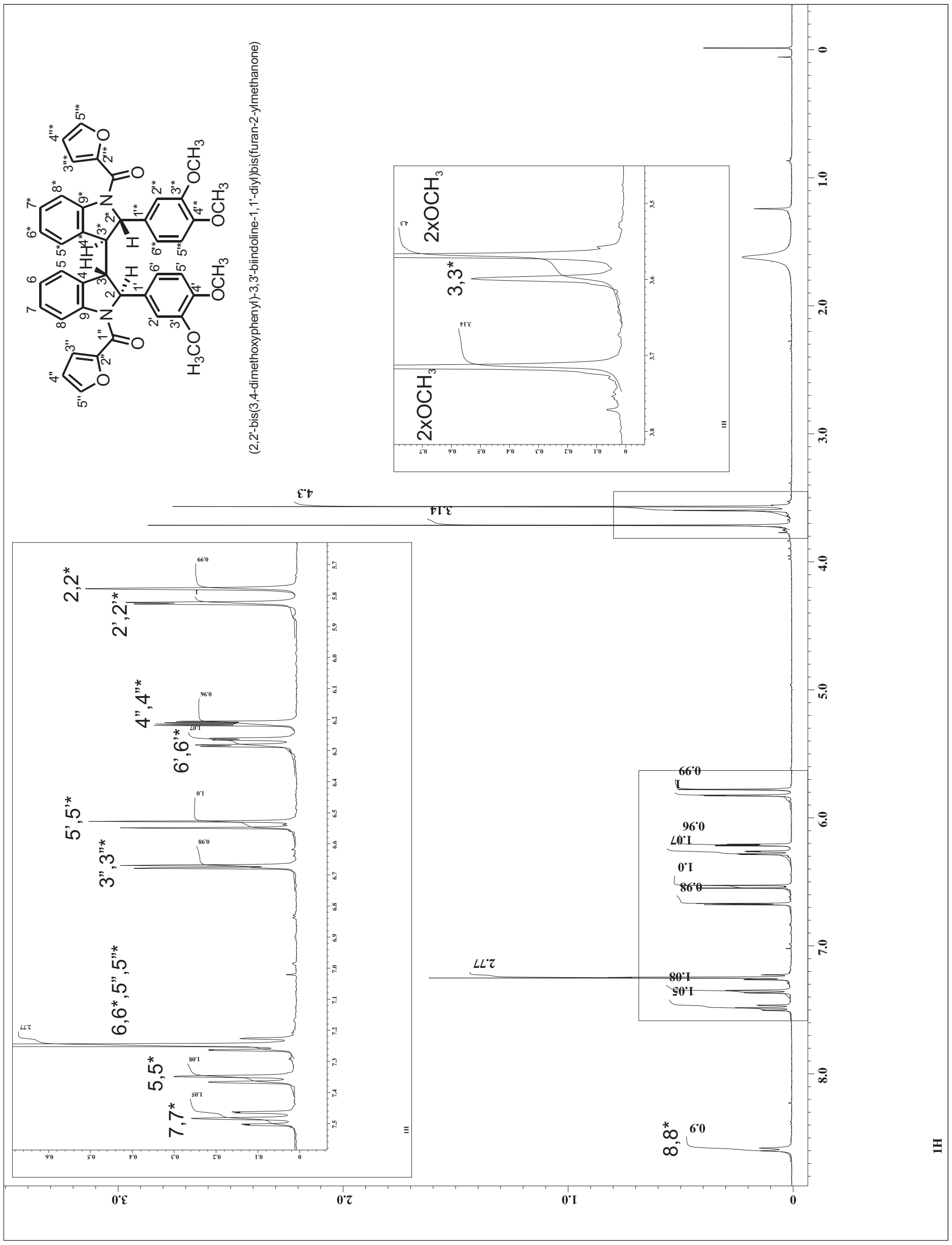

Figure S37: ^{1}H spectrum ($CDCl_3$, 400MHz) of **(±)20b**.

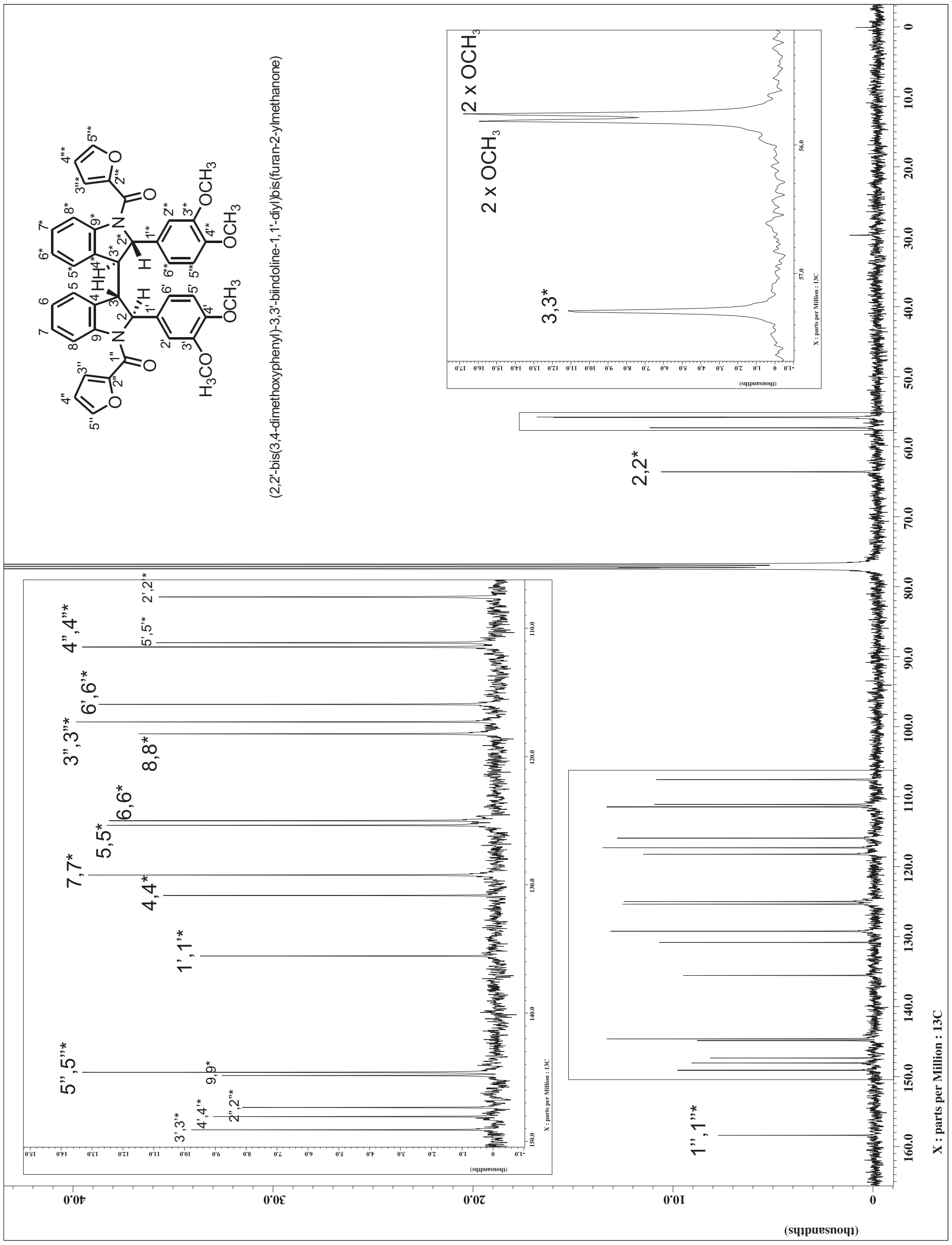

Figure S38: ^{13}C spectrum ($CDCl_3$, 100MHz) of **(±)20b**.

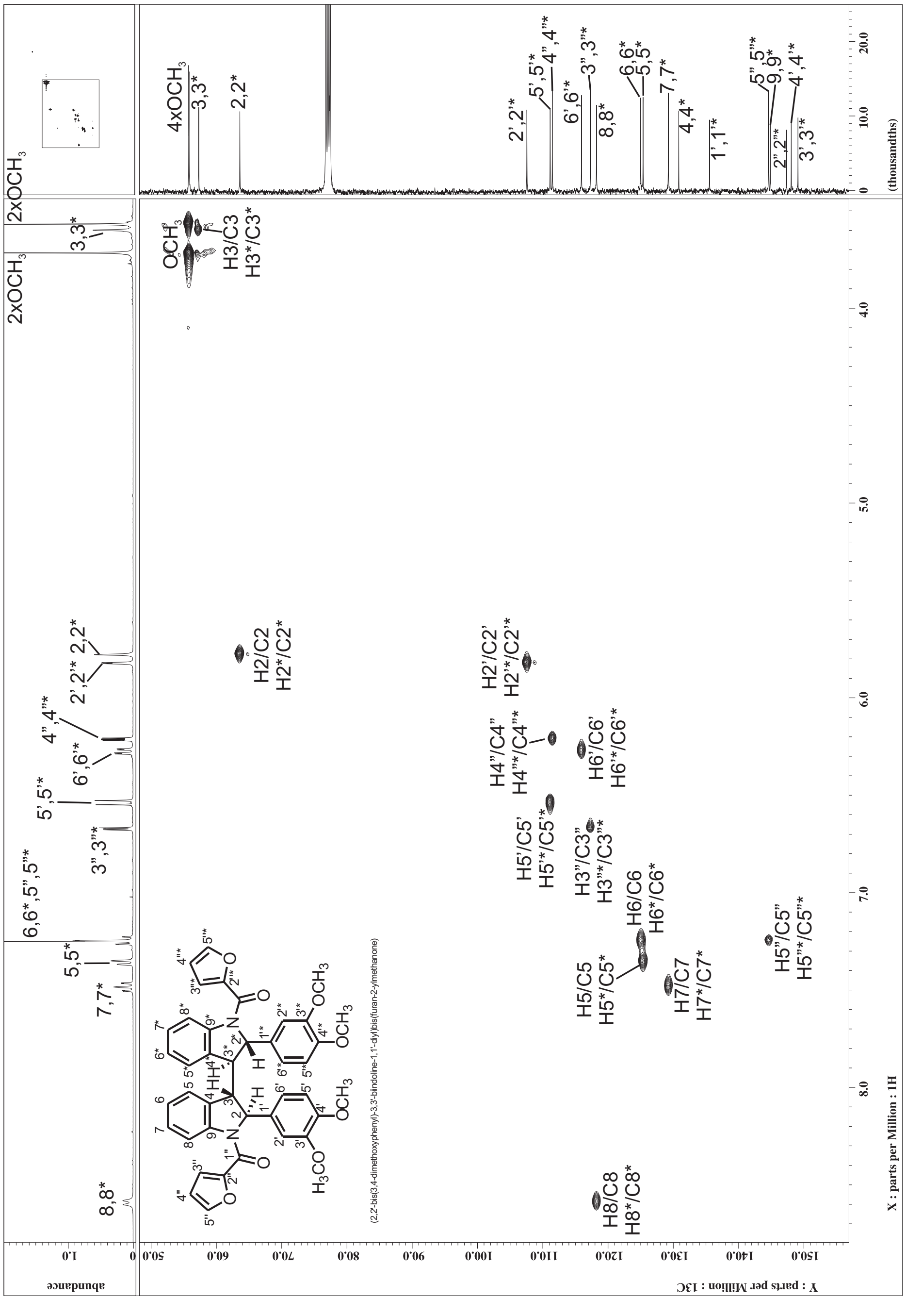

Figure S39: HSQC spectrum ($CDCl_3$, 400MHz) of **(±)20b**.

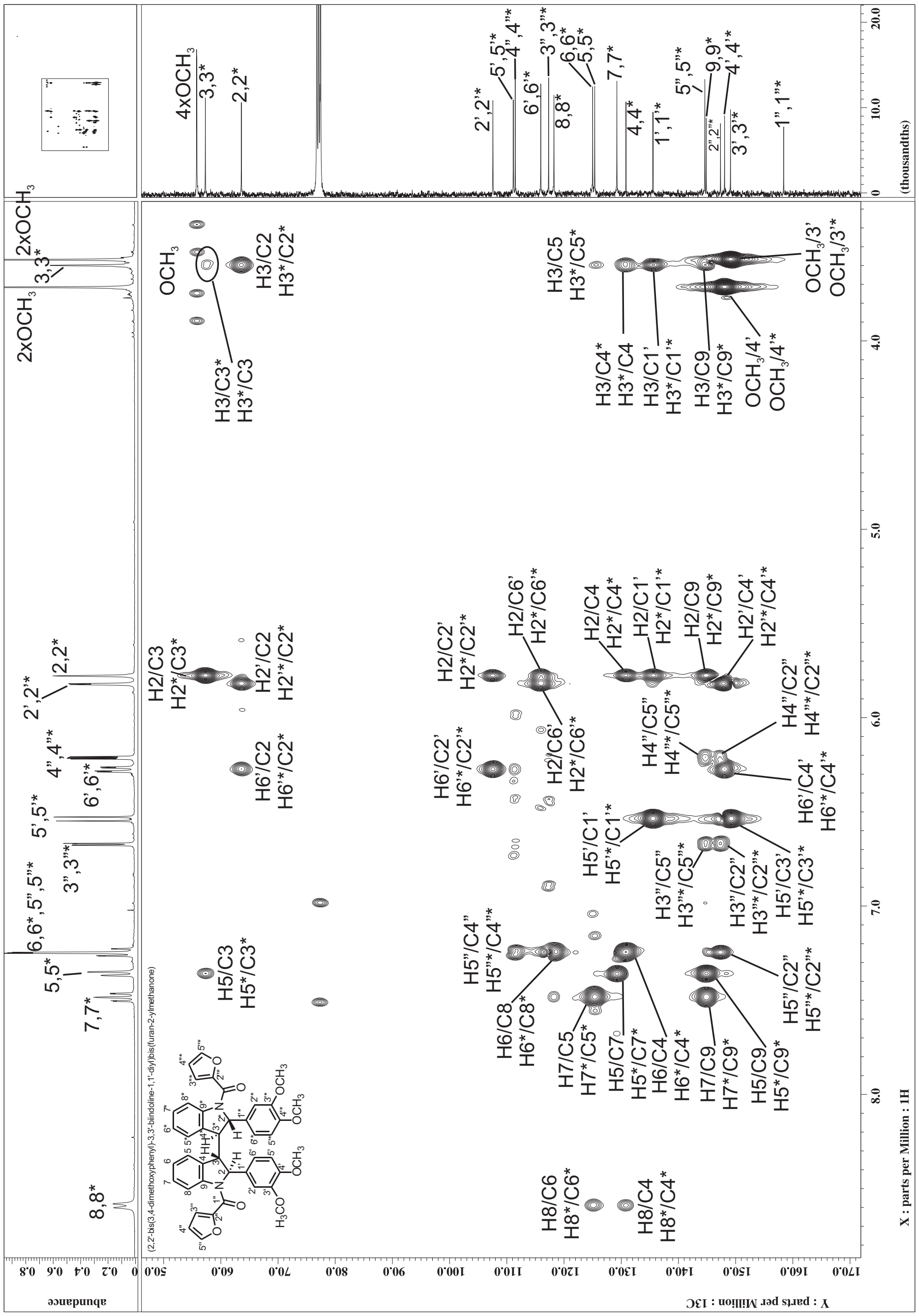

Figure S40: HMBC spectrum ($CDCl_3$, 400MHz) of **(±)20b**.

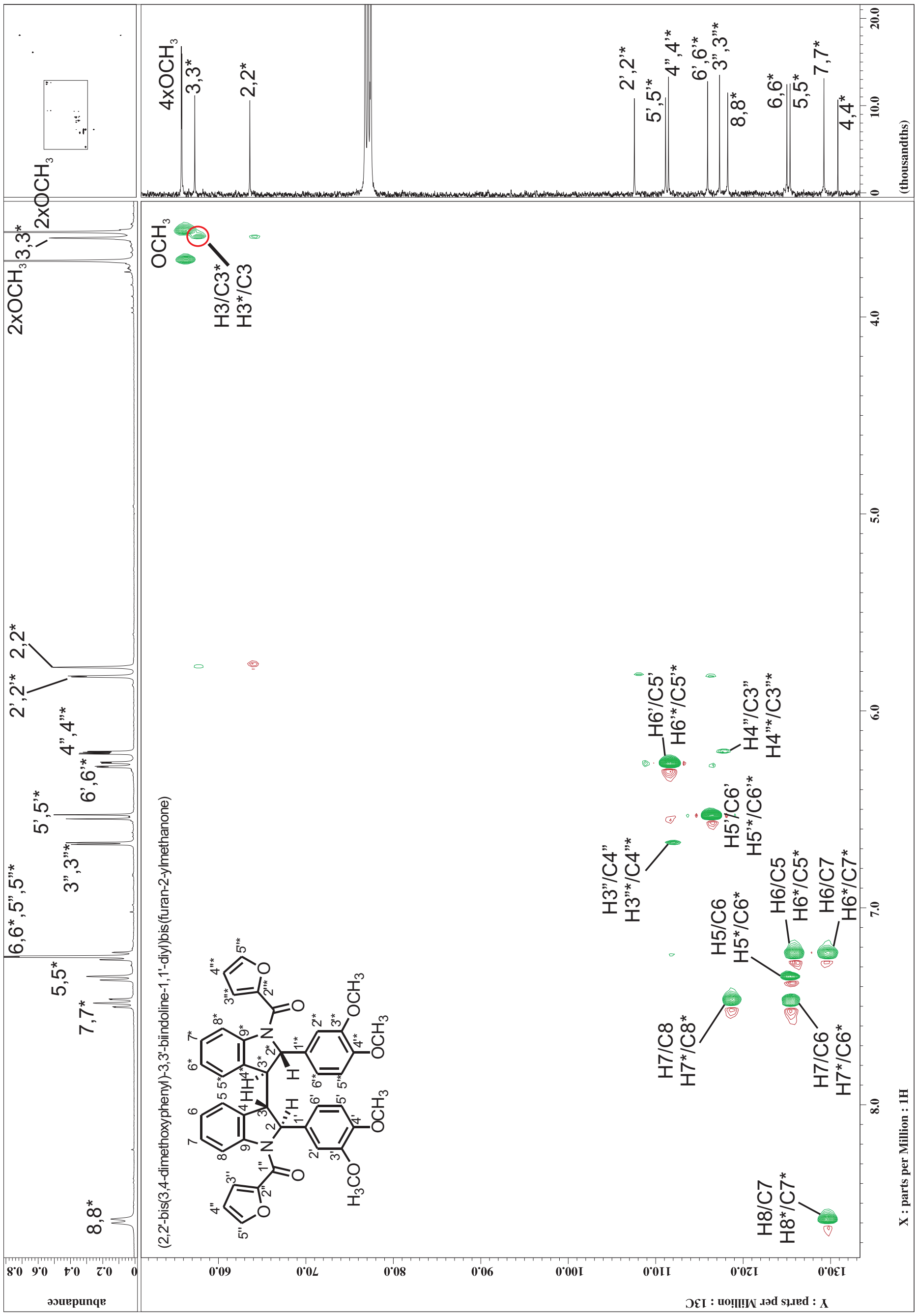

Figure S41: H2BC spectrum ($CDCl_3$, 400MHz) of **(±)20b**.

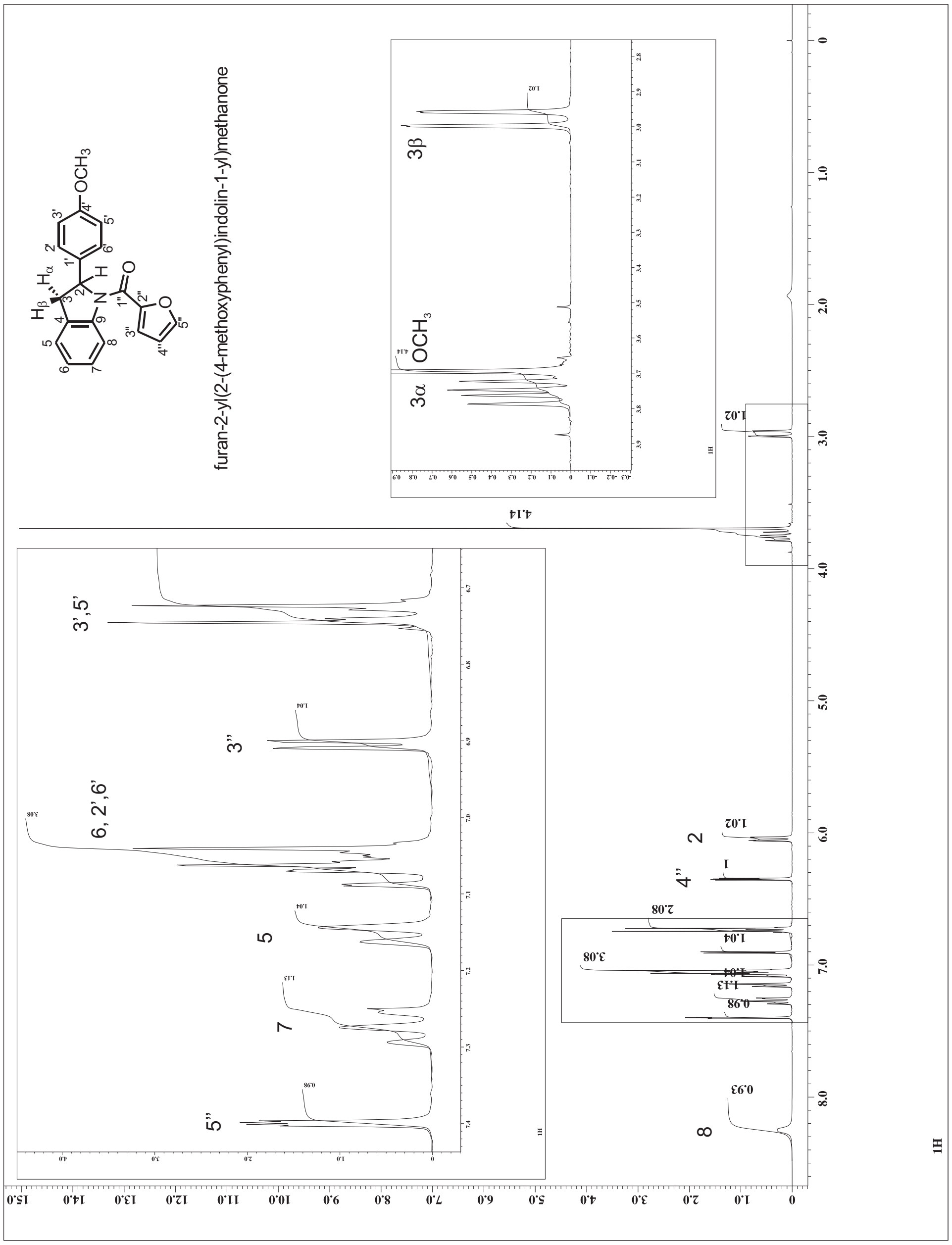

Figure S42: ^{1}H spectrum ($CDCl_3$, 400MHz) of **21a**.

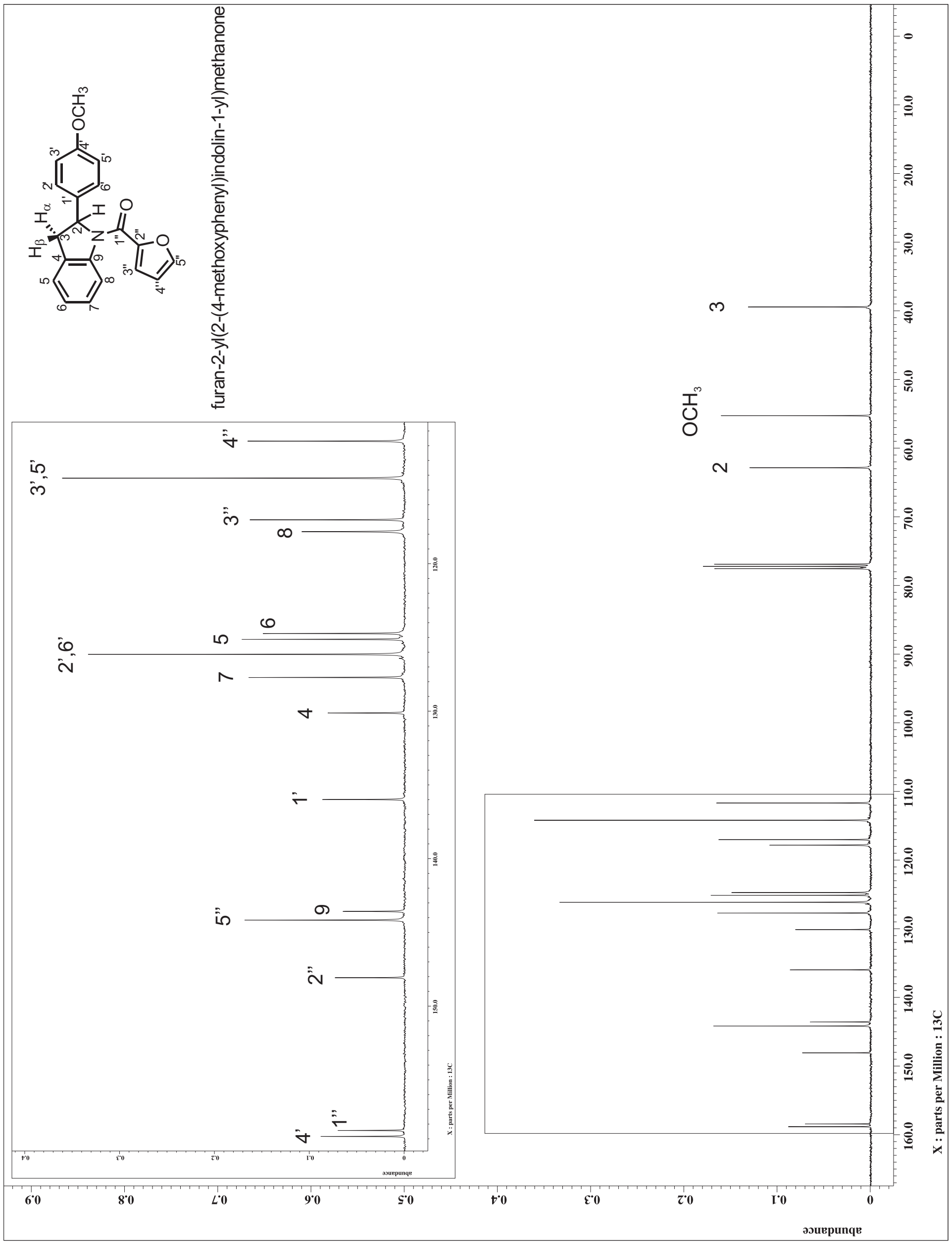

Figure S43: ^{13}C spectrum ($CDCl_3$, 100MHz) of **21a**.

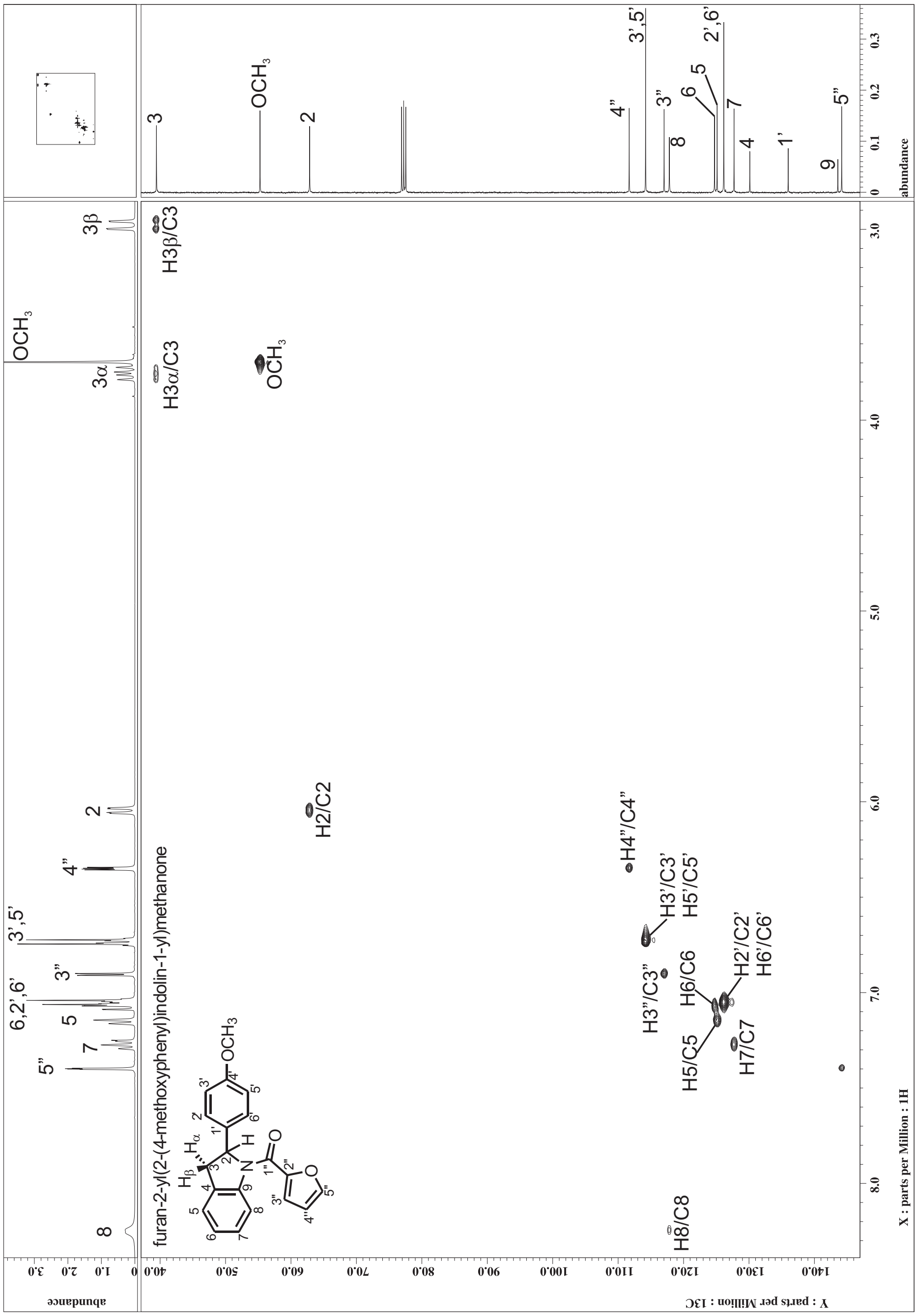

Figure S44: HSQC spectrum ($CDCl_3$, 400MHz) of **21a**.

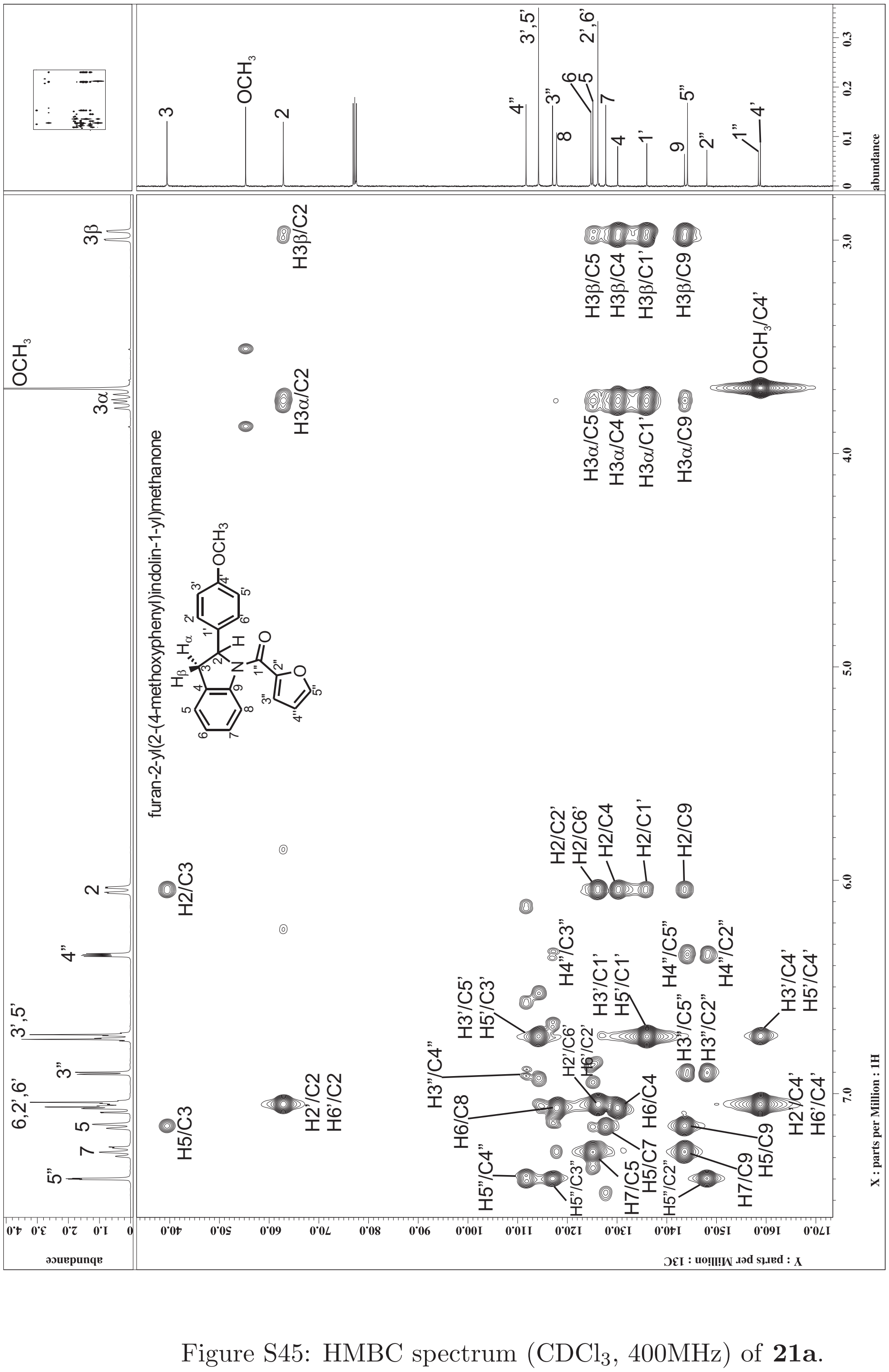

Figure S45: HMBC spectrum ($CDCl_3$, 400MHz) of **21a**.

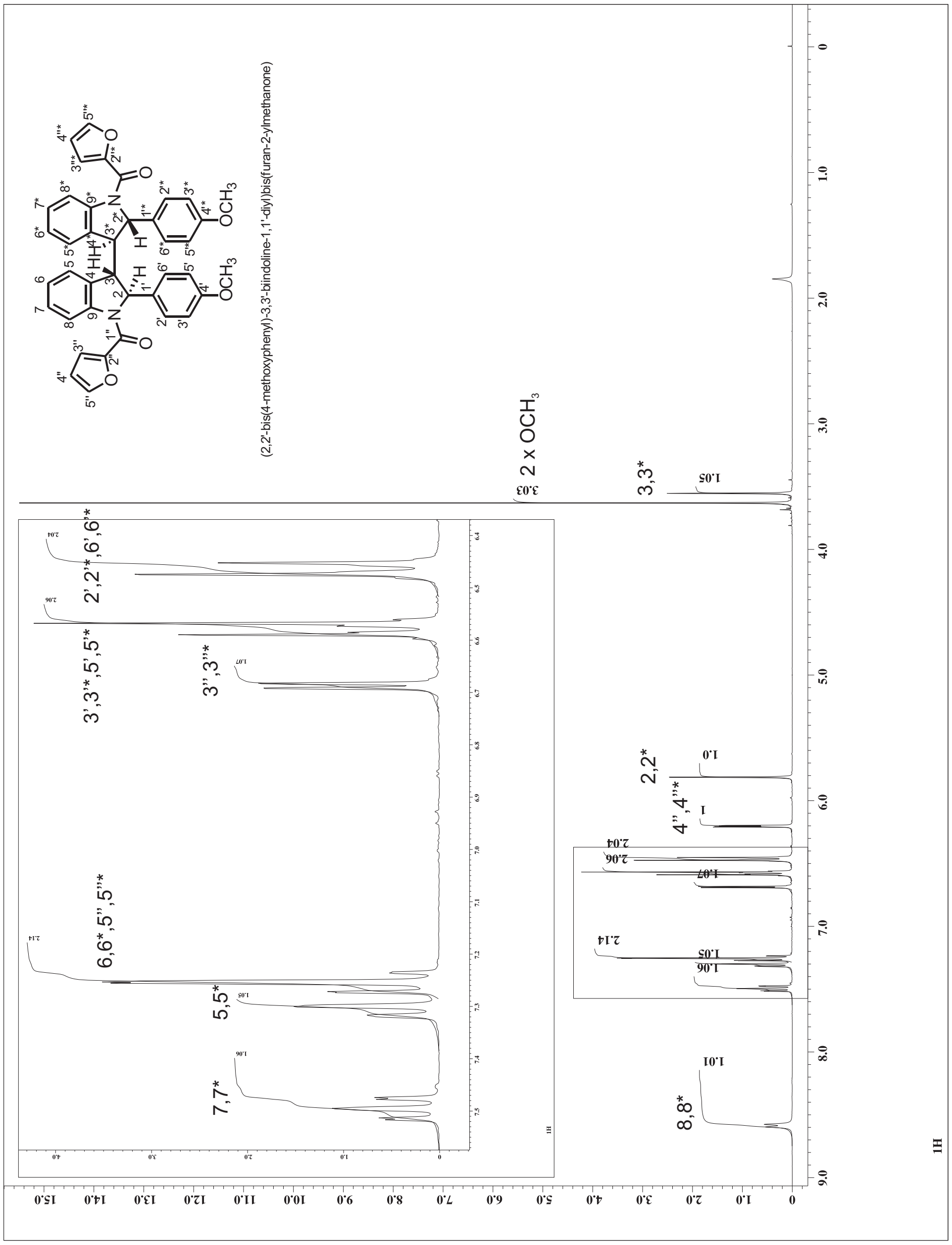

Figure S46: ^{1}H spectrum ($CDCl_3$, 400MHz) of **(±)21b**.

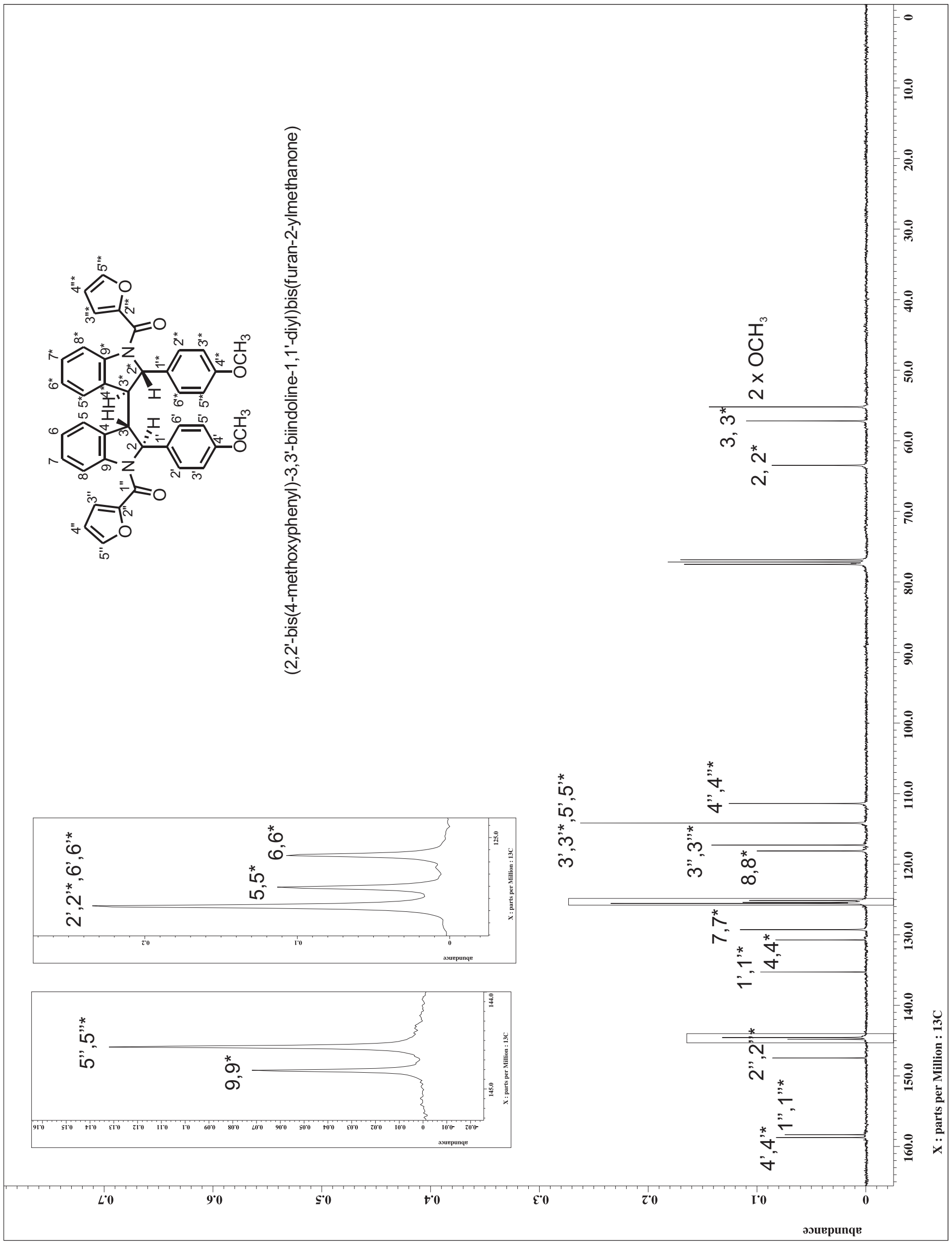

Figure S47: ^{13}C spectrum ($CDCl_3$, 100MHz) of **(±)21b**.

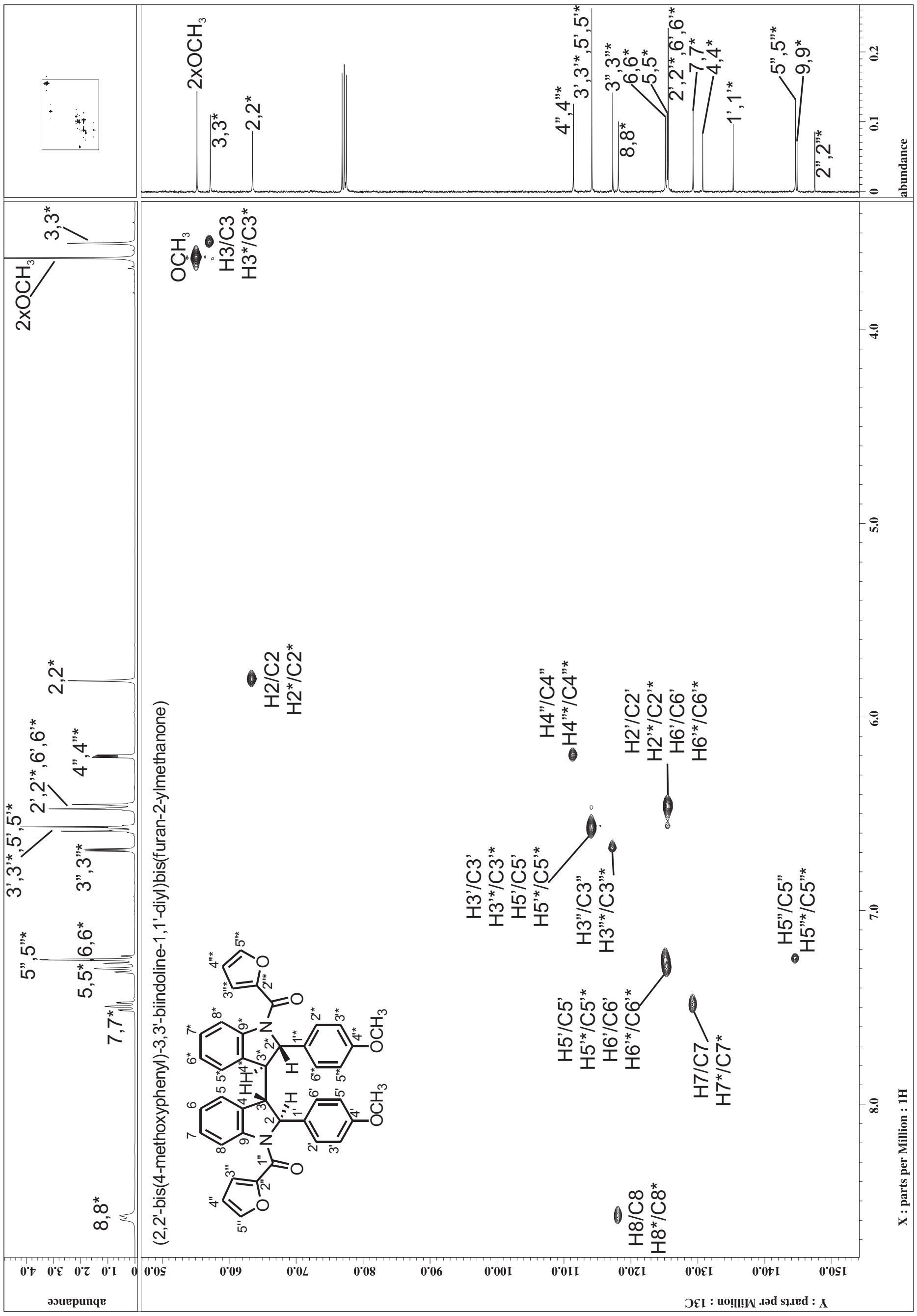

Figure S48: HSQC spectrum ($CDCl_3$, 400MHz) of **(±)21b**.

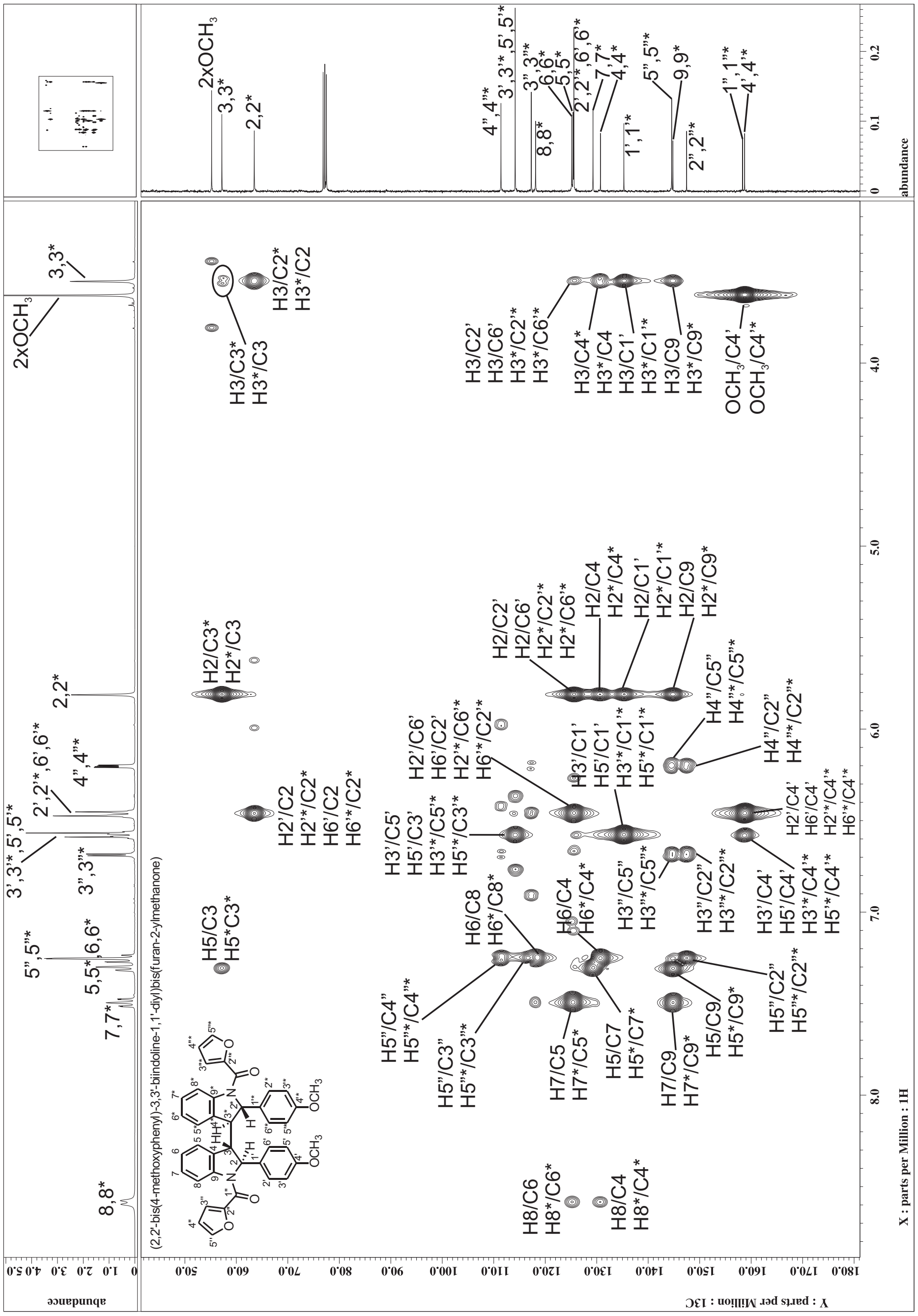

Figure S49: HMBC spectrum ($CDCl_3$, 400MHz) of **(±)21b**.

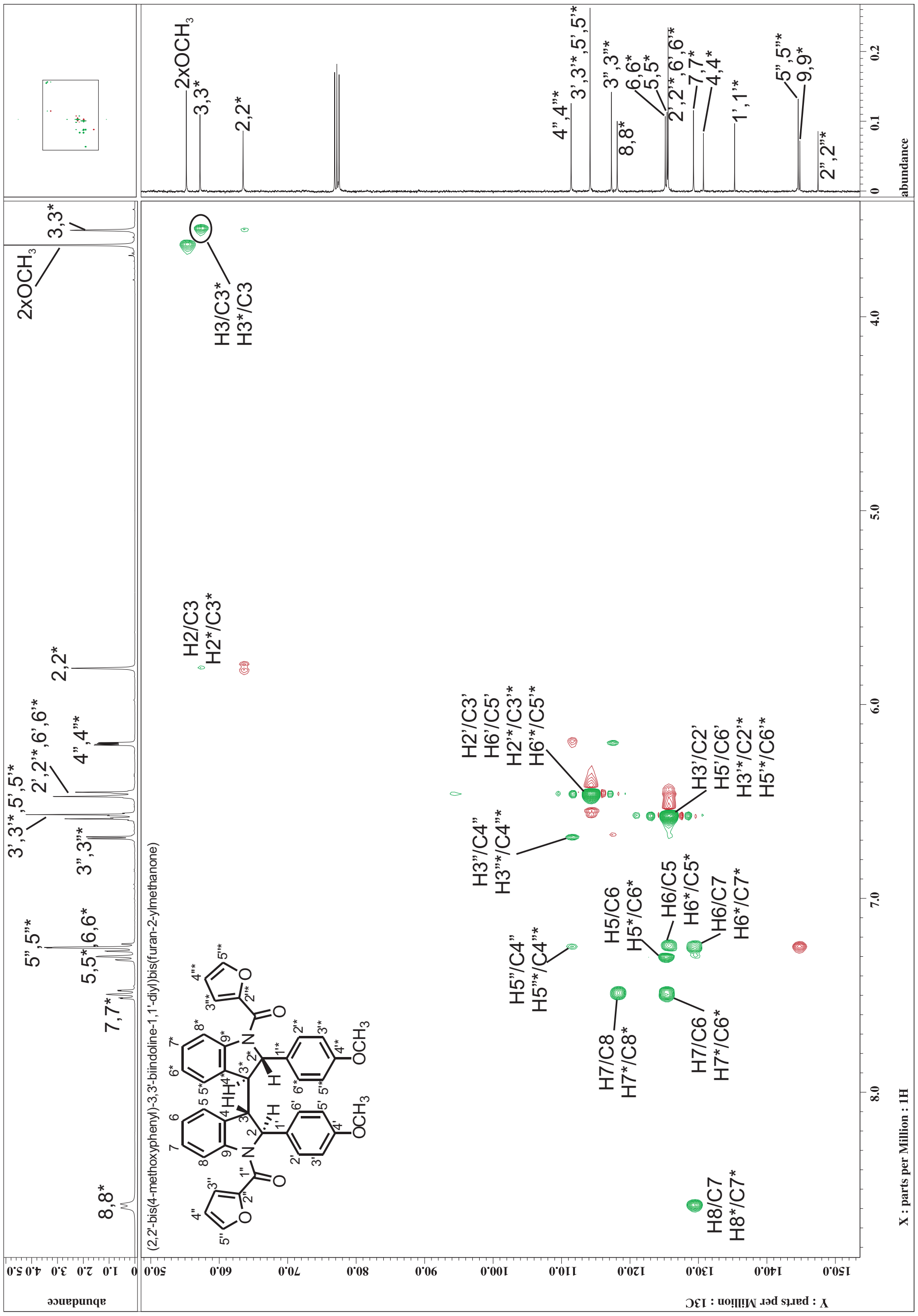

Figure S50: H2BC spectrum ($CDCl_3$, 400MHz) of **(±)21b**.
